# The Relationship Between Running Biomechanics and Running Economy: A Systematic Review and Meta-Analysis of Observational Studies

**DOI:** 10.1007/s40279-024-01997-3

**Published:** 2024-03-06

**Authors:** Bas Van Hooren, Ivan Jukic, Maartje Cox, Koen G. Frenken, Iker Bautista, Isabel S. Moore

**Affiliations:** 1 Department of Nutrition and Movement Sciences, NUTRIM School of Nutrition and Translational Research in Metabolism, Maastricht University Medical Centre+, Universiteitssingel 50, 6229 ER Maastricht, The Netherlands; 2 Sport Performance Research Institute New Zealand (SPRINZ), Auckland University of Technology, Auckland, New Zealand; 3 School of Engineering, Computer and Mathematical Sciences, Auckland University of Technology, Auckland, New Zealand; 4 Institute of Sport, Nursing and Allied Health, University of Chichester, Chichester, UK; 5 Department of Physiotherapy, Catholic University of Valencia, Valencia, Spain; 6 School of Sport and Health Sciences, Cardiff Metropolitan University, Cardiff, UK

## Abstract

**Background:**

Running biomechanics is considered an important determinant of running economy (RE). However, studies examining associations between running biomechanics and RE report inconsistent findings.

**Objective:**

The aim of this systematic review was to determine associations between running biomechanics and RE and explore potential causes of inconsistency.

**Methods:**

Three databases were searched and monitored up to April 2023. Observational studies were included if they (i) examined associations between running biomechanics and RE, or (ii) compared running biomechanics between groups differing in RE, or (iii) compared RE between groups differing in running biomechanics during level, constant-speed, and submaximal running in healthy humans (18–65 years). Risk of bias was assessed using a modified tool for observational studies and considered in the results interpretation using GRADE. Meta-analyses were performed when two or more studies reported on the same outcome. Meta-regressions were used to explore heterogeneity with speed, coefficient of variation of height, mass, and age as continuous outcomes, and standardization of running shoes, oxygen versus energetic cost, and correction for resting oxygen or energy cost as categorical outcomes.

**Results:**

Fifty-one studies (*n* = 1115 participants) were included. Most spatiotemporal outcomes showed trivial and non-significant associations with RE: contact time *r* = − 0.02 (95% confidence interval [CI] − 0.15 to 0.12); flight time *r* = 0.11 (− 0.09 to 0.32); stride time *r* = 0.01 (− 0.8 to 0.50); duty factor *r* = − 0.06 (− 0.18 to 0.06); stride length* r* = 0.12 (− 0.15 to 0.38), and swing time *r* = 0.12 (− 0.13 to 0.36). A higher cadence showed a small significant association with a lower oxygen/energy cost (*r* = − 0.20 [− 0.35 to − 0.05]). A smaller vertical displacement and higher vertical and leg stiffness showed significant moderate associations with lower oxygen/energy cost (*r* = 0.35, − 0.31, − 0.28, respectively). Ankle, knee, and hip angles at initial contact, midstance or toe-off as well as their range of motion, peak vertical ground reaction force, mechanical work variables, and electromyographic activation were not significantly associated with RE, although potentially relevant trends were observed for some outcomes.

**Conclusions:**

Running biomechanics can explain 4–12% of the between-individual variation in RE when considered in isolation, with this magnitude potentially increasing when combining different variables. Implications for athletes, coaches, wearable technology, and researchers are discussed in the review.

**Protocol registration:**

10.17605/OSF.IO/293ND (OpenScience Framework).

**Supplementary Information:**

The online version contains supplementary material available at 10.1007/s40279-024-01997-3.

## Key Points

**Table:** 

Among spatiotemporal variables, ground contact time, flight time, and duty factor showed trivial and non-significant associations with running economy, while a higher step frequency was weakly associated with a better running economy.
Lower vertical displacement and higher vertical and leg stiffness were moderately associated with better running economy, while joint angles at specific instances of the gait cycle, joint angle range of motion, peak vertical ground reaction force, mechanical work variables, and electromyographic muscle activation showed non-significant and often trivial associations with running economy. Nevertheless, some non-significant trends of at least a small magnitude were observed for some outcomes (e.g., co-contraction duration, joint angles at toe-off).
Overall, our findings show that biomechanical variables can explain 4–12% of the between-individual variance in running economy when considered in isolation, with this magnitude potentially increasing when combining different variables.

## Introduction

Running economy (RE) represents the amount of oxygen or energy required to run at a given steady-state speed and is considered an important determinant of running performance, alongside other variables such as the maximum oxygen uptake ($\dot{V}{{\text{O}}}_{2{\text{max}}}$) and the ability to run at a high percentage of $\dot{V}{{\text{O}}}_{2{\text{max}}}$ [1–3]. These three factors can collectively account for approximately 89%–95% of the variance in long-distance running performance [4], or speed at anaerobic threshold [5, 6]. However, RE has been shown to have a stronger association with running performance than $\dot{V}{{\text{O}}}_{2{\text{max}}}$ within homogeneous running populations [7, 8], although there are some conflicting findings [9]. Further support for the importance of RE for running performance is provided by the dominance of East Africans in distance running events, which has often been attributed to their superior RE compared with other ethnicities [10, 11]. Finally, changes in RE have been shown to have strong associations with changes in running (i.e., time-trial) performance in studies that acutely alter RE, for example by shoe wear manipulations [12, 13], as well as long-term studies, in which changes in RE correlate with changes in time-trial performance [14]. While much of the previous work focused solely on male runners, it is likely similar findings would be present in female runners due to limited sex differences in RE when measured at relative intensities [15, 16].

Due to the importance of RE for running performance, knowledge about factors that can (a) influence RE, and (b) be modified to improve RE, is crucial for coaches and athletes, as well as researchers. Several factors have been shown to be associated with RE, such as anthropometrical measures, biochemical aspects, musculotendon properties, and running biomechanics [17–22]. Specifically, longer lower leg length has been associated with better RE in a group of high-level male European distance runners [18]. The proportion of type I and type II fibers has also been associated with RE, although the evidence is often conflicting [17, 19–21, 23]. Finally, several running-related spatiotemporal characteristics, kinematics, and kinetics have also been associated with RE [23–25]. Of these factors, running biomechanics is the only factor that can be modified both acutely (i.e., during a race) and chronically (i.e., over the course of a training program).

Previous work considering how biomechanics influences RE between runners has shown that 54% of the between-individual variation in RE (expressed as mLO_2_∙kg^−1^∙min^−1^) was explained by two kinematics and one kinetic variable [23], whilst others have shown three kinematics to explain 39% of the between-individual variance in RE (expressed as kcal∙kg^−1^∙km^−1^) [24]. In terms of modifying running biomechanics, within-subject changes in running kinematics and kinetics have been reported to explain up to 94% of the changes in RE over a 10-week running program in female runners [26, 27]. Given the modifiable nature of running kinematics and kinetics, coaches and athletes often try to optimize them in an attempt to improve RE and hence running performance. For example, studies have manipulated stride length [28, 29] and ground contact time [30] to show that small adjustments to these characteristics could be beneficial for runners whose self-selected gait deviates from a gait that would mathematically minimize oxygen or energy cost, which would thus improve RE. Similarly, some wearable technologies claim to help enhance performance by attempting to aid runners in modifying factors such as vertical displacement or footstrike pattern, often based on the implicit assumption that there is a common economical running technique for all runners (at least for the modified component) [31].

Although multiple studies have investigated the association between running biomechanics and RE, the evidence is often inconclusive or even conflicting. For instance, while some studies reported rearfoot striking to be associated with a better RE [23, 32], other studies reported fore-/mid-foot striking to be associated with a better RE [24, 33], and yet several studies reported no differences in RE between runners with different footstrike patterns [34, 35]. These conflicting findings may reflect differences in the methods used to assess running biomechanics and RE (e.g., no use of a fixed speed for all participants), or differences due to sampling variation with small sample sizes. However, they could also reflect true differences in the most economical running biomechanics between (groups of) individuals [36]. Although several reviews have covered the relationship between running biomechanics and RE [25, 31, 37, 38], they have also discussed the association of RE with other variables such as footwear or physiological factors. As a result, such reviews have provided a limited detailed critical appraisal of conflicting findings regarding the association between running biomechanics and RE. Further, they were all narrative reviews that had no systematic search and therefore could have missed relevant studies. A systematic review can highlight best practices in data collection and limitations, both of which may be used by future studies to further investigate associations between running biomechanics and RE. Additionally, a meta-analysis can weigh studies according to their precision and thereby provide a more informative estimate of the association between running biomechanics and RE. As such, a systematic review with meta-analysis that (i) provides an updated and comprehensive overview of the associations between running biomechanics and RE, and (ii) discusses potential reasons for conflicting findings would be beneficial. Therefore, the primary aim of this systematic review and meta-analysis was to synthesize the available evidence on the association between running biomechanics and RE as investigated in observational studies. Such evidence is important to inform coaches, athletes, researchers, and developers of wearable technologies on running biomechanics modification strategies, ultimately allowing for more effective improvements in RE.

Studies that investigate the correlation between RE and running biomechanics at different speeds do not always report consistent associations across speeds (e.g., [39, 40]). Further, it is well known that shoes can influence RE [41–43] and running biomechanics [44, 45] and shoe standardization across individuals may therefore impact the correlations between RE and running biomechanics. Similarly, the method used to express RE (i.e., oxygen or energy cost), and normalization of RE for resting or standing oxygen/energy expenditure may impact the established correlations. As a secondary aim, we therefore also explored if the association between running biomechanics and RE was modulated by running speed, the use of standardized shoes, the method used to express RE (i.e., oxygen or energy cost), and normalization of RE for resting or standing oxygen/energy expenditure. Further, given the potential influence of anthropometric characteristics [46–49] and age [50–52] on RE and running biomechanics, we also explored whether sample homogeneity for height, mass, and age affected the magnitude of the correlations.

## Methods

### Registry of Systematic Review Protocol

A systematic review of the literature was performed using guidelines in the Cochrane Handbook for Systematic Reviews of Interventions (version 6.0) and following the checklist for the Preferred Reporting Items for Systematic reviews and Meta-Analyses 2020 (PRISMA) [53]. The protocol was prospectively registered on the OpenScience Framework (https://osf.io/293nd/). Registration occurred after searches had been conducted, but before screening was completed.

### Eligibility Criteria

To be included, studies had to (i) be cross-sectional studies that determined associations between running biomechanics and RE, or compared running biomechanics between two or more groups differing in running economy, or compared running economy between two or more groups differing in running biomechanics (e.g., footstrike comparison studies), during level (0 and 1% incline), constant-speed motorized treadmill, or overground running at sub-maximal speeds (i.e., respiratory exchange ratio < 1.0 or below lactate threshold/respiratory compensation point); (ii) be performed among healthy, non-injured humans between 18–65 years; (iii) measure biomechanical variables (spatiotemporal, kinematics, kinetics, and muscle activity outcomes were all included); (iv) be written in English; and (v) measure RE using respiratory gas analysis (both oxygen and energetic cost and linear or allometrically scaled data were included). Grey literature such as conference abstracts and theses were included only if they provided sufficient methodological details, or if the authors provided this upon request. Data on running above the anaerobic threshold/respiratory compensation point, sprinting (defined here as > 25 km∙h^−1^ or > 7 m∙s^−1^ [54]), barefoot running, running with orthopedic inserts, musculoskeletal modeling studies, running in a fatigued state, and running with additional mass were excluded. We did not apply restrictions to the training level of the sample.

### Information Sources

Three electronic databases (MEDLINE via PubMed, Web of Science, and Embase) as well as two pre-print servers (SportRxiv and BioRxiv) were searched. The searches covered all dates of available literature, with the date of the last search being September 30, 2020. No limits were applied for language within each database to prevent exclusion of articles that were not assigned a language. Search alerts were created to monitor any new search results after the date of the last search up to April 1, 2023. Any articles identified by this search were assessed by two researchers (BVH and KF) for eligibility. One researcher (BVH) double-checked the included papers from the systematic search and modified the eligibility criteria to limit the scope of the review, for example, by only including studies that assessed correlations with all participants running at the same speed as opposed to a percentage of their ventilatory threshold or $\dot{V}{\text{O}}_{2{\text{peak}}}$. Hand searching of reference lists and forward citation searching of included studies was also used to identify articles. An additional narrative search was performed on February 1, 2022, for studies that compared both RE and biomechanics with participants running in different shoes. Although we were not interested in the effect of shoes, studies comparing running economy and running biomechanics between different shoes collected all information to compute correlations between running biomechanics and RE. Therefore, the authors of these studies were emailed to request averaged correlational data across shoes between the measured running biomechanics and RE so these data could also be included in the analyses.

### Search Strategy

A PICO systematic search strategy was developed for PubMed together with a research librarian, and using the Word Frequency Analyser tool (http://sr-accelerator.com/#/help/wordfreq) to suggest potentially relevant search terms [55]. The Research refiner tool (https://ielab-sysrev2.uqcloud.net/) was subsequently used to optimize the sensitivity and specificity of the search, while the Polyglot Search Translator Tool (https://sr-accelerator.com/#/polyglot) was used to adapt the search to other databases [56, 57]. The final search consisted of terms for running, running economy, and running biomechanics. The search string used for all databases is reported in Supplementary File S1 of the Electronic Supplementary Material (ESM).

### Study Selection

Duplicate references were first removed using an online deduplicate tool for systematic reviews (https://sr-accelerator.com/#/libraries/dedupe) [58] and subsequent manual methods. Two authors (BVH and KF) then independently screened titles and abstracts to determine initial eligibility using systematic review software (Rayyan) [59]. Blinding of authors was used to reduce bias during this process. Finally, the authors reviewed the full texts of all articles to determine their eligibility for inclusion based on the eligibility criteria. Disagreements in eligibility decisions were resolved through discussion, or with a third reviewer (IM) when required.

### Data Collection Process

Data extraction was completed independently and in duplicate by four authors (KF, IB, BVH, MC) using a standardized form that was pilot tested on five randomly selected included studies and refined accordingly through discussions with BVH and IM. The data were then merged by two authors (BVH, MC) and any discrepancies in the extracted data were resolved through discussion. Extracted data from each full-text article included (i) study identification information; (ii) study design; (iii) sample size; (iv) sex and nationality/ethnicity; (v) age, height, and body mass; (vi) running ability (e.g., weekly distance, personal best times, and RE); (vii) running surface; (viii) data collection equipment and procedures (e.g., wearable device or 3D motion capture, gas exchange equipment); (ix) running speeds; (x) footwear; (xi) data analysis approaches (e.g., verification of steady-state, corrections or no corrections of RE for resting oxygen/energy expenditure); (xii) correlations between biomechanics and RE for correlational studies, or (xiii) means and standard deviations for relevant outcome measures; and (xiv) an exact *p*-value, *t*-value, or confidence intervals when a study compared RE/biomechanics between groups differing in biomechanics/RE, respectively. If insufficient data were reported, or when more metrics were measured than reported in the results (e.g., studies that assessed duty factor but did not report correlations for contact time and flight time), the corresponding authors were contacted by email. If the corresponding author did not respond, we contacted other authors of the paper and also used different contact methods (e.g., ResearchGate). When data were not presented in tables or text and when authors did not provide the requested data, these were extracted from figures using WebPlot Digitizer (Web Plot Digitizer, V.4.1. Texas, USA) [60] where possible.

### Risk-of-Bias Assessment

Although several risk-of-bias tools are available [61–63], most tools are developed for risk-of-bias assessment of intervention studies and therefore contain several criteria that are not relevant to the observational studies included in this review. Therefore, we modified the risk-of-bias tool developed by Hoy and colleagues [64] and used this to perform a risk-of-bias assessment independently by three authors (IJ, KF, BVH). More information on the criteria used in risk-of-bias assessment can be found in Supplementary File S2 of the ESM. The risk of bias was assessed based on the information reported in the published paper and not on information provided by the authors, except for information regarding steady-state verification as this was often missing in the papers. Disagreements in risk-of-bias assessment were resolved by discussion before the scores were merged into a spreadsheet. The mean kappa agreement between the authors was 0.95 (nearly perfect). The risk of bias was considered in the interpretation of the results by applying the Grading of Recommendations Assessment, Development and Evaluation (GRADE) system [65]. Briefly, the overall quality was initially rated as high and downgraded one level to moderate, low, or very low for each of the following limitations: total sample size < 100 participants (imprecision), high (*I*^2^ > 50%) statistical heterogeneity (inconsistency), > 50% of studies in the meta-analysis had one or more risk-of-bias items assessed to be high risk (risk of bias). For individual study outcomes, we used the same criteria but rated the risk for statistical heterogeneity down if there was only one study reporting on a specific outcome.

### Statistical Analysis

#### Effect Size and Synthesis of Studies Providing Correlations

Pearson correlation coefficients between running biomechanics and RE were considered the primary effect size of interest. If studies reported a Spearman’s rank correlation, we converted this to a Pearson correlation using Eq. 1 as Spearman’s correlations are typically smaller than Pearson correlations [66]:

1 $$r=2{\text{sin}}({r}_{s}\frac{\pi }{6})$$

where *r*_s_ is Spearman’s rank correlation.

The sampling distribution of (Pearson) correlation coefficients is increasingly non-normal (i.e., skewed) with stronger correlations because it is bound from − 1 to 1 [67]. Correlations were therefore transformed to Fisher’s *z* as detailed previously [67] to better approximate a normal distribution. The *z*-transformed correlation coefficient was then meta-analyzed to obtain a weighted point estimate with 95% confidence intervals, and these were back-transformed into a Pearson correlation coefficient using an integral *z*-to-*r* transformation [68] to aid interpretation. Correlations were interpreted as < 0.1 trivial; 0.1–0.29 small; 0.30–0.49 moderate; 0.5–0.69 large; 0.7–0.89 very large; 0.9–0.99 nearly perfect [69].

The synthesis of *z*-scores across studies was done using a random-effects model, with a separate random-effects meta-analysis being performed when two or more studies reported on the same outcome. A substantial proportion of studies included in this review provided two or more correlation coefficients (e.g., at multiple speeds). Two effects from the same study (e.g., correlation coefficients between the two variables at different speeds) are likely more similar than two effects from two different studies due to the use of the same participants and data collection and analysis procedures within each study. The inclusion of multiple effects from the same study would therefore violate the assumption of independence in traditional meta-analyses. To account for this, we conducted a three-level meta-analysis (i.e., a multi-level model). By using a three-level structure we accounted for three different variance components distributed over the three levels in the model. This included sampling variance of the extracted effect sizes at level one, variance between the extracted effect sizes within the same study at level two, and variance between studies at level three. We used cluster-robust variance estimation methods [70] with small-sample adjustments [71] to adjust the within-study standard errors for correlations between effect sizes. To do so, the method required an estimate of the mean correlation between all pairs of within-study effect sizes (*ρ*), which was used to correct the between-study sampling variance (*τ*^2^) for statistical dependencies [72]. Since information about the sampling correlations among effect sizes was limited, this correlation was set to 0.6 [72]. Sensitivity analysis with correlations of 0.4 and 0.8 showed no differences in the outcomes of the meta-analyses.

The inverse of the standard error was used to determine the weight (i.e., contribution) of each effect (i.e., correlation coefficient) in the meta-analysis. Within the multi-level meta-analysis implemented, the standard error (and thus weight) of each study was determined by a combination of within- and between-study heterogeneity in effect sizes, the correlation between effect sizes within each study, and the sample variance of each effect size as described in Eq. 2 [73]:

2 $${w}_{j}=\frac{{k}_{j}}{{k}_{j}{\widehat{t}}^{2}+{k}_{j}\rho {\sigma }_{j}^{2}+{\widehat{\omega }}^{2}+(1-\rho ){\sigma }_{j}^{2}}$$

where ${w}_{j}$ is the weight of study *j*, ${k}_{j}$ the number of effect sizes within study *j*, ${\widehat{t}}^{2}$ the between-study heterogeneity, $\rho$ the correlation between the within-study effects, ${\widehat{\omega }}^{2}$ the within-study heterogeneity, and ${\sigma }_{j}^{2}$ the sample variance of each effect. The sample variance of each effect (i.e., of the Fisher’s *z*-transformed correlation) was in turn directly proportional to the sample size as shown in Eq. 3 [67]:

3 $${\sigma }_{j}^{2}=\frac{1}{n-3}$$

where *n* is the sample size.

Within this weighting procedure (Eq. 2), a large between-study variation in effect sizes will result in relatively more equal weights given to different studies. Further, when there is large within-study variability in effect sizes, relatively more weight will be given to studies that provide multiple effect sizes because the average estimate from such a study will yield a more accurate estimate of the real effect than a study that provides only one effect. This weight will, however, also depend on the correlation assumed between the effect sizes. Specifically, if the correlation between within-study effect sizes is high, averaging highly correlated estimates does not substantially improve the precision relative to using one effect size. Finally, effect sizes with small variance (i.e., large sample size, see Eq. 3) will receive more weight than effect sizes with larger variance.

All model parameters were estimated using the restricted maximum likelihood estimation method. Tests of individual coefficients in all models, and their corresponding confidence intervals, were based on a *t*-distribution. Multilevel meta-analyses and meta-regressions were performed in R (version 4.2.0, R Foundation for Statistical Computing) [74] using the *metafor* package [74], whereas the *clubSandwich* package [72] was used to implement the robust methods with correlated and hierarchical effects.

The heterogeneity of the correlations across studies was assessed using the *I*^2^ statistic obtained from the multi-level model. Briefly, the variance components of the pooled correlation coefficient were decomposed into sampling variance of the observed correlations (level 1), and variance within (level 2) and between studies (level 3). This *I*^2^ (level 3) represents the percentage of the total variation in estimated effects across studies due to heterogeneity rather than chance and was interpreted as small (*I*^2^ < 25%), moderate (*I*^2^ = 25–49%), and high (*I*^2^ > 50%) [75]. We only report and use the *I*^2^ (level 3) for the GRADE criteria (see Sect. 2.7).

Meta-regressions were performed when at least ten effects (i.e., correlations) were available for an outcome [67, 76]. Meta-regressions were performed with running shoes (i.e., standardized vs non-standardized shoes), RE units (i.e., oxygen or energy cost), and normalization of RE (i.e., corrected for resting or standing oxygen/energy expenditure) as categorical outcomes when at least two studies reported on each moderator. Categorical moderators were dummy coded (e.g., oxygen cost = 1, energy cost = 0) to allow the regression coefficient to be interpreted as the difference in effect size between two levels of the moderator. If studies reported correlations between running biomechanics and energy cost expressed as caloric units and oxygen cost, we used the caloric units for all analyses. We performed a sensitivity analysis to investigate the difference in correlations between running biomechanics and RE with RE expressed as oxygen or caloric cost to investigate the impact of this decision (Supplementary File S3, see ESM). When no specification of shoe standardization was reported, we assumed participants ran in non-standardized shoes. Similarly, RE was assumed to be not corrected for resting or standing oxygen/energy expenditure if this was not specifically reported. Meta-regressions were performed with speed, and the coefficient of variation in height, mass, and age as continuous outcomes. The coefficients of variation for height, mass, and age were determined from the reported mean and standard deviation in each study and were used as continuous outcomes because the homogeneity of a group on these outcomes could affect the magnitude of the correlations given the potential influence of anthropometric characteristics [46–49] and age [50–52] on RE and running biomechanics. If there were sufficient studies to perform a multi-variable meta-regression (i.e., at least 10 effects per moderator), we combined variables in the following fixed order: (a) RE units, (b) shoe standardization, (c) speed, (d) normalization of RE, while always ensuring at least 10 effects were available per moderator.

Leverage, outlier, and influential case diagnostics were conducted for all meta-regression models by examining hat, Cook’s distance, and studentized residuals [77–79]. Cases exceeding three times the mean value for hat and Cook’s distance, as well as an absolute studentized residual > 3, were considered influential. These red-flagged estimates (i.e., correlation coefficients) were then dropped from the dataset, and meta-regression models were refitted without them. If the interpretation of the meta-regression model did not change after excluding influential estimates, the original model was retained. In contrast, if the interpretation of a given meta-regression model changed as a consequence of removing influential cases, the model without influential cases was retained and reported instead.

#### Effect Size and Synthesis of Studies Providing Between-Group Differences

Some studies compared running biomechanics and RE between two groups running at a similar speed and did not report a correlation coefficient, but instead reported mean and standard deviations for RE and some biomechanical outcomes of each group, as well as a statistic representing the between-group difference. Specifically, six studies reported or provided RE data for runners running with a rearfoot or mid/forefoot strike. As the RE data were reported in different units, standardized mean differences for independent groups were computed using procedures outlined by Borenstein et al. [67]. Briefly, the RE data in the rearfoot strike group were subtracted from the mid/forefoot strike group to determine the mean difference, which was then divided by the pooled within-group standard deviation to determine the standardized mean difference. The resulting standardized mean differences and their variance were corrected for small sample bias using a correction factor [67].

After the effect sizes (i.e., standardized mean differences) for each comparison were calculated, a meta-analysis was performed using similar procedures as for the correlational data reported in Sect. 2.8.1. Briefly, a cluster-robust variance estimation method [70] with small-sample adjustments [71] was used to adjust the standard error for the overall standardized mean difference, with clustering at the study level. The sampling correlation between the effect sizes was assumed to be 0.6. In addition, the inverse of the standard error was used to weigh each effect as detailed in Eq. 2. Note, however, that the sample variance of each effect was determined for standardized mean differences of independent groups (as opposed to correlations) as detailed by Borenstein and colleagues [67]. Finally, the model’s parameters were estimated using the restricted maximum likelihood estimation method and *p*-values and confidence intervals were based on a *t*-distribution.

#### Data Reduction

The variability in designs and outcomes among eligible studies required several decisions to ensure the data could be appropriately combined for meta-analysis. These decisions are specified in Supplementary File S4 (see ESM). Most importantly, joint or segment angles were expressed in the same reference frame (e.g., higher values representing higher flexion in all studies for a given outcome) so that correlations were also directionally consistent. Two authors (BVH and IM) were involved in checking the reference frame assignment to ensure accuracy. Further, step and stride frequency were combined in one analysis, and we refer to stride frequency throughout the paper to ensure consistency. A similar approach was used for step and stride length. Further, while most studies reported vertical oscillation during a stride or step (i.e., including the flight phase), some studies reported only on the stance phase vertical displacement. Because step vertical oscillation and stance vertical displacement have highly similar correlations with RE [24], we combined all outcomes in one analysis and refer to these as vertical oscillation for consistency. While different methods were used to classify footstrike patterns (e.g., foot–ground angle, footstrike index), we will collectively refer to footstrike angle in Sect. 3 and Sect. 4 as this was the dominant method used in the included studies.

### Publication Bias

Publication bias was not assessed because there was only a small number of studies included in most meta-analyses and we did not see any reason why studies reporting no correlation between RE and biomechanics would be less likely to be published than studies finding a significant correlation. Additionally, we included studies that did not directly aim to assess the relationship between running biomechanics and RE. Therefore, these studies are unlikely to be affected by publication bias. Finally, we also reduced the potential impact of publication bias by checking the consistency between the measured biomechanical outcomes, and reported biomechanical outcomes in Sect. 3.

## Results

### Search Results

**Fig. 1:**
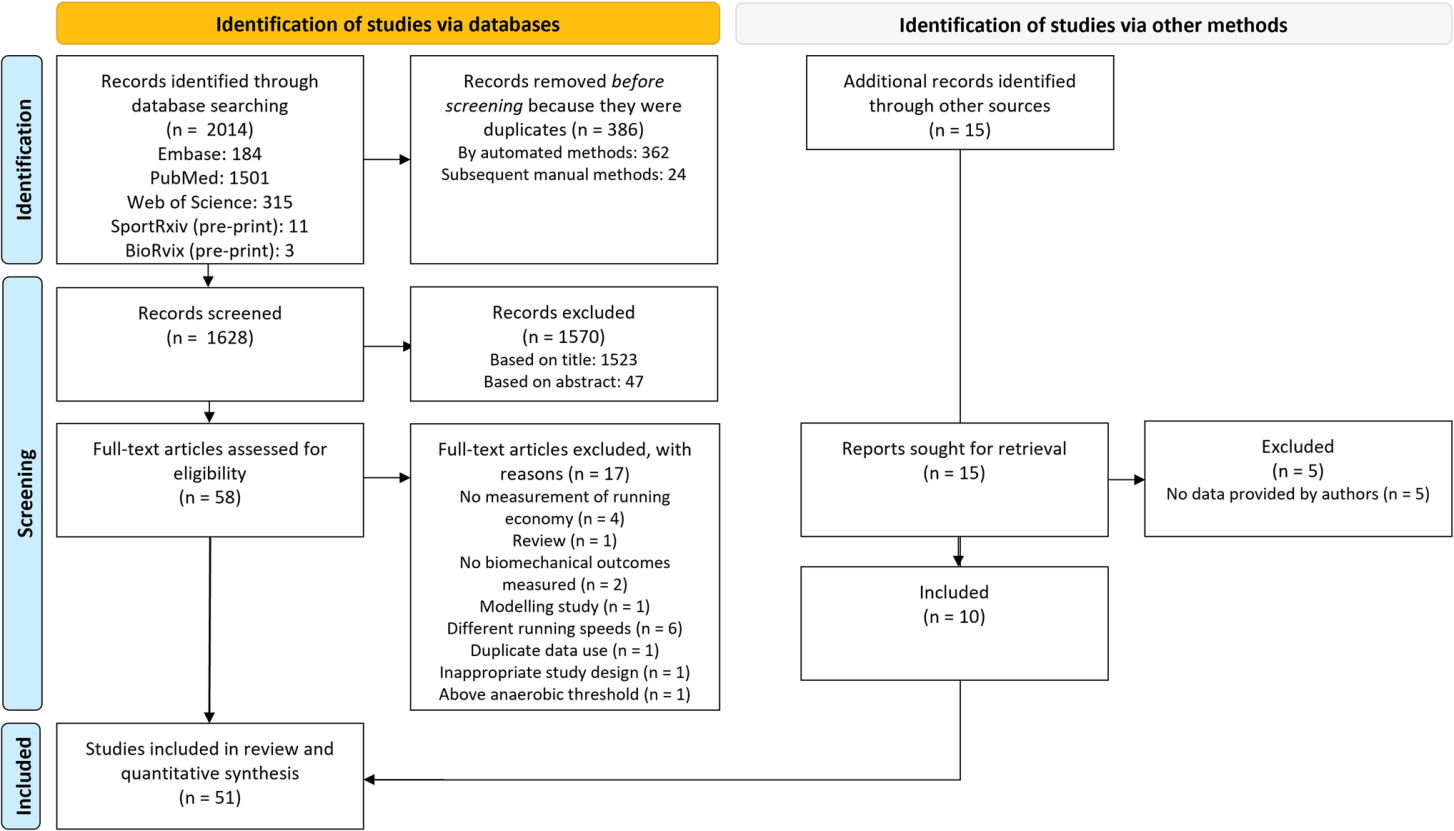
PRISMA flow-diagram

The initial literature search yielded 2014 records through electronic databases (Fig. 1). Title and abstract screening resulted in exclusion of 1570 records. After screening 58 records for inclusion/exclusion criteria, 17 records were excluded, resulting in 41 articles being identified from the original search. A combination of forward citation searching for articles that passed title/abstract screening and monitoring of newly published literature using the search alerts and regular manual checking of relevant journals yielded an addition 15 records for consideration in the review. Five of these were subsequently excluded because the authors did not provide the requested data, resulting in a total number of 51 articles included in the review.

### Study Characteristics

Detailed study characteristics are reported in Table 1. All 51 studies included in this review provided either correlations between running biomechanics and RE (38 studies), compared RE between groups differing in running biomechanics (e.g., footstrike angle; 5 studies [32, 34, 35, 80, 81]), compared running biomechanics between groups differing in RE (5 studies [23, 82–85]), or provided both correlations and between-group comparisons (3 studies [81, 86, 87]). The total number of participants in the included studies was 1115 (904 males, 227 females). Note that three studies used the same sample [88–90], but analyzed different biomechanical outcomes. The sample size of these studies was counted only once for the overall sample size calculation. Of the 51 included studies, 35 included only males, 2 only females, 13 both males and females (only one presented sex-disaggregated data), and one did not specify the sex of included participants. Fifty studies recruited participants that were runners or physically active in other sports, and one study did not specify the physical activity of the participants [40]. Running speeds used for RE assessment ranged from 2.22 m∙s^−1^ [40, 91] to 5.56 m∙s^−1^ [92]). Sixteen studies standardized shoe wear, while 38 studies did not, or at least did not explicitly report that they had.

**Table 1:** Study characteristics

Study reference; design within our meta-analysis	Participant characteristics M/F; mean ± SD age (years); height (cm); mass (kg); ethnicity	Running ability Subjective description of range of running abilities; mean ± SD personal best times; weekly training distance (km∙w^−1^); running experience (years); $\dot{V}{\text{O}}_{2\max }$ (mL∙kg∙min^−1^); running economy (in different units)	Assessment of running economy Equipment used; speed/relative intensity; duration of run; period for data analysis; verification steady-state	Expression of running economy Units; equation used to determine energy cost from $\dot{V}{\text{O}}_2$ (if applicable); resting or standing $\dot{V}{\text{O}}_2$ or energy expenditure (EE) subtracted from RE (if any)	Assessment of running biomechanics Time of measurement (concurrent with running economy or separate); equipment used; surface; shoes; leg used for data collection (if applicable)	Biomechanical variables measured
Adelson et al. [105]; correlational	19/11; 28 ± 5; 177 ± 9; 73.1 ± 13.4; not reported	Recreational runners; no PBs reported; 30.58 ± 20.92 km∙w^−1^, no running experience or $\dot{V}{\text{O}}_{2\max }$ reported; RE (in mLO_2_∙kg^−1^∙min^−1^) at 2.68 m∙s^−1^ was 34.4 ± 3.8	Stationary gas analyzer (TrueOne 2400, Parvo Medics, East Sandy, UT, USA); 2.68 m∙s^−1^; 8 min; last 2 min used for data analysis; post-exercise blood lactate values < 3 mmol∙L^−1^	mLO_2_∙kg^−1^∙min^−1^; n.a.; no subtraction reported	Concurrent; instrumented treadmill, 1000 Hz; treadmill; shoes not standardized; both legs captured	Vertical GRF; Step frequency
Arampatzis et al. [82]; groups differing in RE	28/0; 28.1 ± 4.5; 182 ± 6; 76.8 ± 6.7; not reported	Recreational runners competing locally; no PBs reported; 40–120 km∙w^−1^, no running experience or $\dot{V}{\text{O}}_{2\max }$ reported; RE (in mLO_2_∙kg^−1^∙min^−1^) per speed (m∙s^−1^) and per group High economy group 3.0 = 37.42 3.5 = 43.59 4.0 = 48.93 Moderate economy group 3.0 = 39.86 3.5 = 45.71 4.0 = 51.98 Poor economy group 3.0 = 44.38 3.5 = 49.85 4.0 = 58.93	Stationary breath-by-breath gas analyzer (Jaeger Oxycon α, Hoechberg, Germany); 3.0, 3.5, 4.0 m∙s^−1^; 15 min; last 4 min used for data analysis; visual inspection of $\dot{V}{\text{O}}_2$, and blood lactate sampling	mLO_2_∙kg^−1^∙min^−1^; n.a.; no subtraction reported	Concurrent; high-speed 2D video camera at 250 Hz; treadmill; shoes not standardized; left leg	Sagittal-plane hip, knee and ankle angles and range of motions; stride frequency, contact and swing time, and duty factor
Ardigo et al. [80]; groups differing in biomechanics	8/0; 23.9 ± 4.2; 177 ± 4.5; 68.4 ± 5.9; not reported	Recreational runners; no PBs reported, no weekly training distance, running experience, or $\dot{V}{\text{O}}_{2\max }$ reported; RE (in mLO_2_∙kg^−1^∙min^−1^) per speed (m∙s^−1^) 2.50 = 34.07 2.78 = 35.56 3.06 = 38.53 3.33 = 42.02 3.61 = 44.92 3.89 = 48.73 4.17 = 50.99	Stationary gas analyzer (SensorMedics MMC 4400tc); 7 speeds: 2.50, 2.78, 3.06, 3.33, 3.61, 3.89, and 4.17 m∙s^−1^; 5 min; last min used for data analysis; visual inspection of $\dot{V}{\text{O}}_2$, and RER < 1.0	mLO_2_∙kg^−1^∙min^−1^; n.a.; no subtraction reported	Concurrent; four cameras at 100 Hz; treadmill; shoes not reported; leg used not reported	Step frequency; step length; mechanical work
Barnes et al. [94]; correlational	39/24; males: 20.8 ± 2.8; 177 ± 6; 67.8 ± 6.8; not reported Females 20.5 ± 2.1; 164.8 ± 4.2; 55 ± 5.5; not reported	Male and female distance runners competing at collegiate or national level in events from 800 m to 10 km with 27 qualifying for national championships; no PBs reported; males 97.2 ± 21, females 74.2 ± 12.7 km∙w^−1^; males 6.9 ± 2.9, females 6.9 ± 2.1 years; $\dot{V}{\text{O}}_{2\max }$ (in mLO_2_∙kg^−1^∙min^−1^) males 68.7 ± 4.8, females 59.9 ± 2.5; RE not reported	Stationary gas analyzer (TrueOne 2400, Parvo Medics, Salt Lake City, UT, USA); 3.89 m∙s^−1^; unclear duration; last min used for data analysis; visual inspection of $\dot{V}{\text{O}}_2$, and RER < 1.0	mLO_2_∙kg^−1^∙min^−1^; n.a.; no subtraction reported	Concurrent; treadmill; shoes not reported; not reported	Stride frequency; stride length; contact time; flight time
Beck et al. [81]; correlational and groups differing in biomechanics	15/0; 25.4 ± 5.7; 177 ± 6; 67.6 ± 6.1; not reported	Males able to run 5 km under 25 min; no PBs; weekly training distance; running experience; or $\dot{V}{\text{O}}_{2\max }$ reported; RE (in J∙kg^−1^∙min^−1^) at 3.5 m∙s^−1^ was 12.78	Stationary gas analyzer (TrueOne 2400 Parvo Medics, Sandy, UT, USA); 3.5 m∙s^−1^; 5 min; last 2 min used for data analysis; ensured RER < 1.0	J∙kg^−1^∙min^−1^; Péronnet equation; standing EE subtracted from RE	Separate; 3D motion capture (200 Hz) and instrumented treadmill (1000 Hz); standardized shoes; both legs used for data collection	RE in rearfoot and mid/front foot strikers; contact time; step frequency; vertical GRF peak; braking GRF; propulsive GRF
Besson et al. [95]; correlational	Experienced group: 11/10; males: 35 ± 6; 177 ± 7; 70 ± 7; not reported, females: 35 ± 8; 165 ± 4; 55 ± 4; not reported Elite group: 10/10; males: 31 ± 8; 177 ± 7; 66 ± 6; not reported, females: 29 ± 4; 160 ± 7; 49 ± 6; not reported	Experienced: training at least 3 ×/week for the past 12 months and training for trail competition, Elite: ITRA performance index > 700 (females) or 825 (males); no PBs reported; avg monthly training distance during past 12 months experienced: 34 ± 10 (males) and 38 ± 19 (females), elite: 44 ± 11 (males) and 57 ± 21 (females) km; no running experience, $\dot{V}{\text{O}}_{2\max }$ or RE reported	Portable breath-by-breath gas exchange analyzer (Metamax 3B, Cortex, Leipzig, Germany); 2.78 and 3.89 m∙s^−1^; 4 min; last 0.5 min used for data analysis; visual inspection of $\dot{V}{\text{O}}_2$, and RER < 1.0	J∙kg^−1^∙m^−1^; Péronnet equation; no subtraction reported	Concurrent; optical measurement system; treadmill; shoes not reported; leg not reported	Step frequency; vertical stiffness; leg stiffness; contact time, flight time; duty factor
Bohm et al. [96]; correlational	12/11; intervention group: 9/4; 29 ± 5; 178 ± 8; 73 ± 8; not reported Control group: 3/7; 31 ± 3; 175 ± 10; 70 ± 11; not reported	Recreational runners; no PBs reported; ≥ 2 weekly running sessions; no running experience or $\dot{V}{\text{O}}_{2\max }$ reported; RE (in W∙kg^−1^) intervention group: pre-test 10.6 ± 0.6; control group: pre-test 11.2 ± 1.0	Not reported; 2.5 m∙s^−1^; 8 min; last 3 min used for data analysis; visual inspection of $\dot{V}{\text{O}}_2$, and RER < 1.0	W∙kg^−1^; Péronnet equation; no subtraction reported	Not reported; 3D motion at 250 Hz, plantar pressure by pressure plate at 120 Hz; treadmill; shoes not reported; right leg used for data collection	Maximum plantar flexion moment; Achilles tendon stiffness; strike index; contact time; flight time; step frequency; ankle and knee joint angles
Craighead et al. [109]; correlational	Not reported; intervention group: 43.1 ± 15.8; 172 ± 7.9; 70.9 ± 13.1; not reported Control group: 37.6 ± 10; 168 ± 8.9; 68.1 ± 12.2; not reported	Healthy recreational runners; no PBs reported; running ≥ 3 days∙week^−1^, ≥ 5 km per session at a self-reported speed between 2.7 and 3.0 m∙s^−1^; > 2 years running experience; no $\dot{V}{\text{O}}_{2\max }$ reported; RE (in mLO_2_∙kg^−1^∙km^−1^) pre-test intervention group 206.5 ± 18.5 and control group 199.3 ± 19.5	Stationary gas analyzer (TrueOne 2400 Metabolic Measurement System, Parvo Medics, Sandy, UT, USA); 2.8 m∙s^−1^; 15 min; last 5 min used for data analysis; visual inspection of steady-state in $\dot{V}{\text{O}}_2$^b^	mLO_2_∙kg^−1^∙km^−1^; n.a.; no subtraction reported	Concurrent; high-speed 2D video camera; treadmill; shoes not standardized; not reported	Stride frequency; stride length, thigh angle at IC; knee angle at IC; ankle angle at IC; knee angle at midstance; max knee angular velocity; heel to greater trochanter distance; vertical displacement of CoM
Di Michele and Merni [34]; groups differing in biomechanics	14/0; rearfoot: 25.0 ± 2.8, midfoot: 25.3 ± 2.4; rearfoot 180.1 ± 5.1, midfoot 175.3 ± 5.2; rearfoot 69.6 ± 4.0, midfoot 64.7 ± 5.6; not reported	14 Sub-elite male competitive distance runners; seasonal PB 5 km (min:s) rearfoot 16:43 ± 37, midfoot 16:09 ± 44; rearfoot 100.7 ± 18.8, midfoot 102.9 ± 18.0; no weekly training distance reported; midfoot runners had 7.6 years of experience, rearfoot runners had 6 years of experience; $\dot{V}{\text{O}}_{2\max }$ not reported; RE (in mLO_2_∙kg^−1^∙min^−1^) rearfoot: 49.8 ± 6.4 and midfoot: 48.4 ± 5.3	Portable gas analyzer (K4b^2^, Cosmed, Rome, Italy); 3.9 m∙s^−1^; 6 min; last min used for data analysis; visual inspection of steady-state in $\dot{V}{\text{O}}_2$^b^	mLO_2_∙kg^−1^∙min^−1^; n.a.; no subtraction reported	Concurrent; photoelectric cell system; outdoor track; shoes not reported; not reported	Contact time; rearfoot vs forefoot RE
Folland et al. [24]; correlational	50/47; males: 29 ± 7; 179 ± 6; 69 ± 6.3; not reported Females: 28 ± 7; 166 ± 7; 55.4 ± 6.5; not reported	97 Endurance runners of diverse competitive standards; seasonal PB for 10 km 37.58 ± 6.07 min for males, 43.31 ± 6.54 min for females; 69 ± 40 km∙week^−1^ for males, 51 ± 29 km∙week^−1^ for females; no running experience reported; $\dot{V}{\text{O}}_{2\max }$ (in mLO_2_∙kg^−1^∙min^−1^) 62.3 ± 7.1 for males, 55.5 ± 6.8 for females; RE not reported	Stationary gas analyzer (Vyntus CPX, Jaeger, San Diego, CA); 2.78 and 3.33 m∙s^−1^; 4 min; last min used for data analysis; lactate compared with lactate threshold, and RER < 1	Kcal∙kg^−1^∙km^−1^; Jeukendrup equation; standing EE subtracted from RE	Concurrent; 3D motion capture at 240 Hz; treadmill; standardized shoe; both legs	Vertical oscillation; braking; posture; stride parameters; lower limb angles
Gomez-Molina et al. [83]; groups differing in RE	21/0; 10 trained and 11 untrained; trained: 26.6 ± 6.6; 174.7 ± 4.9; 65.9 ± 4.2; not reported Untrained: 25.6 ± 4.8; 176.7 ± 5.3; 73.2 ± 6.3; reported	Trained and untrained male participants; best time in half marathon between 1:10:00 and 1:26:00 (trained participants); 57.5 ± 22 km∙week^−1^ (trained participants); ≥ 2 years of long distance running experience (trained participants); $\dot{V}{\text{O}}_{2\max }$ (in mLO_2_∙kg^−1^∙min^−1^) 61.8 ± 5.4 (trained participants) and 54.1 ± 5.8 (untrained participants); RE (in mLO_2_∙kg^−1^∙km^−1^) 207.6 ± 17.4 (trained participants) and 217.6 ± 13.9 (untrained participants)	Stationary gas analyzer (Ergocard, Medisoft Group, Sorinnes, Belgium); two groups: untrained (2.5 m∙s^−1^, 3.06 m∙s^−1^, 3.61 m∙s^−1^) and trained (3.06 m∙s^−1^, 3.61 m∙s^−1^, 4.17 m∙s^−1^); 5 min; last 3 min used for data analysis; RER < 1.0^b^	mLO_2_∙kg^−1^∙km^−1^; n.a.; no subtraction reported	Concurrent; contact laser platform; treadmill with 1% slope; standardized shoes; right side	Step frequency; step length; contact time; flight time; foot strike pattern
Gruber et al. [35]; groups differing in biomechanics	26/11; Rearfoot: 26.7 ± 6.1; 180 ± 10; 70.1 ± 10; not reported Forefoot: 25.6 ± 6.4; 180 ± 10; 68.7 ± 9.8; not reported	Experienced runners; no PBs reported; 46.2 ± 27.4 km∙week^−1^; preferred running speed of 3.7 ± 0.3 m∙s^−1^; no $\dot{V}{\text{O}}_{2\max }$ reported; no RE reported	Stationary gas analyzer (TrueOne 2400, Parvo Medics, Sandy, UT, USA); 3.00 3.5, 4 m∙s^−1^; 5 min; last 2 min used for data analysis; < 10% change in $\dot{V}{\text{O}}_2$ over 2-min period	mLO_2_∙kg^−1^∙min^−1^; n.a.; no subtraction reported	Concurrent; 3D motion capture at 240 Hz; treadmill; standardized shoe; right leg	Not reported
Hansen et al. [113]; correlational	12/0; 22.4 ± 3.1; 182 ± 6; 68.5 ± 7.7; not reported	International elite athletes from 800 m to 10 km; average PB 800 m: 1.53.69 min, average PB 1500 m: 3.54.77 min; no weekly training distance or running experience reported; $\dot{V}{\text{O}}_{2\max }$ (in mLO_2_∙kg^−1^∙min^−1^): 67.0 ± 4.2; RE (in mLO_2_∙kg^−1^∙min^−1^) 44.1 at 3.89 m∙s^−1^ and 58.7 at 5 m∙s^−1^	Stationary gas analyzer (MasterScreen CPX, CareFusion); 3.89 & 5 m∙s^−1^; 4 min; last minute used for data analysis; blood lactate^b^	mLO_2_∙kg^−1^∙min^−1^; n.a.; corrected for resting value	Concurrent; 3D motion capture for mechanical energy and video analysis for spatiotemporal parameters; treadmill; shoes not standardized; not reported	Step frequency; step length; contact time; swing time; body center of mass vertical oscillation; mechanical work; positive mechanical work; negative mechanical work; whole body stiffness normalized to body mass; leg stiffness
Heise et al. [114]; correlational	9/0; 33.6 ± 6.8; 175.2 ± 6.4; 68.2 ± 6.7; not reported	Well trained; no PBs, training experience, or weekly distance reported; $\dot{V}{\text{O}}_{2\max }$ (in mLO_2_∙kg^−1^∙min^−1^) 67.7 ± 6.2; RE (in mLO_2_∙kg^−1^∙min^−1^) 47.6 ± 2.8	Stationary gas analyzer (Amatek); 4.13 m∙s^−1^; 10 min; last 2 min used for data analysis; ‘no’ change in $\dot{V}{\text{O}}_2$ over 2-min period, < 83% $\dot{V}{\text{O}}_{2{\text{peak}}}$, and RER < 1.0	mLO_2_∙kg^−1^∙min^−1^; n.a.; no subtraction reported	Concurrent, bipolar electrodes at 600 Hz; treadmill; shoe standardization not reported; right side	Duration of activation relative to stance or swing and co-activation duration of rectus femoris, medial hamstrings, lateral hamstrings and gastric medialis
Heise and Martin [89]; correlational	16/0; 27.3 ± 4.8; 178.7 ± 7.4; 75 ± 8.3; not reported	Well-trained men; recent PB 10 km times between 38–45 min; no weekly training distance or running experience reported; $\dot{V}{\text{O}}_{2\max }$ (in mLO_2_∙kg^−1^∙min^−1^) 62.2 ± 3.0; RE (in mLO_2_∙kg^−1^∙min^−1^) 44.6 ± 3.3	Stationary gas analyzer (Vacumed, Ventura, CA, USA); 3.35 m∙s^−1^; 6 min; last 2 min used for data analysis; ‘no’ change in $\dot{V}{\text{O}}_2$ over 2-min period, < 83% $\dot{V}{\text{O}}_{2{\text{peak}}}$, and RER < 1.0	mLO_2_∙kg^−1^∙min^−1^; n.a.; no subtraction reported	Separate; force platform; treadmill and overground; standardized shoes; right side	Leg spring stiffness; vertical stiffness; mass-specific mechanical power output of the legs
Heise and Martin [88]; correlational	16/0; 27.3 ± 4.8; 178.7 ± 7.4; 75 ± 8.3; not reported	Recreational runners; recent PB 10 km times between 38^−^45 min; no weekly training distance or running experience reported; $\dot{V}{\text{O}}_{2\max }$ (in mLO_2_∙kg^−1^∙min^−1^) 62.2 ± 3.0; RE (in mLO_2_∙kg^−1^∙min^−1^) 44.6 ± 3.3	Stationary gas analyzer (Vacumed, Ventura, CA, USA); 3.35 m∙s^−1^; 6 min; last 2 min used for data analysis; ‘no’ change in $\dot{V}{\text{O}}_2$ over 2-min period, < 83% $\dot{V}{\text{O}}_{2{\text{peak}}}$, and RER < 1.0	mLO_2_∙kg^−1^∙min^−1^; n.a.; no subtraction reported	Separate; force platform; treadmill and overground; standardized shoes; right side	Total vertical impulse; net vertical impulse; anterior posterior impulse; medial–lateral impulse; contact time; max free moment; min free moment; net angular impulse
Heise et al. [90]; correlational	16/0; 27.3 ± 4.8; 178.7 ± 7.4; 75 ± 8.3; not reported	Well-trained men; recent PB 10 km times between 38–45 min; no weekly training distance or running experience reported; $\dot{V}{\text{O}}_{2\max }$ (in mLO_2_∙kg^−1^∙min^−1^) 62.2 ± 3.0; RE (in mLO_2_∙kg^−1^∙min^−1^) 44.6 ± 3.3	Stationary gas analyzer (Vacumed, Ventura, CA, USA); 3.35 m∙s^−1^; 6 min; last 2 min used for data analysis; ‘no’ change in $\dot{V}{\text{O}}_2$ over 2-min period, < 83% $\dot{V}{\text{O}}_{2{\text{peak}}}$, and RER < 1.0	W∙kg^−1^; Weir equation; no subtraction reported	Separate; force plate at 480 Hz and 2D video camera at 60 Hz to record the sagittal plane; treadmill; standardized shoes; not reported	Hip positive mechanical work; hip negative mechanical work; knee positive mechanical work; knee negative mechanical work; ankle positive mechanical work; ankle negative mechanical work; net total mechanical work
Hoogkamer et al. [41]; correlational	18/0; 23.7 ± 3.9; 177.8 ± 4.6; 64.3 ± 4.7; not reported	High-caliber runners; had recently run a sub-31-min 10 km at sea level, a sub-32-min 10-km race at local altitude or equivalent performance in a different distance running event; no weekly training distance or running experience reported; $\dot{V}{\text{O}}_{2\max }$ (in mLO_2_∙kg^−1^∙min^−1^) 72.1 ± 3.4 mLO_2_∙kg^−1^∙min^−1^; RE (in W∙kg^−1^) at 3.89 m∙s^−1^ 14.17 ± 0.82, 14.13 ± 0.84, 13.57 ± 0.76 for NS, AB, NP shoes, respectively. RE (in W∙kg^−1^) at 4.44 m∙s^−1^ 17.07 ± 1.02, 17.03 ± 1.02, 16.36 ± 0.99 for NS, AB, NP shoes, respectively. RE (in W∙kg^−1^) at 5 m∙s^−1^ 20.26 ± 1.06, 20.25 ± 1.18, 19.42 ± 1.08 for NS, AB, NP shoes, respectively	Stationary gas analyzer (TrueOne 2400, Parvo Medics, Salt Lake City, UT, USA); 3.89, 4.44, 5 m∙s^−1^; 5 min; last 2 min used for data analysis; post-exercise blood lactate values < 4 mmol∙L^−1^	W∙kg^−1^; Brockway equation; no subtraction reported	Concurrent; high- speed video at 240 Hz; treadmill; standardized shoes; both legs	GRF; step frequency; contact time
Howe et al. [110]; correlational	9/3; 34 ± 7; 173.7 ± 7.3; 68.4 ± 7.4; not reported	Endurance runners; no PBs reported; 74 ± 27 km∙week^−1^; 3 years running experience; $\dot{V}{\text{O}}_{2\max }$ (in mLO_2_∙kg^−1^∙min^−1^): 60.4 ± 5.8; RE not reported	Stationary gas analyzer (Oxycon Pro, Vyaire, UK); 2.22 m∙s^−1^; 3 min; last min used for data analysis; slope of regression line fitted through the $\dot{V}{\text{O}}_2$ did not significantly differ from zero	J∙kg^−1^∙m^−1^; Péronnet equation; no subtraction reported	Concurrent; tri-axial accelerometer; treadmill at 1% incline; shoes not reported; not reported	Stride length; stride frequency
Joubert et al. [42]; correlational	8/8; males: 29 ± 15; height not reported; 68.8 ± 10.9; not reported; females: 38 ± 7; height not reported; 58.5 ± 7.4; not reported	Trained runners that could run with blood lactate < 4 mmol∙L^−1^ at 3.33 m∙s^−1^; 5-km PB males 19.1 ± 2.6 min, females 20.3 ± 2.2 min; at least 3 runs per week in the prior 3 months; experience and $\dot{V}{\text{O}}_{2\max }$ not reported; RE (in mLO_2_∙kg^−1^∙km^−1^) at 2.78 m∙s^−1^ was 177.7 ± 11.3 for the Asics Hyper Speed shoes and 176.1 ± 11.1 for the Nike ZoomX Vaporfly Next% 2 shoes, and at 3.33 m∙s^−1^ 181.8 ± 8.4 for the Asics Hyper Speed shoes and 179.1 ± 8.3 for the Nike ZoomX Vaporfly Next% 2 shoes	Stationary gas analyzer (TrueOne 2400, Parvo, Medics, Sandy, UT, USA); 2.78 and 3.33 m∙s^−1^; 5 min; last 2 min used for data analysis; blood lactate < 4 mmol∙L^−1^	mLO_2_∙kg^−1^∙km^−1^; n.a.; no subtraction reported	Concurrent; heart rate monitor with accelerometer; treadmill; standardized shoes; not applicable	Step frequency; vertical oscillations; contact time
Joubert and Jones [43]; correlational	12/0; 26 ± 8; 181 ± 5; 68.0 ± 3.3; not reported	Trained distance runners; overall 5-km PB 16.0 ± 0.7 min, seasonal 5-km PB 16.4 ± 0.9 min; ≥ 3 weekly runs over last 3 months; not reported; $\dot{V}{\text{O}}_2$ (in mL O_2_∙kg^−1^∙min^−1^) at 4.44 m∙s^−1^ was 51.71 ± 2.02, 51.67 ± 2.07, 51.42 ± 1.72, 50.99 ± 1.83, 50.93 ± 1.82, 50.39 ± 1.71, 50.29 ± 1.72, 50.13 ± 1.86 for the Asics Hyperspeed, Hoka Rocket X, Brooks Hyperion Elite 2, New Balance RC Elite, Saucony Endorphin Pro, Asics Metaspeed Sky, Nike Vaporfly 2, Nike Alphafly shoes, respectively	Stationary gas analyzer (TrueOne 2400, Parvo, Medics, Sandy, UT, USA); 4.44 m∙s^−1^; 5 min; last 2 min used for data analysis blood lactate < 4 mmol∙L^−1^	W∙kg^−1^; Péronnet equation; no subtraction reported	Concurrent; heart rate monitor with accelerometer; treadmill; standardized shoes; not reported	Contact time imbalance; contact time; step frequency; vertical oscillation ratio; stride length
Kyröläinen et al. [84]; groups differing in RE	10/0; 5 power- and 5 endurance-trained athletes; power: 24 ± 5; 182 ± 6; 76.8 ± 6.4 Endurance: 26 ± 5; 178 ± 1; 69.9 ± 5; not reported	Power athletes were jumpers and sprinters, endurance athletes were runners and cross-country skiers; no PBs, weekly training distance or experience reported; $\dot{V}{\text{O}}_{2\max }$ (in mL O_2_∙kg^−1^∙min^−1^) was 72.7 ± 3.7 and 54.1 ± 4.4 for endurance and power athletes, respectively; RE not reported	Stationary gas analyzer (Douglas bag); 2.50, 3.25, and 4 m∙s^−1^; 5 min; last 1–2 min used for data analysis; visual inspection of respiratory gas exchange data for steady state	J∙kg^−1^; assumed 1 mL O_2_ consumed yielded 20.18 J at RER of 0.82, with 0.01 unit change in RER corresponding to change of 42 J; no subtraction reported	Concurrent; electro goniometer for knee and ankle angles, kinematic arm for external work; treadmill and track; shoes not reported; not reported	Mechanical efficiency; maximal knee extensor force; maximal plantar flexors force; maximal velocity; step frequency; contact time; angular velocity of knee joint in braking phase; angular velocity of ankle joint in braking phase; vertical displacement of center of mass; average net F2 (an undefined outcome)
Kyröläinen et al. [111]; correlational	9/8; males: 20 ± 2; 180 ± 3; 68.1 ± 1.9; not reported Females: 21 ± 3; 168 ± 2; 55.7 ± 4.5; not reported	Middle-distance runners; no PBs reported; training distance during year preceding study 72.5 ± 32.5 km∙week^−1^; running experience 7 ± 3 years; $\dot{V}{\text{O}}_{2\max }$ and RE not reported	Stationary gas analyzer (SensorMedics V_max_ 229, Yorba, Linda, CA); 5 m∙s^−1^; 3 min; last 20 s used for data analysis; visual inspection for steady state in respiratory gas data	mLO_2_∙kg^−1^∙min^−1^; n.a.; no subtraction reported	Concurrent; force plate at 150 Hz and high-speed 2D video analysis for kinematics at 200 Hz; indoor track; shoes not reported; right leg	Biceps femoris braking EMG; Biceps femoris push-off EMG; Gastrocnemius push off EMG Note that more speeds and variables were measured, but no correlations were reported for these
Lemire et al. [40]; correlational	19/10; 34 ± 10; 174 ± 9; 68.3 ± 12.2; not reported	Description not reported; no PBs reported; running 1–5 ×/week; experience not reported; $\dot{V}{\text{O}}_{2\max }$ (in mLO_2_∙kg^−1^∙min^−1^) 56.6 ± 8.9; RE not reported	Stationary gas analyzer (Quark, Cosmed, Rome, Italy); 2.22, 2.78, 3.33, and 3.89 m∙s^−1^; 4 min; data from 3:15–3:45 used for data analysis; RER < 1.0 and blood lactate < 4 mmol∙L^−1^, with $\dot{V}{\text{O}}_2$ correction applied when these conditions were not met	mLO_2_∙kg^−1^∙min^−1^; n.a.; no subtraction reported	Concurrent; instrumented treadmill at 1000 Hz; treadmill; shoes not reported; not reported	Step length; step frequency; contact time; aerial time; max vertical GRF; vertical displacement of the CoM during ground contact; duty cycle; vertical stiffness
Li et al. [106]; correlational	28/0; 20.7 ± 1.2; 177.3 ± 4.9; 60.8 ± 5.2; not reported	Collegiate long-distance runners; no PBs or weekly running distance reported; 4-y running experience; $\dot{V}{\text{O}}_{2\max }$ (in mLO_2_∙kg^−1^∙min^−1^) 65.78 ± 4.99; RE (in mLO_2_∙kg^−1^∙min^−1^) 47.13 ± 3.11, 53.96 ± 2.86, 60.46 ± 0.04, at 3.33, 3.89, and 4.44 m∙s^−1^, respectively	Portable gas analyzer (K5, Cosmed, Rome, Italy); 3.33, 3.89 and 4.44 m∙s^−1^; 4 min; last min used for data analysis; $\dot{V}{\text{O}}_2$, heart rate, and blood lactate checked for steady state	mLO_2_∙kg^−1^∙min^−1^; n.a.; no subtraction reported	Separate; 3D motion capture at 120 Hz. Two force plates at 1000 Hz; biomechanics assessed overground and treadmill for RE; shoes not reported; not reported	Leg stiffness
Lussiana et al. [86]; correlational and groups differing in biomechanics	58/0; 30 ± 8; 177 ± 5; 72 ± 9; not reported	Recreational runners able to run at 3.33 m∙s^−1^ with submaximal effort; no PBs, weekly running distance, experience reported or $\dot{V}{\text{O}}_{2\max }$ reported; RE (in mLO_2_∙kg^−1^∙m^−1^) 6.00 and 5.94 for aerial and terrestrial runners, respectively. RE (in kcal∙kg^−1^∙km^−1^) 0.83 and 0.83 for aerial and terrestrial runners, respectively	Portable gas analyzer (Metamax 3B, Cortex Biophysik, Leipzig, Germany); 3.33 m∙s^−1^; 5 min; last min used for data analysis; visual inspection of $\dot{V}{\text{O}}_2$ and $\dot{V}{\text{CO}}_2$ and RER < 1.0	mLO_2_∙kg^−1^∙m^−1^ and kcal∙kg^−1^∙km^−1^; Lusk equation; corrected for standing $\dot{V}{O}_{2}$	Concurrent; optoelectronic system at 1000 Hz and high-speed 2D camera at 200 Hz; treadmill; shoes not standardized; not reported	Vscore (a subjective assessment of running technique); leg stiffness; joint angles; contact time; flight time; step frequency; center of mass displacement; muscle activation outcomes; knee, ankle and foot ground angles
Lussiana et al. [39]; correlational	33/21; males: 31 ± 8; 175 ± 6; 66 ± 9; not reported Females: 32 ± 7; 162 ± 3; 52 ± 4; not reported	Trained runners; < 50 min (10 km), < 1 h 50 min (21 km), < 3 h 50 min (42 km); males: 53 ± 15 km∙week^−1^, females: 50 ± 14; no experience $\dot{V}{O}_{2}$ max or RE reported	Stationary gas analyzer (TrueOne 2400, Parvo Medics, Sandy, UT, USA); 3.33, 3.89, 4.44 m∙s^−1^; 4 min; last min used for data analysis; visual inspection of $\dot{V}{\text{O}}_2$ and $\dot{V}{\text{CO}}_2$ and RER < 1.0	Kcal∙kg^−1^∙km^−1^; Lusk equation; no subtraction reported	Separate; 3D motion capture at 200 Hz; treadmill; shoes not standardized; not reported	Vertical displacement center of mass during a step; contact time; flight time; step frequency; step symmetry; duty factor
Man et al. [97]; correlational	9/0; 22 ± 3; 178 ± 5; 70 ± 8; not reported	Experienced runners; no PBs reported; weekly running distance 50 km∙week^−1^; no experience or $\dot{V}{\text{O}}_{2\max }$ reported; RE (in mLO_2_∙kg^−0.75^∙min^−1^) 115.42	Portable gas exchange (Metamax 3B Cortex, Leipzig, Germany); 2.78 m∙s^−1^; 12 min; data from 3–12 min used for data analysis; RER < 1.15	mLO_2_∙kg^−0.75^∙min^−1^; n.a.; no subtraction reported	Concurrent; plantar pressure sensors system at 100 Hz; treadmill; standardized shoes; right side	Vertical stiffness; leg stiffness; stride frequency; contact time; stride length
Martin et al. [115]; correlational	16/0; 27.3 ± 5; 178.7 ± 7.4; 76.1 ± 7.8; not reported	Recreational male runners; no PBs, weekly distance, experience or $\dot{V}{\text{O}}_{2\max }$ reported; RE (in mLO_2_∙kg^−1^∙min^−1^) 44.6	Stationary gas analyzer (Beckman OM-11, Germany); 3.35 m∙s^−1^; 6 min; last 2 min used for data analysis; increase in $\dot{V}{\text{CO}}_2$ < 1 mLO_2_∙kg^−1^∙min^−1^ and RER < 1.0	mLO_2_∙kg^−1^∙min^−1^; n.a.; no subtraction reported	Separate; cine camera (60 Hz); treadmill; standardized shoes; both legs	No energy transfer; complete energy transfer within and between all segments; exchange between potential and kinetic energy components; running net joint moment power; rate of mechanical energy transfer between adjacent segments; total body angular impulse
Moore et al. [98]; correlational	0/11; 21.8 ± 2.9; 164.8 ± 4.2; 60.4 ± 6.6; not reported	Recreational runners; no PBs or weekly distance reported; ≤ 2 years of running experience; no $\dot{V}{\text{O}}_{2\max }$ reported; no RE reported	Stationary gas analyzer (Metalyser II Cortex, Biophysik, Leipzig, Germany); 2.53, 3.06, 3.33 m∙s^−1^; 6 min; last 2 min used for data analysis; visual inspection of $\dot{V}{\text{O}}_2$ and RER < 1.0^b^	mLO_2_∙kg^−1^∙km^−1^; n.a.; no subtraction reported	Concurrent; surface EMG; treadmill; no standardized shoes; not reported	Rectus femoris biceps femoris % stance; vastus lateralis biceps femoris % stance; rectus femoris gastrocnemius lateralis % stance
Muniz-Pardos et al. [116]; correlational	4/0; 27.3 ± 5.1; not reported; 52.3 ± 3.4; Kenyan	Elite Kenyan long distance runners; no PBs, weekly running distance, or experience reported; $\dot{V}{\text{O}}_{2\max }$ (in mLO_2_∙kg^−1^∙min^−1^) 78.5 ± 6.1; RE (in mLO_2_∙kg^−1^∙min^−1^) 69.4 ± 4.7	Not reported; 4.44 m∙s^−1^; 3 min; unknown what duration was used for data analysis; not reported	mLO_2_∙kg^−1^∙min^−1^; n.a.; no subtraction reported	Not reported; wireless foot-worn inertial sensors; treadmill at 1° grade; shoes not reported; not reported	Stride frequency; contact time; flight time; pitch; eversion
Nummela et al. [117]; correlational	28/0; 19.8 ± 1.1; 182 ± 7; 69.4 ± 7.5; no ethnicity reported	10 Distance runners, 8 orienteers and 7 triathletes (all endurance athletes belonging to the junior national team); no PBs, weekly training distance, or running experience reported; $\dot{V}{\text{O}}_{2\max }$ (in mLO_2_∙kg^−1^∙min^−1^) 63.9 ± 5.7; RE (in mLO_2_∙kg^−1^∙min^−1^) 49.9 ± 3.3 at 3.89 m∙s^−1^, RE (in mLO_2_∙kg^−0.75^∙min^−1^) 144 ± 10 at 3.89 m∙s^−1^	Portable gas analyzer (Oxycon Mobile, Viasys Healthcare GmbH, Hoechberg, Germany); 3.89 m∙s^−1^; 4 min, unknown duration used for data analysis) not reported, but mentioned they verified steady state	mLO_2_∙kg^−0.75^∙min^−1^; n.a.; no subtraction reported	Concurrent; photocell gates and 2D and 3D force platforms 170 and 400 Hz; 200-m indoor track; not reported; not reported	Stride frequency; stride length; vertical effective force; mass-specific horizontal force; ground contact force
Ogueta-Alday et al. [32]; groups differing in RE	20/0; rearfoot strikers (*n* = 10): 26.2 ± 6.5; 180 ± 6; 68.1 ± 4.7; not reported Midfoot strikers (*n* = 10): 28.7 ± 6.6; 177 ± 4; 66.1 ± 5.7; not reported	Well-trained, long-distance runners; recent half-marathon performance between 1:05:00 and 1:15:00; training volume during the month preceding the study was 91 ± 24 km∙week^−1^; running experience 12 ± 6 years; $\dot{V}{\text{O}}_{2\max }$ (in mLO_2_∙kg^−1^∙min^−1^) rearfoot 65.8 ± 4.6, midfoot 66.7 ± 3.6; RE (in mLO_2_∙kg^−1^∙km^−1^) was 204.1 ± 9.1, 199.8 ± 14.5 and 205.5 ± 14.6 for the rearfoot group at speeds 3.06 m∙s^−1^, 3.61 m∙s^−1^ and 4.17 m∙s^−1^, respectively. RE (in mLO_2_∙kg^−1^∙km^−1^) was 215.7 ± 13.6, 220.4 ± 11.1, 216.3 ± 10.0 for the midfoot group at speeds 3.06 m∙s^−1^, 3.61 m∙s^−1^ and 4.17 m∙s^−1^, respectively	Stationary gas analyzer (Ergocard; Medisoft Group, Sorinnes, Belgium); 3.06, 3.61, 4.17 m∙s^−1^; 6 min; last 3 min used for data analysis; RER < 1.0^b^	mLO_2_∙kg^−1^∙min^−1^ and mLO_2_∙kg^−1^∙km^−1^; n.a.; no subtraction reported	Concurrent; contact laser platform; treadmill; shoes not standardized; right side	Step length; step frequency; foot strike pattern; contact time; step length; flight time
Pastor et al. [118]; correlational	17/0; road runners (*n* = 7) 27 ± 4.8; 177.1 ± 6.7; 62.6 ± 3.9; not reported Trail runners (*n* = 10) 30.8 ± 8.3; 176.7 ± 6.7; 65.6 ± 5.6; not reported	Members of national team; road PB 10 km 29:17 average with times ranging from 28:06 to 30:56 for road runners, trial runners performance index of 864 points; no weekly training distance, running experience, or $\dot{V}{\text{O}}_{2\max }$ reported; RE (in J∙kg^−1^∙m^−1^) was 4.37 ± 0.27 for trail runners and 4.09 ± 0.31 for road runners at 2.78 m∙s^−1^, and RE was 4.32 ± 0.22 for trail runners and 4.06 ± 0.29 for road runners at 3.89 m∙s^−1^	Portable gas analyzer (Metamax 3B, Cortex, Leipzig, Germany); 2.78 and 3.89 m∙s^−1^; 4 min; last min used for data analysis; increase in $\dot{V}{\text{O}}_2$ < 100 mLO_2_∙min^−1^ and RER < 1.0^b^	J∙kg^−1^∙m^−1^; Péronnet equation; no subtraction reported	Concurrent; optoelectronic system; treadmill; not reported; not reported	Flight time; contact time; step frequency; duty factor; leg stiffness; vertical stiffness
Patoz et al. [99]; correlational	31/21; males: 31 ± 8; 174 ± 7; 66 ± 10; not reported Females: 32 ± 9; 162 ± 4; 52 ± 5; not reported	Trained runners; recent half-marathon times: males 91 ± 9 min, females: 102 ± 9 min; weekly training distance: males 55 ± 19 km, females 50 ± 21 km; Experience: males 8 ± 6 years, females 7 ± 4 years; $\dot{V}{\text{O}}_{2\max }$ and RE not reported	Stationary gas analyzer (TrueOne 2400, Parvo Medics Inc., Sandy, UT, USA); 2.78, 3.33, and 3.89 m∙s^−1^; 4 min; last min used for data analysis; visual inspection of $\dot{V}{\text{O}}_2$ and $\dot{V}{\text{CO}}_2$ and RER < 1.0	mLO_2_∙kg^−0.75^∙km^−1^; n.a.; no subtraction reported	Separate; 3D motion capture; treadmill; shoes not standardized; not reported	Duty factor; V^a^ score (a subjective assessment); vertical displacement of center of mass; range of horizontal displacement of elbow; vertical displacement of the pelvis center of mass at foot strike; horizontal distance between the heel marker and pelvis center of mass at foot strike; foot strike angle at ground contact
Rogers et al. [119]; correlational	11/0; 20 ± 2.9; 181.7 ± 5.3; 68.2 ± 6.7; not reported	Highly trained runners with IAAF scores > 750 points in events 800–5000 m (average IAAF score 850 ± 90); PB 1500 m 4:02 ± 0:06; weekly training volume of 90.8 ± 15.6 km; no running experience reported; $\dot{V}{\text{O}}_{2\max }$ (in mLO_2_∙kg^−1^∙min^−1^) 67.6 ± 3.8; RE (in kcal∙kg^−1^∙km^−1^) 1.00 ± 0.03	Stationary gas analyzer (TrueOne 2400, Parvo Medics, Sandy, UT, USA); 3.33, 3.89, 4.44, 5 m∙s^−1^; 4 min; last min used for data analysis; not reported	kcal∙kg^−1^∙km^−1^; equation not reported; no subtraction reported	Concurrent; 2D video camera at 210 Hz; treadmill at 1% incline; shoes not reported; not reported	Leg stiffness; contact time; flight time
Santos-Concejero et al. [85]; groups differing in RE	17/0; divided Eritrean and European: 23.3 ± 4.8 and 28.0 ± 4.2; 172 ± 5.2 and 173.6 ± 5.1; 57.8 ± 3.3 and 63.5 ± 7.5; Eritrean and European	Elite long distance runners (Eritrean and European); 10-km time: 27.7 ± 0.8 min and 28.5 ± 0.8 min; no weekly running distance or experience reported; $\dot{V}{\text{O}}_{2\max }$: 73.5 ± 6.0 mLO_2_∙kg^−1^∙min^−1^ and 77.2 ± 5.2 mLO_2_∙kg^−1^∙min^−1^; RE (in mLO_2_∙kg^−1^∙km^−1^) 191.4 ± 10.4 and 205.9 ± 13.3 at 5.28 m∙s^−1^	Stationary gas analyzer (Vmax 29C, SensorMedics Corp, Yorba Linda, CA, USA); 4.72 and 5.28 m∙s^−1^, (5.83 m∙s^−1^ excluded); 6 min; last 3 min used for data analysis; increase in $\dot{V}{\text{O}}_2$ of < 1.5 mLO_2_∙kg^−1^∙km^−1^) during last 2 min^b^	mLO_2_∙kg^−1^∙km^−1^; n.a.; no subtraction reported	Concurrent; optical measurement system; treadmill; shoes not standardized; not reported	Contact time; swing time; stride length; stride frequency
Santos-Concejero et al. [92]; correlational	15/0; 23.7 ± 4.2; 170.5 ± 6.3; 54.8 ± 6.3; Kenyan	Kenyan elite runners; 10-km PB 28.7 ± 0.4 min, half-marathon PB 62.2 ± 1.0 min; weekly training volume 128.9 ± 18.8 km∙week^−1^; no experience reported; RE 0.94 ± 0.07 (3.33 m∙s^−1^) and 0.93 ± 0.07 kcal∙kg^−1^∙km^−1^ (5.56 m∙s^−1^), oxygen cost of running 192.2 ± 14.7 (2.78 m∙s^−1^) and 184.8 ± 9.9 (5.56 m∙s^−1^) mLO2∙kg^−1^∙km^−1^	Automated breath-by-breath system (COSMED Quark CPET, Rome, Italy); 3.33 and 5.56 m∙s^−1^; 6 min; last min used for data analysis; below each athlete’s lactate threshold and RER < 1.0	RE in kcal∙kg^−1^∙km^−1^, and mLO2∙kg^−1^∙km^−1^; Lusk equation; no subtraction reported	Separate; 3D motion capture at 250 Hz, GRF data collected with force platform at 2000 Hz; RE on treadmill, biomechanical data on 40-m indoor synthetic track; shoes not reported; leg not reported	Contact time; swing time; stride length; stride frequency; vertical GRF; vertical initial loading rate; peak braking force
Seki et al. [112]; correlational	12/0; 21.9 ± 0.8; 171 ± 5; 60.1 ± 4.2; Japanese	Middle- and long-distance runners; no PBs, weekly running distance, or running experience reported; > 60 mL∙kg^−1^∙min^−1^; RE (in J∙kg^−1^∙m^−1^) 4.15 ± 0.57 at 3.75 m∙s^−1^	Stationary gas analyzer (AE-301s; Minato, Medical Science, Japan); 3.75 m∙s^−1^; 3 min; last min used for data analysis; visual inspection of $\dot{V}{\text{O}}_2$^b^	J∙kg^−1^∙m^−1^; Kyröläinen method to determine EE, then divide EE by speed to obtain RE; no subtraction reported	Concurrent; 3D motion capture and sEMG; treadmill; standardized shoes; right leg	Positive mechanical work; negative mechanical work; total mechanical work; step length; step frequency; contact time; vertical displacement to cover 1 m; maximal ankle, knee, and hip angle, velocity and range of motion
Seminati et al. [91]; correlational	19/0; untrained runners: 33.1 ± 13.2; 175.9 ± 4.7; 70.6 ± 3.4; not reported Occasional runners: 31.9 ± 11.8; 177.3 ± 4.0; 67.3 ± 6.1; not reported Skilled runners: 42.6 ± 7.4; 177.8 ± 4.4; 68.2 ± 4.9; not reported	Untrained, occasional and skilled runners (UR, OR, and SR, respectively); SR marathon PB 2:44:24 ± 10 min 12 s; UR < 2 h∙week^−1^, OR 2–6 h∙week^−1^, SR > 6 h∙week^−1^; no running experience, $\dot{V}{\text{O}}_{2\max }$ or RE reported	Portable gas analyzer (K4b^2^, Cosmed Rome, Italy); fixed speeds of 2.22–4.44 m∙s^−1^ with increments of 0.56 m∙s^−1^; 5 min; last 2 min used for data analysis; RER < 1	J∙kg^−1^∙m^−1^; assumed 1 mL O_2_ consumed yielded 20.9 J; resting $\dot{V}{\text{O}}_2$ subtracted	Not reported; 3D motion capture; treadmill; shoes not standardized; not reported	Global symmetry index; symmetry index in vertical direction; symmetry index in anterior–posterior direction; symmetry index in medial–lateral direction
Sinclair et al. [107]; correlational	12/0; 23.7 ± 2.3; 176.5 ± 5.8; 75.6 ± 7.6; not reported	Experienced runners; PBs not reported; minimum of 3 × /week and 35 km∙week^−1^; running experience and $\dot{V}{\text{O}}_{2\max }$ not reported; RE: Shoe A = 42.72 ± 2.17 and Shoe B = 42.75 ± 1.95 mLO_2_∙kg∙min^−1^	Stationary gas analyzer (MetaLyzer 3B, Cortex Biophysic, Leipzig, Germany); 4 m∙s^−1^; 6 min; unknown duration for data analysis; post-exercise blood lactate of < 2.0 mmol∙L^−1^	mLO_2_∙kg^−1^∙min^−1^; n.a.; no subtraction reported	Separate; 3D motion capture at 250 Hz; treadmill; standardized shoes; right side	Peak stance phase hip flexion; peak sagittal plane ankle velocity; peak sagittal plane knee excursion; stance phase activation of the vastus medialis
Storen et al. [120]; correlational	11/0; 21.4 ± 3.9; 181.1 ± 3.3; 71.5 ± 6.1; not reported	Elite endurance athletes competing in orienteering, cross-country skiing, biathlon and long-distance running; PB 3 km 566.9 ± 42.6 s; weekly training distance or running experience not reported; $\dot{V}{\text{O}}_{2\max }$ (in mLO_2_∙kg^−1^∙min^−1^) 75.8 ± 6.2; RE not reported	Portable gas analyzer (Metamax II, Cortex, Leipzig, Germany); 4.17 m∙s^−1^; 5 min; unknown duration for data analysis; speeds were < 85% of $\dot{V}{\text{O}}_{2\max }$	mLO_2_∙kg^−0.75^∙m^−1^; n.a.; no subtraction reported	Separate; pressure insoles and force platform; treadmill; not reported; not reported	Average vertical peak eccentric and concentric force; average horizontal peak eccentric and concentric force; average peak force total; contact time; step frequency; step length; time to peak force
Tam et al. [101]; correlational	14/0; 24.2 ± 4.2; 170.5 ± 6.3; 54.8 ± 6.3; Kenyan	Elite runners; 10-km race time 28.7 min ± 0.4 min, half-marathon time 62.2 ± 1.0 min; 128.9 ± 18.8 km∙week^−1^; running experience not reported; $\dot{V}{\text{O}}_{2\max }$ (in mLO_2_∙kg^−1^∙min^−1^) 71.9 ± 5.1; RE (in kcal∙kg^−1^∙km^−1^) 0.94 ± 0.18 over a 12-km run and 0.93 ± 0.08 over a 20-km run	Stationary gas analyzer (Quark, Cosmed, Rome, Italy); 3.33 m∙s^−1^ and 5.56 m∙s^−1^; 6 min; last 0.5 min used for data analysis; speeds were slower than lactate threshold of each athlete, and RER < 1.0	kcal∙kg^−1^∙km^−1^; Lusk equation; no subtraction performed	Separate; 3D motion capture at 250 Hz and force platform at 2000 Hz; 60-m indoor synthetic track, RE determined on treadmill with 1% incline; not reported; right side	Rectus femoris–biceps femoris co-activation; rectus femoris–gluteus medius co-activation
Tam et al. [102]; correlational	30/0; 25.8 ± 5; 175.2 ± 7.5; 66 ± 13.3; not reported	Trained runners; PB 10 km 36.03 ± 7.46; weekly training distance, running experience and $\dot{V}{\text{O}}_{2\max }$ not reported; RE (in mLO_2_∙kg^−1^∙km^−1^) 206.03 ± 19.02	Stationary gas analyzer (Quark, Cosmed Rome, Italy); 3.3 m∙s^−1^; 6 min; last 0.5 min used for data analysis; speeds were slower than lactate threshold of each athlete, RER < 1.0	mLO_2_∙kg^−1^∙km^−1^; n.a.; no subtraction reported	Separate; 3D motion capture at 250 Hz and force platform at 2000 Hz; treadmill at 1% incline; biomechanical data on a 60-m indoor synthetic running track; no standardized shoes; EMG was only measured for the right limb, other data both limbs	Ground contact time; stride frequency; stride duration; swing time; stride length; ankle stiffness; knee stiffness; lateral gastrocnemius and tibialis anterior co-activation; medial gastrocnemius; lateral gastrocnemius; tibialis anterior; peroneus longus; biceps femoris; rectus femoris; gluteus medius
Tanji et al. [108]; correlational	11/0; 22.4 ± 3.1; 182 ± 6; 68.5 ± 7.7; not reported	Highly trained long-distance runners; IAAF scores: 1038 ± 48; PBs, weekly training distance, running experience, and $\dot{V}{\text{O}}_{2\max }$ unknown; RE at 4.5 m∙s^−1^: 0.90 ± 0.08 kcal∙kg^−1^∙km^−1^, RE at 6 m∙s^−1^: 1.00 ± 0.07 kcal∙kg^−1^∙km^−1^	Not reported; speed: 4.5 m∙s^−1^ (6 m∙s^−1^ excluded); 3 min; unknown duration for data analysis; below individual lactate threshold	Kcal∙kg^−1^∙km^−1^; no equation reported; no subtraction reported	Not reported	Ground contact time
Tartaruga et al. [103]; correlational	0/9; 26 ± 14.6; 160.8 ± 6.2; 50.2 ± 8.0; not reported	More than 5 years experience; no PBs or weekly training distance reported; > 5 years experience; $\dot{V}{\text{O}}_{2\max }$ (in mLO_2_∙kg^−1^∙min^−1^) 43.13 ± 4.08; no RE reported	Not reported; 6-min stages at 3.33 m∙s^−1^ (85% $\dot{V}{O}_{2}$ max); 6 min; last 2 min used for data analysis; RER < 0.95	mLO_2_∙kg^−1^∙min^−1^; n.a.; no subtraction reported	Concurrent; 2D video camera at 120 Hz; treadmill; not standardized; left side	Stride time; stride length; stride frequency; balance time; relative stride length; contact time
Tartaruga et al. [124]; correlational	16/0; 27 ± 5.7; 174 ± 8; 64.5 ± 5.8; not reported	Long-distance runners; 10-km times between 30–36 min; 66.8 ± 13.6 km∙week^−1^; 7.7 ± 3.2 years running experience; $\dot{V}{\text{O}}_{2\max }$ (in mLO_2_∙kg^−1^∙min^−1^) 56.36 ± 4.7 mLO_2_∙kg^−1^∙min^−1^; RE (in mLO_2_∙kg^−1^∙min^−1^) 44.85 ± 4.7	Portable gas analyzer (AEROSPORT-KB1-C, Ann Arbor, MI); 4.4 m∙s^−1^; 6 min; last 2 min used for data analysis; 89% of the average velocity at the ventilatory threshold	mLO_2_∙kg^−1^∙min^−1^; n.a.; no subtraction reported	Concurrent; 2D cameras (sagittal and frontal plane) at 120 Hz; treadmill at 1°; shoes not standardized; left side	Stride time; contact time; balance time; stride length; relative stride length; stride frequency; vertical oscillation center of mass; range of elbow motion; internal knee and ankle angles at foot strike and toe-off and max during stance; max trunk flexion during stance; max pronation of subtalar joint; external mechanical work
Vercruyssen et al. [87]; correlational and groups differing in biomechanics	13/0; 38.2 ± 4.8; 175.5 ± 4.9; 68.2 ± 6.0; not reported	Well-trained competitive male runners; no PBs reported; 70 ± 10 km∙week^−1^; trail running experience 6.4 ± 2.4 years; $\dot{V}{\text{O}}_{2\max }$ (in mLO_2_∙kg^−1^∙min^−1^) 62.5 ± 3.5; no RE reported	Stationary gas analyzer (Oxycon Alpha, Jaeger, Germany); 5 min; last 2 min used for data analysis; 2.77 m∙s^−1^; not reported, but did mention they verified a steady state	mLO_2_∙kg^−1^∙min^−1^; n.a.; no subtraction reported	Concurrent; high-speed 2D camera at 240 Hz; treadmill with 1% slope; standardized shoes; not reported	Vertical stiffness; foot strike angle
Williams and Cavanagh [23]; groups differing in RE	31/0; no anthropometrics reported	Could run at a speed of 3.57 m∙s^−1^ for 4 min with post-exercise lactate of < 2 mmol∙L^−1^; no PBs reported; weekly running distance of ≥ 40 km∙week^−1^; running experience, $\dot{V}{\text{O}}_{2\max }$ and RE not reported	Stationary gas analyzer (Beckman, Germany); 3.57 m∙s^−1^; 8 min; last 2 min used for data analysis; post-exercise lactate of < 2 mmol∙L^−1^	mLO_2_∙kg^−1^∙min^−1^; n.a.; resting oxygen cost subtracted	Separate; 2D video analysis; not reported; not reported	Step frequency; vertical displacement of pelvis during stance phase
Willis et al. [93]; correlational	6/5; 33.6 ± 4.3; 170 ± 5; 62.4 ± 7.3; not reported	Top-level ultradistance trail runners, group included 3 men in the top 30 and 5 women in the top 20 of the performance index ranking of the international trail running association; no PBs reported; no weekly training distance, running experience, $\dot{V}{\text{O}}_{2\max }$, or RE not reported	Portable gas analyzer (K5; Cosmed, Rome, Italy); men 4.17 m∙s^−1^, women 3.61 m∙s^−1^; 5 min; last 2 min used for data analysis; RER < 1.0	mLO_2_∙kg^−1^∙min^−1^; mLO_2_∙kg^−1^∙km^−1^; J∙kg^−1^∙m^−1^; undefined energy equivalent of oxygen; no subtraction reported	Not reported; inertial measurement unit sensors at 256 Hz treadmill; not reported; both sides	Vertical stiffness; contact time; aerial time; stride frequency
Zhang et al. [104]; correlational	30/0; 21 ± 1; 180 ± 6; 72.1 ± 9.3; not reported	Recreational-trained runners; PBs not reported; 20–30 km∙week^−1^ for 3 months prior to the study; ≥ 2 years running experience; $\dot{V}{\text{O}}_{2\max }$ (in mLO_2_∙kg^−1^∙min^−1^) 54.02 ± 4.67; RE (in mLO_2_∙kg^−1^∙min^−1^) 40.60 ± 3.03 at 2.78 m∙s^−1^	Portable metabolic analyzer (K5, Cosmed, Italy); 2.78 m∙s^−1^; 4 min; last min used for data analysis; RER < 1.0	mLO_2_∙kg^−1^∙min^−1^; n.a.; no subtraction reported	Separate; 3D motion capture at 250 Hz and instrumented treadmill at 1000 Hz; treadmill; standardized shoes; both sides	Vertical stiffness; leg stiffness; knee stiffness; ankle stiffness

*AB* Adidas adizero Adios BOOST 2, *CoM* center of mass, *2D* two dimensional, *3D* three dimensional, *EE* energy expenditure, *EMG* electromyography, *GRF* ground reaction force, *IAAF* International Association of Athletics Federations, *IC* initial contact, *ITRA* International Trail Running Association, *n.a.* not applicable, *NP* Nike prototype Zoom Vaporfly, *NS* Nike Zoom Streak 6, *OBLA* onset of blood lactate accumulation, *OR* occasional runners, *PB* personal best, *RE* running economy, *RER* respiratory exchange ratio, *SR* skilled runners, *UR* untrained runners, $\dot{V}{\text{CO}}_2$ rate of carbon dioxide output; $\dot{V}{\text{O}}_2$ rate of oxygen consumption, $\dot{V}{\text{O}}_{2\max }$ maximal rate of oxygen consumption, $\dot{V}{\text{O}}_{2{\text{peak}}}$ peak rate of oxygen consumption

^a^*n* was initially 35 (21 males) but 5 were excluded due to blood lactate values > 3 mM. As the sex of the excluded participants was not reported, we assumed that 2 males and 3 females were excluded

^b^Information provided by authors via personal contact

Twenty-seven studies assessed RE during one fixed speed, and 24 studies assessed RE during two or more speeds. In one study [93], male and female participants ran at a different constant speed. RE was expressed in mlO_2_∙kg^−1^∙km^−1^ in nine studies, mlO_2_∙kg^−1^∙min^−1^ in 23 studies, and kcal∙kg^−1^∙km^−1^ in seven studies. Other commonly employed units for RE were W∙kg^−1^ (*k* = 4), J∙kg^−1^ (*k* = 1), J∙kg^−1^∙m^−1^ (*k* = 5), J∙kg^−1^∙min^−1^ (*k* = 2), mLO_2_∙kg^−0.75^∙km^−1^ (*k* = 1), and mlO_2_∙kg^−0.75^∙min^−1^ (*k* = 2). Several studies reported RE in multiple units, and thus the total is higher than 51. Forty studies described methods used to check for a steady state, which included verification of the respiratory exchange ratio below 1.0 (22 studies [24, 39, 40, 80, 81, 86, 88–90, 93–104], measurement of blood lactate concentration (12 studies [23, 24, 40–43, 82, 92, 105–108]), and visual inspection of a plateau in the oxygen consumption ($\dot{V}{\text{O}}_2$) and/or carbon dioxide expired $(\dot{V}{\text{CO}}_2 )$ data (21 studies [34, 35, 39, 80, 82, 84–86, 88–90, 94–96, 98, 99, 106, 109–112]). Most studies (*k* = 18) employed multiple methods to check for steady state [32, 83, 87, 113–120], while 11 studies did not report any steady-state verification. Six studies subtracted resting (3 studies [23, 91, 111, 113]) or standing (3 studies [24, 81, 86]) $\dot{V}{\text{O}}_2$ or energy expenditure from running $\dot{V}{\text{O}}_2$ or energy expenditure. Most studies (*k* = 44) did not report whether such a subtraction was performed, and one study [101] explicitly stated that no subtraction was done. Four studies used allometric scaling when normalizing RE for body mass [97, 99, 117, 120] while 47 studies used linear scaling. Finally, six studies used the Péronnet equations to compute the energetic cost from $\dot{V}{\text{O}}_2$ and $\dot{V}{\text{CO}}_2$, one study used the Brockway equation, three referred to Fletcher [121], who used the Lusk equation, one study used the Jeukendrup equation [122], one study used a method described by Kyröläinen based on blood lactate [111], and one used the Weir equation [123].

Thirty-one studies assessed running biomechanics and RE simultaneously, while 14 studies performed these assessments separately. The remaining six studies did not specify these components. Thirty-three studies used three-dimensional motion capture and/or an instrumented treadmill or ground-mounted force plate to measure running biomechanics, four studies used photoelectronic cell systems, contact laser platforms, or an optical measurement system. The remaining studies used accelerometry (*k* = 2), two-dimensional measurements (*k* = 10), and other methods such as surface electromyographic electrodes (*k* = 5) or electro goniometers (*k* = 1).

### Risk of Bias in Studies

**Fig. 2:**
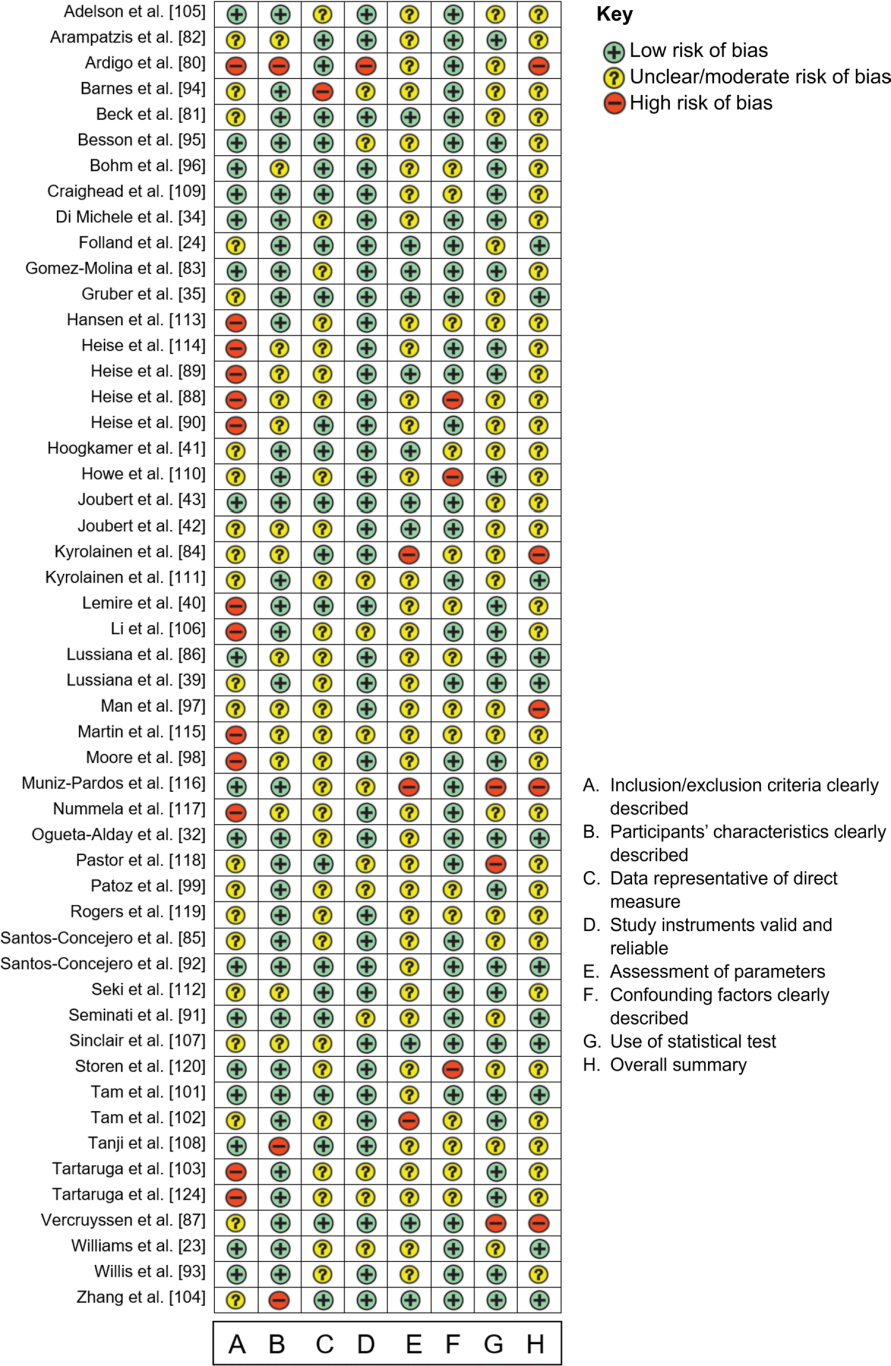
Risk-of-bias assessment for all included studies

The risk-of-bias score of included studies is reported in Fig. 2. Most studies were at moderate or high risk of bias for not clearly describing the inclusion and exclusion criteria. Conversely, only a few studies were at high risk of bias for the data collection and analysis procedures.

### Spatiotemporal Outcome Measures

**Fig. 3:**
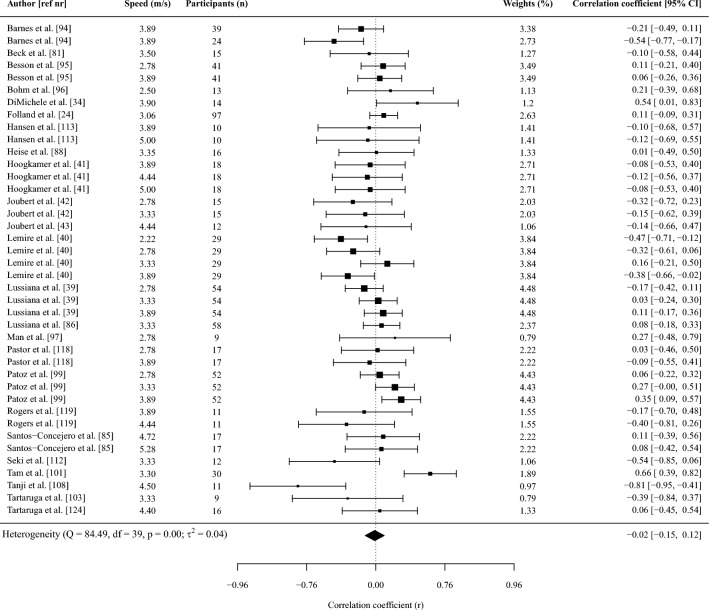
Random-effects meta-analysis of the correlation between ground contact time and running economy. Positive correlations indicate that longer contact times are associated with a higher oxygen or energy cost of running, or that shorter contact times are associated with a lower oxygen or energy cost (i.e., longer is higher or shorter is lower), while negative correlations indicate that a shorter contact time is associated with a higher oxygen or energy cost or that a longer contact time is associated with lower oxygen or energy cost (i.e., longer is lower or shorter is higher). *CI* confidence interval. Note that the correlation coefficients are depicted on a non-linear scale to ensure symmetric confidence intervals after the back transformation procedure

**Fig. 4:**
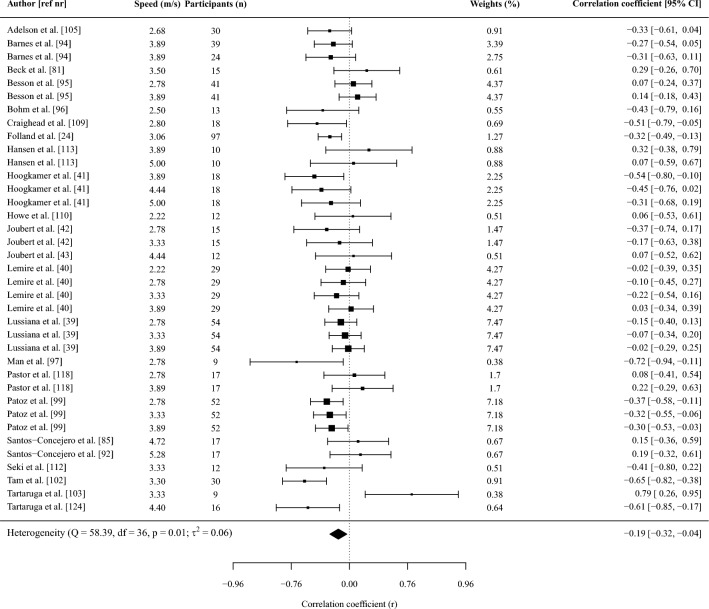
Random-effects meta-analysis of the correlation between stride frequency and running economy. Positive correlations indicate that higher step/stride frequencies are associated with a higher oxygen or energy cost of running or that lower step/stride frequencies are associated with a lower oxygen or energy cost (i.e., higher is higher or lower is lower), while negative correlations indicate that a lower step/stride frequency is associated with a higher oxygen or energy cost or that a higher step/stride frequency is associated with lower oxygen or energy cost (i.e., higher is lower or lower is higher). *CI* confidence interval. Note that the correlation coefficients are depicted on a non-linear scale to ensure symmetric confidence intervals after the back transformation procedure

**Fig. 5:**
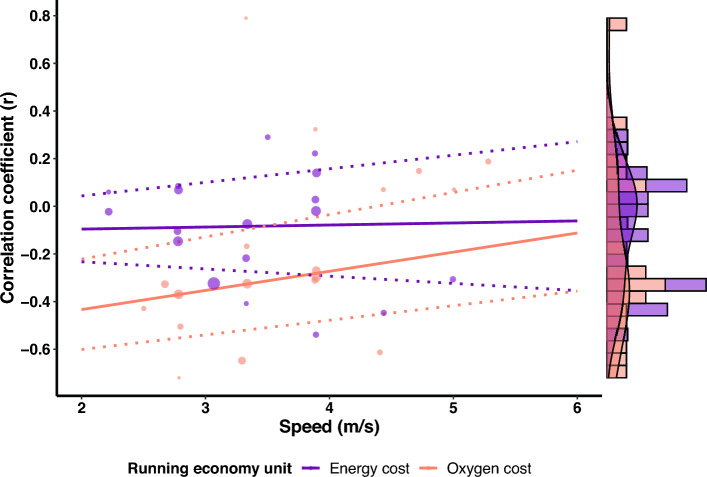
Meta-regression of the correlation between stride frequency and running economy as a function of running speed and with running economy expressed as the energetic or oxygen cost. The meta-regression also included shoe standardization as a categorical covariate. The s*olid line* represents the mean effect, while the *dashed lines* indicate the 95% confidence intervals. *Circles* represent individual correlations, with the size of the circle representing the weight of the effect to the meta-regression. The *stacked bars* on the right side depict the distribution of the categorical data (i.e., oxygen or energy cost)

**Fig. 6:**
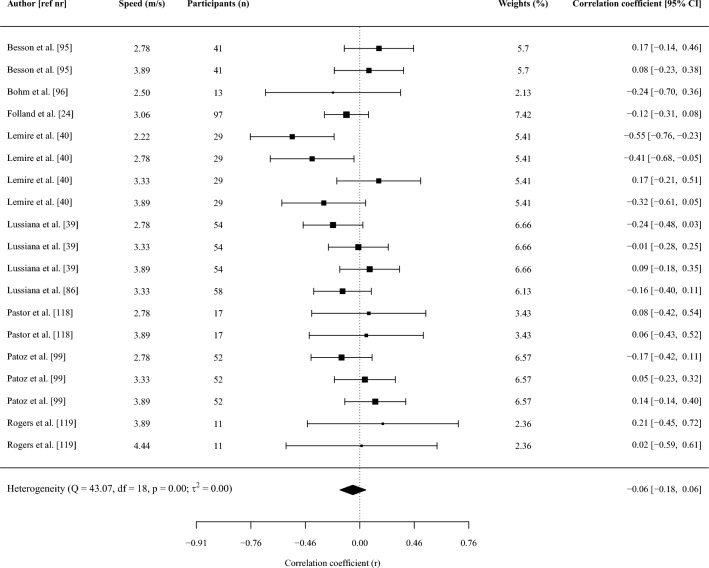
Random-effects meta-analysis of the correlation between duty factor and running economy. Positive correlations indicate that higher duty factors are associated with a higher oxygen or energy cost of running or that lower duty factors are associated with a lower oxygen or energy cost (i.e., higher is higher or lower is lower), while negative correlations indicate that a lower duty factor is associated with a higher oxygen or energy cost or that a higher duty factor is associated with lower oxygen or energy cost (i.e., higher is lower or lower is higher). *CI* confidence interval. Note that the correlation coefficients are depicted on a non-linear scale to ensure symmetric confidence intervals after the back transformation procedure

**Fig. 7:**
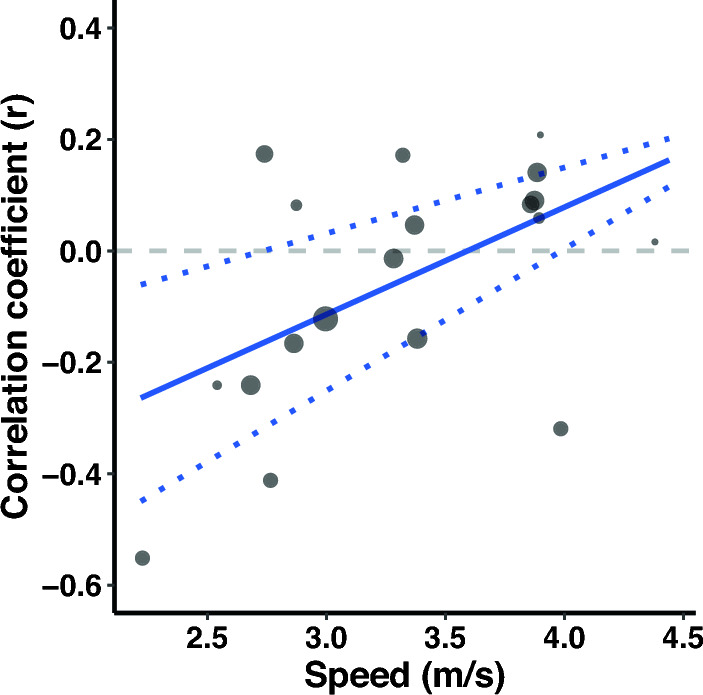
Meta-regression of the correlation between duty factor and running economy as a function of running speed. The *solid blue line* represents the mean effect, while *dashed blue lines* indicate the 95% confidence intervals. *Circles* represent individual study correlations, with the size of the circle representing the weight of the study in the meta-regression

Among the spatiotemporal outcomes investigated (contact time [Fig. 3], flight time, swing time, stride time, duty factor, stride length, normalized stride length, and stride frequency), only stride frequency was significantly associated with RE (Table 2, Figs. 4, 5, 6, and 7). The results of the meta-regressions are detailed in Supplementary File S5 (see ESM) and two meta-regression examples are shown in Figs. 5 and 7.

**Table 2:** Summary of meta-analysis findings and quality of evidence synthesis

Outcome	Summary of findings					Quality of evidence synthesis (GRADE)			
	*k*	*n*	Weighted mean correlation coefficient (95% CI)	*I*^2^ (%)	Direction effect	Imprecision	Inconsistency	Risk of bias	Overall quality
*Spatiotemporal*									
Contact time	40	591	− 0.02 (− 0.15 to 0.12)	52.3	↔	None	− 1	None	Moderate
Flight time	18	242	0.11 (− 0.09 to 0.32)	25.8	↔	None	None	None	High
Swing time	15	320	0.12 (− 0.13 to 0.36)	75.1	↔	None	− 1	None	Moderate
Stride time	12	213	0.01 (− 0.48 to 0.50)	90.2	↔	None	− 1	None	Moderate
Duty factor	19	372	− 0.06 (− 0.18 to 0.06)	15.0	↔	None	None	None	High
Stride length	19	207	0.12 (− 0.13 to 0.36)	64.1	↔	None	− 1	None	Moderate
Norm. stride length	8	160	0.27 (− 0.23 to 0.65)	67.8	↔	None	− 1	None	Moderate
Cadence	37	593	− 0.20 (− 0.35 to − 0.05)	60.3	↓	None	− 1	None	Moderate
*Vertical oscillation*									
Vertical oscillation	23	317	0.35 (0.19 to 0.49)	56.0	↑	None	− 1	None	Moderate
Vertical oscillation normalized for step length	2	24	0.20 (− 1.00 to 1.00)	35.2	↔	− 1	None	None	Moderate
*Hip and pelvis*									
Hip angle footstrike	4	69	0.05 (− 0.75 to 0.79)	0.00	↔	− 1	None	None	Moderate
Hip angle ROM	3	124	0.21 (− 0.65 to 0.84)	4.96	↔	None	None	None	High
CoM-heel distance footstrike	7	124	0.04 (− 0.38 to 0.44)	3.41	↔	None	None	None	High
Hip angle toe-off	5	81	− 0.00 (− 0.78 to 0.78)	59.2	↔	− 1	− 1	None	Low
*Knee*									
Knee angle footstrike	8	174	− 0.02 (− 0.34 to 0.31)	53.4	↔	None	− 1	None	Moderate
Shank angle footstrike	4	151	0.07 (− 0.85 to 0.88)	44.1	↔	None	None	None	High
Peak knee flexion during stance	7	198	0.27 (− 0.14 to 0.60)	69.2	↔	None	− 1	None	Moderate
Knee angle ROM	3	124	0.23 (− 0.73 to 0.88)	31.3	↔	None	None	None	High
Knee angle toe-off	6	95	0.05 (− 0.50 to 0.57)	38.6	↔	− 1	None	None	Moderate
*Ankle and foot*									
Footstrike index/angle^a^	6	156	− 0.02 (− 0.59 to 0.55)	60.3	↔	None	− 1	None	Moderate
Ankle angle footstrike	8	174	− 0.18 (− 0.51 to 0.20)	68.6	↔	None	− 1	None	Moderate
Peak ankle angle during stance	4	67	− 0.07 (− 1.00 to 1.00)	69.1	↔	− 1	− 1	None	Low
Ankle angle toe-off	7	110	0.13 (− 0.35 to 0.55)	56.9	↔	None	− 1	None	Moderate
*Kinetic*									
Absolute vertical ground reaction force	4	112	− 0.27 (− 0.66 to 0.23)	0.00	↔	None	None	None	High
Normalized vertical ground reaction force	4	48	0.28 (− 0.49 to 0.81)	0.00	↔	− 1	None	− 1	Low
Vertical stiffness	18	236	− 0.31 (− 0.56 to − 0.05)	68.2	↓	None	− 1	None	Moderate
Leg stiffness	18	287	− 0.28 (− 0.52 to − 0.03)	68.2	↓	None	− 1	None	Moderate
Knee stiffness	2	60	0.11 (− 1.00 to 1.00)	40.4	↔	− 1	None	None	Moderate
Ankle stiffness	2	60	0.10 (− 1.00 to 1.00)	46.5	↔	− 1	None	None	Moderate
Total mech. work	8	54	0.37 (− 0.05 to 0.68)	0.00	↔	− 1	None	None	Moderate
Negat. mech. work	3	22	0.08 (− 1.00 to 1.00)	26.8	↔	− 1	None	None	Moderate
Posit. mech. work	3	22	0.18 (− 0.96 to 0.98)	0.00	↔	− 1	None	None	Moderate
*Muscle activity*									
EMG gluteus maximus stance	2	27	− 0.10 (− 0.78 to 0.70)	0.00	↔	− 1	None	None	Moderate
EMG biceps femoris stance	4	73	− 0.09 (− 0.46 to 0.32)	0.00	↔	− 1	None	None	Moderate
EMG gastroc. Stance	4	57	− 0.03 (− 0.89 to 0.88)	26.3	↔	− 1	None	None	Moderate
EMG rectus femoris stance	7	131	− 0.34 (− 0.79 to 0.36)	89.1	↔	None	− 1	None	Moderate
EMG tibialis anterior stance	4	115	− 0.08 (− 0.28 to 0.13)	0.00	↔	None	None	None	High
EMG soleus stance	2	27	0.02 (− 0.74 to 0.76)	0.00	↔	− 1	None	None	Moderate
EMG vastus lateralis stance	2	28	− 0.46 (− 1.00 to 1.00)	31.3	↔	− 1	None	None	Moderate

Only outcomes with *k* > 1 are included in this table

↔ indicates no significant effect; ↓ indicates significant negative correlation; ↑ indicates significant positive correlation

*CI* confidence interval, *CoM* center of mass, *EMG* electromyographic activity, *gastroc* gastrocnemius, *GRADE* Grading of Recommendations Assessment, Development and Evaluation, *k* number of outcomes, *mech* mechanical, *n* number of participants, *norm* normalized, *ROM* range of motion

^a^This effect size represents a standardized mean difference instead of a correlation coefficient

### Vertical Oscillation

A higher vertical oscillation of the pelvis/trunk/center of mass showed a moderate, significant association with a higher oxygen/energetic cost (poorer RE; *r* = 0.35, Supplementary File S6, Fig. S1, see ESM).

### Kinematic Outcomes

Ankle, knee, and hip angles at different phases in the gait cycle and their range of motion were not significantly associated with RE (Table II, Supplementary File S6, Figs. S4–S6, see ESM). Similarly, segment angles relative to the global reference frame were not associated with RE (Table 2). Meta-regression results are detailed in Supplementary File S5 (see ESM).

### Kinetic Outcomes

Peak vertical ground reaction forces were not significantly associated with RE overall (*r* = − 0.02, or when considering absolute *r* = − 0.27 or normalized *r* = 0.28 outcomes separately; Table 2). Meta-regression was not undertaken for these outcomes due to insufficient effects. Similarly, a higher vertical and leg stiffness were both associated with a moderate and small significant reduction in oxygen/energetic cost (*r* = − 0.31 and − 0.28, respectively, Supplementary File S6, Figs. S2–S3, see ESM). The results of the meta-regressions are detailed in Supplementary File S5 (see ESM).

One study further reported a higher anterior–posterior and medio-lateral impulse to be non-significantly associated with a higher energy cost (*r* = 0.25 and 0.37, respectively) [88]. Storen and co-workers [120] reported no significant correlations between the braking or propulsive forces (correlation magnitudes not reported), but runners with a higher oxygen cost showed a higher sum of the horizontal and vertical forces (*r* = 0.66). Similarly, Williams and Cavanagh [23] reported the group with the lowest oxygen cost exhibited lower horizontal braking forces than the group with medium oxygen cost, which in turn exhibited lower braking forces than the group with high oxygen cost, although no differences reached statistical significance. One final study reported a trivial correlation between average braking forces and energy cost (*r* = 0.07), but a moderate correlation between average propulsive force and energy cost (*r* = 0.30) [81].

### Mechanical Work Outcomes

Total, negative, or positive mechanical work were not significantly associated with RE (Table 2). Meta-regression was not undertaken due to insufficient effects.

### Electromyographic Outcomes

Surface electromyographic activation of the gluteus maximus, soleus, gastrocnemius medialis/lateralis, rectus femoris, vastus lateralis, biceps femoris long head, and tibialis anterior during stance was not significantly associated with RE (Table 2). Meta-regression was not undertaken due to insufficient effects.

### Between-Group Comparisons

The only between-group comparison with sufficient data for meta-analysis was footstrike angle. Meta-analysis showed that RE did not significantly differ between footstrike classified into rearfoot or midfoot/forefoot strikers (standardized mean difference = − 0.02, Table 2, Supplementary File S6, Fig. S7, see ESM).

### Sensitivity Analysis

We performed an informal sensitivity analysis to compare whether the correlations between various biomechanics and RE were affected by the expression of RE as oxygen or energetic cost within the same study by comparing the correlation coefficients (Supplementary File S3, see ESM). These analyses revealed that the correlations were very similar with both analyses (mean difference of *r* = − 0.03 and 0.01 for contact time and cadence, respectively). Additionally, we compared the sensitivity of the correlation coefficients to two different stoichiometric equations. This resulted in negligible mean differences of *r* = 0.01.

## Discussion

The primary aim of this systematic review and meta-analysis was to synthesize the available evidence on the association between running biomechanics and RE as investigated in observational studies. In the following sections we first discuss the most important findings from the meta-analyses and (to a lesser extent) individual study results, and we discuss potential biomechanical/physiological mechanisms for each of the observed effects. We also briefly compare the associations found in our review of observational studies with findings from longitudinal studies that investigated changes in running biomechanics and RE, and with studies that investigated associations between running biomechanics and running performance. Finally, we provide practical implications of our findings for coaches, researchers, and developers of wearable technology. Note that we also discuss non-significant correlations as ‘trends’ when their magnitude is at least small (*r* > 0.1) and when the correlation is consistent with other significant biomechanical outcomes. For example, a larger vertical oscillation could result from a larger knee flexion during stance, but the latter may not be significant due to the small number of studies assessing this outcome.

### Spatiotemporal Outcomes

Moderate GRADE evidence showed that a higher stride frequency was weakly but significantly (*r* = − 0.20) associated with a lower energetic cost of running (Table 2, Fig. 4). Between-group studies included in this review that compared cadence between groups differing in RE also reported a higher cadence in the more economical group [83], or no difference between groups [82]. At the fixed running speed used in all included articles, a low stride frequency means that runners adopt a long stride length, which can lead to higher braking impulses [125]. Greater braking during the initial phase of the stance phase necessitates a larger horizontal propulsive force production during the remaining stance phase to reaccelerate the center of mass to maintain running speed. The generation of propulsive force involves energetically expensive concentric contractions and is an important component of the metabolic cost of running [24, 126]; see also Sect. 4.4. These mechanisms may therefore partly explain the association between a higher stride frequency and lower energy cost. However, the small magnitude of the correlation between stride frequency and RE should be noted. The small magnitude observed may be due to differences in anthropometrics (e.g., height and body mass) between individuals that in turn can cause differences in the most economical stride frequency between individuals [46–49], and thereby reduce the magnitude of the correlation. We explored whether more homogeneous groups showed stronger correlations between stride frequency and RE by performing a meta-regression with the coefficients of variation in height or body mass as continuous predictors (Supplemental File S5, see ESM). However, this indicated that the correlation between stride frequency and RE did not differ with smaller coefficients of variation in height and body mass (i.e., reflecting a more homogeneous group). Therefore, a higher stride frequency is weakly associated with better RE, but the homogeneity of anthropometrics across studies does not appear to alter the strength of the observed relationship. Running speed and running economy units both significantly moderated the correlation between cadence and RE (Supplemental File S5, Fig. 5), such that the correlation became larger with increases in speed, and decreased with RE expressed as oxygen cost.

Based on (a) the significant association found between stride frequency and RE, and (b) the inverse relationship between stride length and stride frequency at a given running speed, one would expect a shorter stride length also to be associated with a lower energy cost. While the direction of the effect did indeed suggest shorter stride lengths were associated with a lower energy cost (*r* = 0.12; moderate GRADE evidence), the association was not significant. This is likely due to the ~ 65% smaller sample size and thus lower statistical power in the stride length analysis compared with the stride frequency analysis (Table 2). A similar reason likely explains the lack of significant associations for stride length normalized to height. Interestingly, the correlation for normalized stride length was approximately double compared with stride length (*r* = 0.27; moderate GRADE evidence). This suggests that individuals with short stride lengths relative to their height might have better RE than those with long stride lengths relative to their height. Stride length normalized to height may therefore be more relevant to inform on running economy, and potentially presents a useful variable to modify when optimizing running economy, although further research is required to substantiate this notion.

Stride time, ground contact time (Fig. 3), flight time, and swing time were not significantly and mostly trivially associated with RE (moderate–high GRADE evidence). One between-group study included in this review also found no differences in these outcomes between groups differing in RE [82]. Combining these findings with the significant correlation observed between stride frequency and RE suggests that the higher stride frequency seen in more economical runners may be achieved using different combinations of contact and flight times that are equally economical. This supports the notion that contact time can be self-optimized and operates within a narrower optimal range than stride frequency [30]. Mechanistically, the trivial association between contact time and RE may be because a shorter contact time requires a faster force production, which in turn requires a higher fascicle/fiber shortening velocity and this increases energy costs [22, 127]. Conversely, a longer contact time may lead to more dissipation of stored elastic energy into heat (e.g., [128]), and may reflect a longer braking phase and thus higher braking impulse, both of which require metabolically expensive concentric muscle action to generate propulsive force to maintain a similar running speed. The trivial correlation between contact time and RE is notable as it is often believed that a short contact time is reflective of better RE based on the findings of several studies in highly trained runners [100, 129]. However, these studies either did not standardize running speed [129], or measured RE above the anaerobic threshold [100], and our findings suggest that this effect is not observed when running speed is standardized and when measured at a metabolic steady state.

In line with the findings for contact and flight time, duty factor (i.e., contact time/[contact time + flight time]; reflecting the proportion of stride time spent on the ground) was also not significantly associated with RE (high GRADE evidence, Table 2). Mechanistically, this may be because runners with a low duty factor (e.g., long flight time) rely more on a larger vertical displacement (which increases energy cost), but also better use elastic energy (i.e., optimization of the spring-mass model; which reduces energy cost), while runners with a high duty factor rely more on energetically costly forward propulsion, but also limit energetically costly vertical displacement [39, 130]. The net effect is therefore that both lower and higher duty factors can be economical. Whether a runner adopts a lower or higher duty factor may in turn reflect differences in musculotendinous properties. Runners with a low duty factor (longer flight time, shorter contact time) have, for example, been shown to exhibit a higher rate of force development, muscle activation, and H-reflex of the soleus compared with runners with a high duty factor [131]. Runners may therefore simply self-organize to the technique that is most economical for their musculotendinous properties with sufficient training (e.g., [27]). Simply altering contact or flight time (and thus duty factor) without determining if a runner already produces a metabolically optimized running gait may force them to use a technique that is not economical for their current musculotendinous properties and could reduce rather than increase performance. Further research is required to investigate whether alterations in musculotendinous properties could allow individuals to use a different running technique that in turn is more economical.

Our meta-regression, however, indicated that the correlation between duty factor and RE became significantly larger (from moderate negative correlations to small positive correlations) with increases in running speed (Supplementary File S5, see ESM). This suggests that high duty factors (shorter contact times and/or longer flight times) are associated with lower energy costs at relatively slower speeds, but higher energy costs at higher speeds. This may be explained as follows: at relatively slow running speeds, the contact time is relatively long (e.g., 275 ms at 2.78 m∙s^−1^), and this leads to more dissipation of elastic energy into heat as compared with shorter contact times [128]. By adopting a relatively shorter contact time and increasing the flight time at these slow speeds, less elastic energy may dissipate, thus benefiting energy costs. Conversely, at higher speeds, the contact time is already shorter (e.g., 175 ms at 4 m∙s^−1^) and further reductions in contact time may not yield much benefit from an elastic energy dissipation perspective, while they may be more energetically costly from a fascicle shortening velocity perspective, thus reducing the benefit of a high duty factor. Nevertheless, further research is required to substantiate these hypotheses.

Findings from individual studies showed that contact time imbalance was not significantly associated (*r* = − 0.05) with RE in one study [43]. Another study reported no correlation between different symmetry indexes and RE [91]. These findings are in contrast to those of a study not included in this review that found contact time imbalance to be strongly associated with poorer RE [132]. However, this study did not standardize speed, thus suggesting running speed may have confounded this association. Only one study reported on step width, with this being not significantly different between groups differing in RE [23]. This latter finding is in line with the relatively small energetic cost for maintaining mediolateral balance during running, which is estimated to account for only ~ 2% of the total energetic cost [133].

### Vertical Oscillation

Moderate GRADE level evidence showed that a higher vertical oscillation of the pelvis/trunk/center of mass during either a complete stride or stance phase was moderately associated with a higher energetic cost (poorer RE; *r* = 0.35, Supplementary File S6, Fig. SI, see ESM). One study identified by our systematic search compared vertical displacement between groups of runners differing in RE and found vertical oscillation also to be lower in more economical groups, although the difference was not significant [23]. From a physiological perspective, the correlation between vertical oscillation and RE can be explained by a higher vertical oscillation requiring recruitment of a larger muscle volume to produce a larger vertical impulse, which increases energy cost. From a mechanical perspective, higher vertical oscillation will contribute to greater work needing to be performed against gravity, and thus a greater energetic demand being placed on a runner. Meta-regression analyses showed no moderation of the effect with RE expressed as oxygen cost or energetic cost, or gross versus net oxygen/energetic cost (Supplemental File S5, see ESM). Similarly, meta-regression showed that running speed did not significantly moderate the association between RE and vertical oscillation (Supplementary File S6, Fig. SI, see ESM), although there appeared to be a trend within each study for stronger associations with increases in speed (Supplementary File MOESM1, Fig. SI, see ESM). Meta-regression with standardized versus non-standardized shoes showed that the correlation between RE and vertical oscillation increased when shoes were standardized, suggesting that some shoe features such as the degree of cushioning may affect vertical oscillation and thereby RE. In support of this, removing shoe cushioning has been shown to reduce vertical oscillation and improve RE [134] and net mechanical efficiency [135]; this may explain the smaller correlation when shoes were not standardized. When vertical oscillation was normalized to step length, the correlation became non-significant, yet the magnitude of the effect was in the same direction (i.e., larger normalized step length is higher energy cost; *r* = 0.20). The absence of a significant effect likely reflects the small number of studies (*k* = 2) that measured this outcome. The slightly smaller magnitude of the normalized vertical oscillation suggests that part of the higher energy cost with higher vertical oscillation is related to the resulting larger step length and thus higher oscillation during the flight phase, while the remaining part results from larger vertical displacement during the stance phase. Collectively, these different measures therefore all reflect that a smaller vertical oscillation is typically associated with a better RE.

### Kinematic Outcomes

A larger peak knee flexion and knee flexion range of motion were non-significantly associated with a higher energy cost (*r* = 0.27 and 0.23, respectively, Table 2, Supplementary File S6, Fig. S5 [see ESM], moderate and high GRADE evidence, respectively). The lack of significant associations may reflect the relatively small number of studies that investigated these outcomes and considerable inter-study variability (potentially introduced by non-standardized shoes, which may impact lower extremity stiffness [136–138]). Further, one between-group study included in this review found no differences in knee angle between groups of runners differing in RE, but shoe wear was not standardized [82]. Mechanistically, a higher knee flexion angle or range of motion during mid-stance creates a larger knee extension moment, meaning greater muscle force needs to be produced potentially through recruiting a larger muscle volume, which in turn may increase energy costs. A small training study supports this notion, with reductions in the knee extensor moment as runners became more economical [26]. Further, studies that had individuals adopt larger knee flexion during running also reported increases in energy cost [139]. As a larger knee flexion (range of motion) is expected to increase vertical displacement during stance, this may also partly explain the association between vertical displacement and RE. Nevertheless, some findings suggest that the knee extensors may function on the ascending limb of the force–length curve at knee angles similar to those observed during the stance phase in running [140], and a slightly larger knee flexion angle could therefore result in more force potential, which reduces activation and energy cost. This may explain why one study that compared groups of runners differing in RE found a trend towards a larger peak knee flexion angle in the more economical group, although the difference was not significant [23]. The knee angle at footstrike and toe-off both showed trivial and non-significant associations with the energetic cost of running (Table 2), suggesting the trend for a higher energy cost with a larger knee flexion range of motion may be due to a variable combination of joint angles, with some individuals landing with a relatively extended knee at footstrike followed by large flexion, while other individuals may instead extend their leg more at toe-off following large flexion. One study further reported a significant correlation (*r* = 0.41) between a higher peak knee flexion angle during the swing phase of running and a higher energy cost [24]. While a larger knee flexion reduces the leg moment of inertia, it may also speculatively delay the coupled swing-leg retraction of the front leg, with this delay potentially leading to higher braking forces and thereby a higher energetic cost than the larger moment of inertia.

Similar to the knee, a larger hip range of motion during the stance phase showed a small but non-significant association with a higher energy cost (*r* = 0.21; high GRADE evidence; Table 2), although one between-group study included in this review showed no differences between groups differing in RE [82]. Mechanistically, a larger hip range of motion requires either a larger flexion at or just after initial contact, or greater extension at toe-off. However, the hip angle at footstrike and toe-off both showed trivial and non-significant associations with RE (Table 2), suggesting runners may use a combination of strategies to realize this larger range of motion. Alternatively, the degree to which runners flex their hips after initial contact may better correlate with RE as this could reflect a high braking impulse, although this contention requires further research. Hip and knee angles at footstrike are often measured to infer whether an individual is ‘overstriding’ based on the premise that this increases injury risk and decreases RE (e.g., [125, 141]). However, our findings do not support that either joint angle in isolation is associated with RE. Moreover, a measure that integrates both angles into one outcome (i.e., heel to the extrapolated center of mass horizontal, anterior posterior distance) also showed a trivial association (*r* = 0.04) with RE, thus questioning whether a combination of both angles is more sensitive for inferring RE than either measure alone. Similar trivial associations (*r* = − 0.09, 0.06, and 0.11 at 2.78, 3.33, and 3.89 m∙s^−1^) were reported by one study when heel to the center of mass distance was normalized to leg length [39]. Likewise, shank or thigh angles relative to the global reference frame were also not significantly associated with RE (Table 2), with one study even showing a more horizontal shank angle in more economical runners [23]. Collectively, these findings suggest that lower limb orientation at initial contact plays only a minimal role in contributing to RE. Future research could explore whether a combination of angles and velocities at initial contact may better correlate with RE than joint angles alone. Moreover, future studies could also investigate if biomechanical outcomes at midstance may better relate to RE than at initial contact.

The ankle angles at footstrike, toe-off, or the peak during stance were all not significantly associated with RE (Table 2, Supplementary File S6, Figs. S4–6, see ESM), although a more plantar flexed ankle at toe-off showed a small non-significant association with a higher energy cost (*r* = 0.13). This is in line with previous findings whereby more economical runners showed smaller ankle plantar flexion at toe-off [23] and reductions in ankle plantar flexion at toe-off were observed when runners became more economical [27]. Positioning the ankle in less plantar flexion may optimize the force–length potential [142] and thereby both reduce activation-related energy cost and aid the production of horizontal force during the propulsive phase [27], hence making it an economical running characteristic. Additionally, larger plantar flexion angles at toe-off may require a metabolically costly concentric muscle action and thereby explain the small association with higher energy cost. Finally, a larger plantar flexion at toe-off may result in a larger vertical oscillation as runners are using their plantar flexors to push upwards, and this may also partly explain the association between vertical oscillation and RE.

Our between-group meta-analysis showed no significant differences in RE between rearfoot or fore/midfoot strikers (Hedges’ *g* = − 0.02; moderate GRADE evidence, Table 2, Supplementary File S6, Fig. S7, see ESM). In line with these findings, two studies included in this review reported a trivial correlation (*r* = 0.08 and 0.10) between RE and footstrike angle [24, 86]. Therefore, these findings do not support the use of footstrike patterns to infer RE as used by some coaches [143]. Mechanistically, the similar RE between different footstrike patterns can be explained by the reduced muscle energy cost associated with lower fascicle contraction velocity in fore/midfoot strikers being counteracted by greater muscle forces during early ground contact, thus yielding no net benefit to RE [144]. While pronation of the subtalar joint is often investigated in relation to injury risk, only one study investigated its association with RE [124], reporting a small but non-significant correlation (*r* = 0.12; *p* = 0.65) between higher pronation and higher energy cost. The trivial associations between ankle angles and foot orientation at initial contact and RE further support the notion that initial contact kinematics have a minimal role in RE, potentially due to the small muscle forces at this time instant. Conversely, our findings regarding toe-off plantar flexion indicate that the lower limb orientation during propulsion may play some role in RE.

Only a few studies investigated trunk or upper limb kinematics in relation to RE and the difference in the measured components did not allow combination in a meta-analysis. No studies reported breast motion. One study of 97 runners reported that a greater trunk lean range of motion was significantly associated with a higher energy cost (*r* = 0.32), while a greater trunk lean angle relative to the global reference frame was non-significantly associated with a higher energy cost (*r* = 0.27) [24]. Moreover, a greater pelvis/trunk rotation (i.e., longitudinal body rotation) was associated with higher energy cost in the same study (*r* = 0.32) [24]. However, another study found no significant differences in trunk rotation between groups differing in RE and found rather greater trunk flexion in a more economical group [23]. These conflicting findings may be related to the smaller number of subjects in the latter study [23] versus the former [24], and because the latter study [23] split the sample into three groups which reduced statistical power. Tartaruga et al. [124] found a moderate but non-significant correlation (*r* = 0.42; *p* = 0.11) between a larger elbow range of motion and oxygen cost. Similarly, Williams and Cavanagh [23] showed greater arm movement (as measured by three-dimensional wrist displacement) in a group of runners with higher $\dot{V}{\text{O}}_2$ as compared with a group with lower $\dot{V}{\text{O}}_2$, although the difference was not significant. Mechanistically, arm movement reduces the lateral movement and longitudinal rotation of the body by counteracting the angular momentum created by the swinging legs. Because greater pelvis/trunk rotation (i.e., longitudinal body rotation) has been associated with higher energy cost [24], greater arm movement may be associated with a higher energy cost via a compensatory mechanism whereby greater arm movements compensate for greater trunk rotation, with this indirect association possibly explaining the non-significant nature of the findings in individual studies with small sample sizes. The lack of studies and use of inconsistent biomechanical outcomes means future studies should focus on the relationship between the trunk, breast, upper limb, and pelvis kinematics, and RE.

### Kinetic Outcomes

High GRADE level evidence indicated that a higher absolute peak vertical ground reaction force showed a small non-significant negative association with a lower energy cost (*r* = − 0.27). Conversely, when the vertical ground reaction force was normalized for body mass, there was a small non-significant positive correlation (*r* = 0.28; low GRADE evidence), suggesting that higher peak vertical ground reaction forces relative to body weight were associated with higher energy costs. Individual study outcomes indicated that the normalized peak vertical ground reaction force was higher in a group of runners that showed a higher oxygen cost as opposed to a group of runners with lower oxygen cost [23]. Further, Heise and Martin [88] reported that a larger net vertical impulse was associated (*r* = 0.60) with a higher oxygen cost. The energy required to support and accelerate the body has been suggested to account for ~ 80% of the energy cost of running [133]. Mechanistically, a larger vertical ground reaction force peak relative to body weight reflects a larger vertical acceleration (as force/mass = acceleration) that in turn requires the recruitment of more muscle mass, which increases energy cost. This larger vertical acceleration is likely to also lead to a larger vertical oscillation and this finding is therefore consistent with the relationship found between vertical oscillation and RE.

In line with the lower normalized peak vertical ground reaction force and lower vertical oscillation being associated with a lower energetic cost, a larger vertical stiffness and leg stiffness also showed a small significant association with a lower energy cost (*r* = − 0.31, and − 0.28, respectively; moderate GRADE evidence). Having a high vertical stiffness and leg stiffness may optimize storage and release of elastic energy and thereby benefit RE. In contrast, knee and ankle stiffness were not associated with RE (Table 2). This may be due to variations in mechanisms regarding how leg stiffness is produced, with runners utilizing different lower limb segment orientations to produce similar magnitudes of leg stiffness (e.g., different degrees of hip and knee flexion). Consequently, leg and/or vertical stiffness may be more informative for inferring RE than the stiffness of individual joints. In line with these findings, Burns and colleagues [145] re-analyzed data from two studies included in this review and showed that more economical runners exhibited a technique that was more similar to an ideal spring-mass system than recreational runners.

Studies have shown a tendency towards higher peak forces and or impulses in the anterior–posterior direction to be related to a higher oxygen cost [81, 88, 120] or to be present in runners with a worse RE than those with a good RE [23]. However, limited significant findings exist. A body of research by Arellano and Kram [133] has identified propulsive forces to be more metabolically costly to generate than braking forces using repeated-measures study designs. Collectively, minimizing propulsive force generation may be important for economical running, but due to the need to balance braking and propulsion during constant speed running, this will also involve minimization of braking forces.

### Mechanical Work Outcomes

Since it is difficult to isolate the effect of one biomechanical factor on RE, some studies have used a more global measure that involves estimation of the mechanical work done to move the center of mass, and work done relative to the center of mass. Specifically, external mechanical work refers to the movement of the whole-body center of mass relative to the ground, whereas internal mechanical work refers to the movement of the arms and legs relative to the whole-body center of mass. Total mechanical work in turn reflects the sum of both external and internal work. Surprisingly, none of the mechanical work variables were significantly associated with RE. Nevertheless, the directions of the effects were consistent with the notion that more mechanical work should reflect more metabolic work as a higher total mechanical work was moderately but non-significantly associated with a higher energy cost (*r* = 0.37). The lack of significant findings for these outcomes likely reflects the small number of studies (with each also having a small sample size) that investigated these outcomes (Table 2), and different methods that can be used to calculate mechanical work [146].

Two studies have shown that more economical runners had more energy transfer between adjacent segments [115] or between the trunk and legs [23]. This suggests that more economical runners are better able to use the energy-transporting role of bi-articular muscles, which in turn requires less muscle work to displace the center of mass. However, more research is required to detail which exact components (e.g., joint coupling) allow for this better energy transport.

### Muscle Activation Outcomes

Surface electromyographic activation of various mono- and biarticular lower limb muscles during stance was not significantly associated with RE (Table 2, moderate-high GRADE evidence), with the correlation for all, except for two muscles (rectus femoris and vastus lateralis) also being trivial. The general lack of associations between muscle activation and RE could be considered surprising because, from a physiological perspective, higher muscle activation is often considered detrimental to RE as it is believed to reflect a larger number of active cross-bridges and ion pumping and thus higher energy costs [147]. A potential explanation for this finding is that some muscle activation may be required to increase leg and vertical stiffness and improve storage and re-use of elastic energy in tendons, both of which indirectly improve RE despite the higher muscle activation also (directly) resulting in higher energy costs. In direct support of this hypothesis, the rectus femoris and vastus lateralis both play an important role in knee extension and thus vertical stiffness, and higher activation of these muscles showed a (non-significant) moderate magnitude correlation with lower energy costs. This may be because higher activation of these muscles helps increase lower limb stiffness. Indeed, it has previously been suggested that greater pre-activation of the leg extensors increases the sensitivity of the muscle spindle through enhanced alpha-gamma coactivation potentiating stretch reflexes, and this may increase musculotendon stiffness and thereby enhance RE [148]. In further support, one study included in this review found earlier onset of rectus femoris muscle activation was associated with a lower oxygen cost [114]. In contrast, measures of tendon stiffness were not associated with joint stiffness during hopping [149] or joint angles at initial contact during running [108]. Similarly, groups differing in Achilles tendon stiffness showed no significant differences in ankle joint kinematics during running [82]. Such findings further reinforce the importance of muscle activation strategies to joint range of motion and hence more global measures of stiffness such as vertical or leg stiffness.

While several studies also reported co-activation outcomes, these were too dissimilar to include in meta-analysis. Specifically, four studies [86, 98, 101, 102, 114] investigated either the percentage of stance during which muscles were co-activated or the ratio of muscle activation. The duration of the stance phase over which muscles were co-activated showed inconsistent relations with RE, with one study reporting trivial correlations [86] (obtained from individual participant data; see the ‘overview’ tab in the supplementary Excel dataset for all correlations in the ESM), another study reporting generally (very) strong associations between longer co-activation duration and a higher oxygen cost [98], and another study reporting longer co-activation durations relative to stance or swing phase generally being associated with lower oxygen cost [114]. These conflicting findings may reflect differences in methods used to determine co-activation duration and pairs of muscles investigated. A higher ratio of co-activation (i.e., both muscles being activated at the same time to a larger extent relative to their maximum) was associated with a higher energy cost in two studies from the same author group [101, 102].

### Practical Implications for Athletes, Wearable Technology, and Researchers

**Fig. 8:**
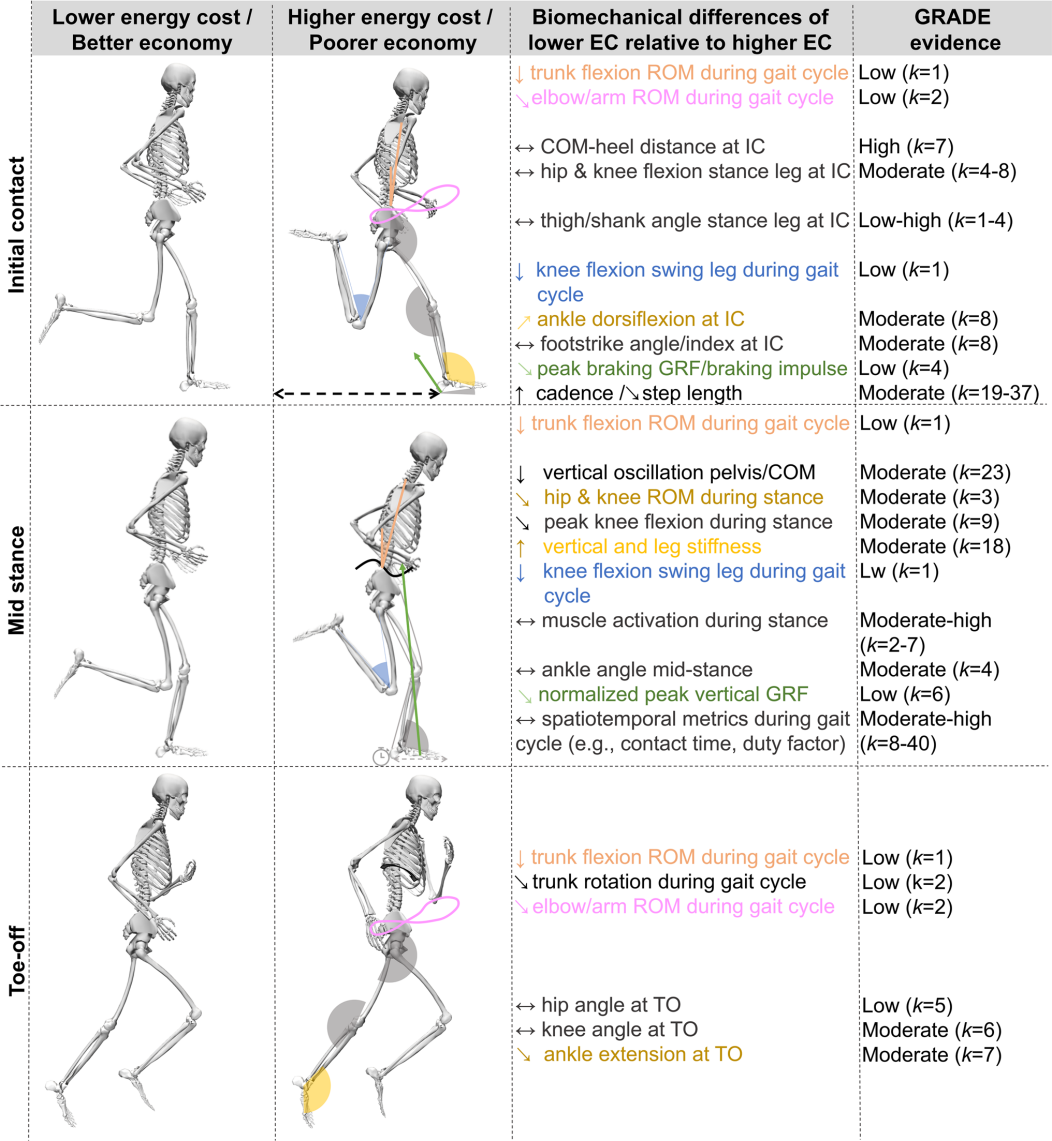
Schematic visualization of the most important associations between running economy and running biomechanics from meta-analyses and individual study results. ↓ or ↑ denote statistically significant correlations, or differences in biomechanics between groups differing in running economy, or differences in running economy between groups differing in running biomechanics, with ↑ indicating more economical runners have larger magnitudes for the specific component. ↔ denotes a component that is not significantly different and of trivial magnitude. Oblique arrows depict non-significant trends of at least a small magnitude (*r* > 0.1). *k* = number of effect sizes for the specific outcome. Note that for some outcomes, the number of effect sizes in this figure is different from the number of effect sizes in Table 2 as the figure also includes effect sizes from individual (group-comparison) studies, while Table 2 solely reflects correlational studies included in the meta-analysis. *COM* center of mass, *EC* energy cost, *GRF* ground reaction force, *IC* initial contact, *ROM* range of motion, *TO* toe-off

The findings of this review have several implications for coaches, athletes, researchers, and developers of wearable technology. Most prominently, Fig. 8 depicts a selection of running technique components that were found to be associated with RE in this review. These components may be modified using targeted training interventions in an attempt to improve RE and by extension running performance. A consideration in this regard is that most correlations were small to moderate in magnitude at most (*r* = 0.2–0.35), which would equate to an explained variance and potential improvement in running economy of (only) 4–12%. However, even a 4% increase is considered relatively large and relevant in shoe comparison studies. Moreover, the magnitude of explained variance may increase when combining different modifications. Nevertheless, to date, targeted training interventions to improve RE have produced mixed results. Specifically, modifying certain components of running technique (e.g., Pose^®^ running or changing footstrike) has been shown to not change RE [109, 150–155], or even worsen it [156]. Conversely, changing stride frequency has improved RE [157, 158], with one study specifically training runners to alter their stride frequency towards their most economical (mathematically optimal) stride frequency [158]. Given that most runners produce stride frequencies lower than their mathematical optimal stride frequency [28–30, 48, 159] and stride frequency was negatively associated with RE in our meta-analysis, wearables and coaches could target stride frequency increases in an attempt to improve RE. In turn, this higher stride frequency likely reduces vertical oscillation [160, 161], which might also enhance RE according to our findings. It is important to note that more research is required to compare the effectiveness of targeted and unguided training at improving RE. Moore et al. [27], for example, found beginner runners self-optimized during 10 weeks of unguided running training. Specifically, RE improved by 8% and the training resulted in alterations in running biomechanics, with three kinematic variables explaining 94.3% of the change in RE.

Another observation with potential practical implications was that two studies reported better energy transfer between segments in more economical runners [23, 115]. Similarly, the findings of three studies suggested that intermuscular coordination (e.g., co-contraction duration and magnitude) were associated with RE [98, 101, 102]. Collectively, these findings suggest that exercises which may enhance intermuscular coordination could be beneficial for enhancing RE (e.g., via plyometric training/running drills or certain ballistic strength training exercises). For example, high-velocity multi-joint training (as opposed to single-joint training) may enhance intermuscular coordination and thereby energy transfer [162, 163], while high-velocity (multi-joint) training may reduce co-contraction magnitude during high-velocity tasks such as running (as opposed to an increase in co-contractions observed with heavy resistance training in some studies) [164]. Training interventions are required to confirm if improvements in RE can be produced via changes in intermuscular coordination.

In addition to informing on components that may be modified to improve RE, our results also provide information on components that may *not* be relevant to modify from an RE perspective such as contact time, flight time, and duty factor. In support of this, acute gait manipulation studies showed that most runners naturally select contact times close to their theoretical optimum value, with ground contact time also exhibiting a narrow optimal range within which runners can operate [30]. However, some components that were not associated with RE may still be relevant to modify if associations are present between running performance and the component, as optimal performance may require gait optimization beyond minimizing energy cost [24]. In support of this, it has recently been demonstrated that individuals adopt a technique that avoids overburdening individual muscles (i.e., minimization of individual muscle fatigue) during walking, despite this leading to a higher overall metabolic cost [165]. This may therefore explain some conflicting findings between our review and studies that compared running biomechanics between groups of runners differing in running performance or training experience [166–169].

### Limitations and Considerations

This review has several limitations that should be kept in mind when interpreting the results. First, most of the included studies measured males solely or predominantly (Table 1). Running biomechanics have been shown to differ between males and females (e.g., [170]) and further research is therefore required to investigate whether the findings of this review translate to females.

Second, we did not include studies among elderly subjects as it has been shown that older individuals retain their RE, despite biomechanical differences [50], and this could therefore have confounded the correlations between running biomechanics and RE.

Third, while track running and some road races are performed on a predominantly level surface, runners will typically also run in uphill and downhill conditions. However, we limited our review to level running, with a maximum of 1% treadmill inclination as an inclusion criterion. While it is unknown if these findings can be generalized to uphill and downhill conditions, recent research has shown that uphill, level, and downhill RE values are strongly related within individuals, except on steep slopes [40, 171]. Thus, runners who adopt an economical running gait in level running may also adopt an economical gait in sloped running.

A fourth limitation is that most studies that included kinematics focussed on sagittal-plane kinematics, with only two studies including some frontal-plane kinematics [107, 124]. Further research is therefore required on frontal- and transverse-plane kinematics. Related to this, most studies a priori selected discrete outcomes to analyze, thereby potentially ignoring other important variables that were not selected for analysis. Additionally, these discrete outcomes are often considered in isolation and future studies should investigate if a combination of multiple biomechanics improves relations with running economy (e.g., [172]).

Fifth, some studies did not report all information required for meta-analysis and we therefore extracted the required information from figures or estimated the information based on other studies. This likely introduced some error and we therefore urge researchers to improve reporting and provide open data. In line with these suggestions, we have provided all data extracted or provided by authors in the ESM to facilitate further research. Related to this, some studies did not report whether they verified a steady-state oxygen consumption to ensure a submaximal intensity. We excluded some studies (e.g., [100]) or specific speeds within other studies (e.g., 6 m∙s^−1^ in [108] or 5.83 m∙s^−1^ in [85]) as these were very likely not at a metabolic steady-state. While for some other included studies/speeds it is unknown if they were performed at a metabolic steady state, we included these studies as they typically used speeds at which similar populations were shown to run at a metabolic steady-state in other studies (e.g., [87, 116, 117, 119, 124]), or because the speed of the RE assessment was slower than the half-marathon speed (5.56 vs 5.86 m∙s^−1^, respectively) [92], thus likely being at a metabolic steady-state, in particular on the treadmill due to the smaller influence of air drag.

Sixth, some meta-analyses were affected by high levels of heterogeneity and a small number of studies. Although we attempted to explore the causes of the heterogeneity by performing meta-regressions, other factors that were not investigated such as running experience and competition distance may also have contributed to the heterogeneity. Related to this, optimal running biomechanics may be discipline-specific, with the technique for middle-distance athletes (e.g., 1500 m) being different from long-distance (e.g., marathon), although both types of trained athletes are likely more economical than untrained individuals [36]. Since individuals included in each study may have specialized in different distances, this could have confounded the relationship between running biomechanics and RE (e.g., [173, 174]). However, we could not include these factors as a subgroup or in meta-regression because most studies did not clearly specify the participant characteristics (Fig. 2). Similarly, although we attempted to explore the influence of anthropometric characteristics on the obtained correlations, there are numerous anthropometrical factors such as leg length [18] and calcaneus length [175] that may also contribute to differences in the most economical running biomechanics across individuals. It is therefore likely some components of running technique may be unique to a specific individual given their anthropometric characteristics.

Seventh, 15 studies (29.4%) standardized shoe wear while 26 (70.6%) studies did not standardize shoe wear. Non-standardization of shoe wear may introduce bias when associating RE with running biomechanics. For example, while most studies were conducted before the introduction of ‘super shoes’ with carbon plates and special cushioning in 2017, ‘traditional’ racing flat shoes can still enhance RE by ~ 2% as compared with ‘normal’ training shoes [176]. Similarly, differences in aspects such as heel-toe drop and midsole material properties may alter running biomechanics and thus confound the relation between running biomechanics and RE. In partial support of the relevance of shoe standardization, meta-regression showed stronger correlations between vertical displacement and RE when shoe wear was standardized (Supplementary File S5, see ESM).

Finally, 29 studies (58%) expressed RE as the mass specific rate of oxygen required to run at a given speed, while 16 (32%) studies determined the mass specific rate of energy required to run at a given speed (Table 1). Our sensitivity analysis showed that the differences in correlations between running biomechanics and RE expressed as the oxygen or energetic cost were generally small (< 0.03 units) (Supplementary File S3, see ESM), suggesting this had only a trivial impact on our findings. Nevertheless, we urge caution with the use of oxygen cost as it may confound correlations at higher relative speeds (see e.g., Fig. 5 oxygen vs energy cost correlation with change in speed). Related to this, the energetic cost of running can be determined using assumptions about the energy equivalent of oxygen, or equations that each assume different stoichiometry of the substrate used. Our sensitivity analysis showed that the equation used to determine energy cost had a negligible impact on the correlation with running biomechanics (mean difference in correlation of 0.01; Supplementary File S3, see ESM).

## Conclusion

Our findings show that among spatiotemporal outcomes, ground contact time, flight time, and duty factor showed trivial and non-significant associations with RE, while a higher stride frequency showed a small significant association with a better RE. Lower vertical oscillation and higher vertical and leg stiffness showed small to moderate magnitude correlations with better RE, while joint angles at specific instances of the gait cycle, joint angle range of motion, electromyographic muscle activation, and peak vertical ground reaction forces showed non-significant and often trivial associations with RE. Nevertheless, there were some trends (e.g., peak knee flexion angle, hip range of motion, and vastus lateralis/rectus femoris activation) worth exploring in future studies. Overall, our findings show that biomechanical variables can explain 4–12% of the between-individual variance in running economy when considered in isolation, with this magnitude potentially increasing when combining different variables. Moreover, we also show that some biomechanical variables often considered relevant to RE (e.g., contact time) are not overall associated with RE when assessed at a similar speed for all runners. While coaches, athletes, researchers, and developers of wearable technology may be tempted to use the biomechanical variables identified in this review to improve RE, further research is required to investigate if targeted training to modify these components is more effective than self-optimization. Finally, optimal performance may require optimization of running biomechanics beyond simply minimizing energy cost, thus suggesting that components not significantly associated with RE may still be relevant from a performance or injury preventative perspective.

## Supplementary Information

Below is the link to the electronic supplementary material.

Supplementary file1 (DOCX 20 kb)

Supplementary file2 (DOCX 28 kb)

Supplementary file3 (DOCX 38 kb)

Supplementary file4 (DOCX 45 kb)

Supplementary file5 (DOCX 64 kb)

Supplementary file6 (DOCX 2401 kb)
