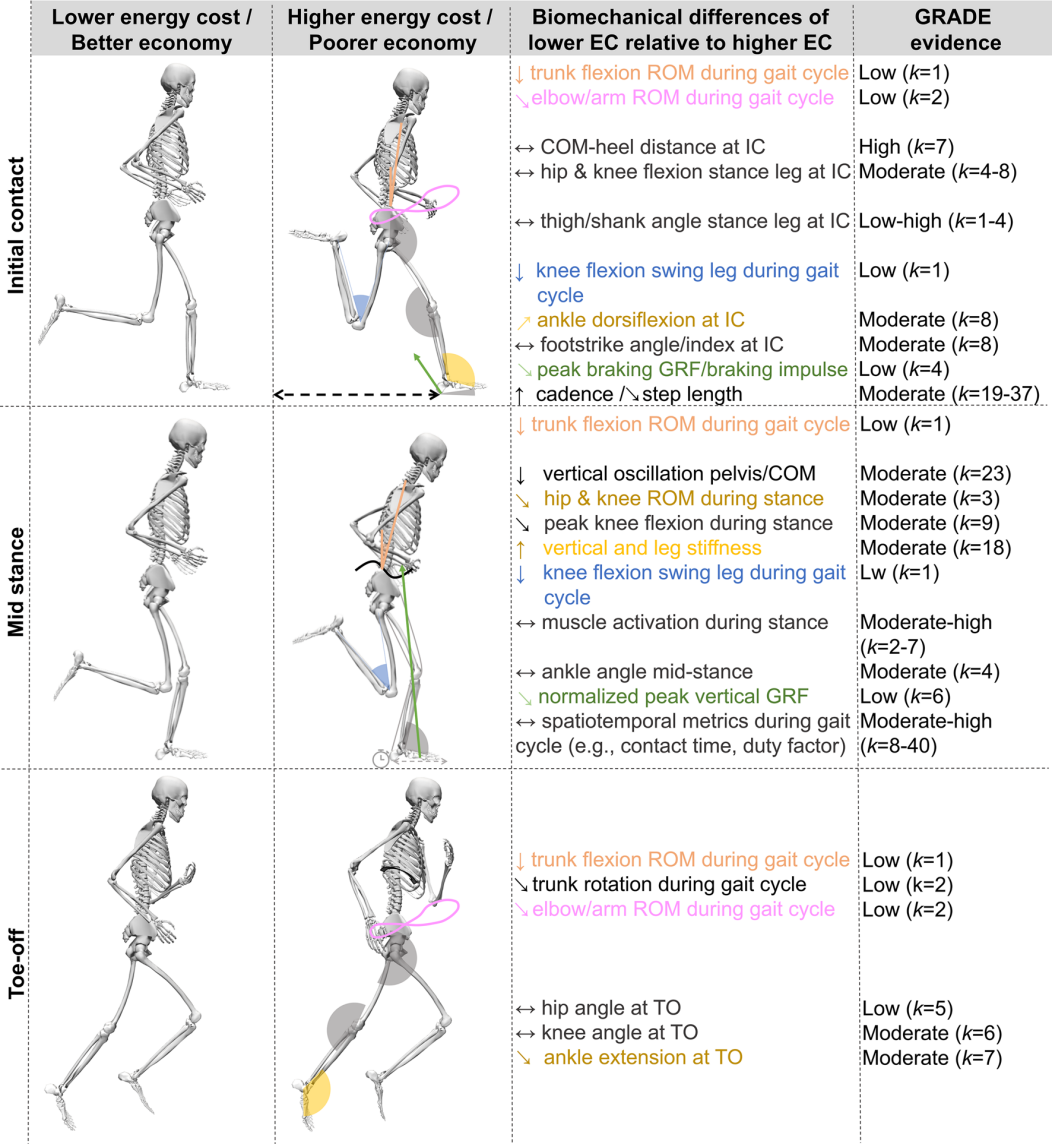# Correction to: The Relationship Between Running Biomechanics and Running Economy: A Systematic Review and Meta-Analysis of Observational Studies

**DOI:** 10.1007/s40279-024-02157-3

**Published:** 2025-01-17

**Authors:** Bas Van Hooren, Ivan Jukic, Maartje Cox, Koen G. Frenken, Iker Bautista, Isabel S. Moore

**Affiliations:** 1 Department of Nutrition and Movement Sciences, NUTRIM School of Nutrition and Translational Research in Metabolism, Maastricht University Medical Centre+, Universiteitssingel 50, 6229 ER Maastricht, The Netherlands; 2 Sport Performance Research Institute New Zealand (SPRINZ), Auckland University of Technology, Auckland, New Zealand; 3 School of Engineering, Computer and Mathematical Sciences, Auckland University of Technology, Auckland, New Zealand; 4 Institute of Sport, Nursing and Allied Health, University of Chichester, Chichester, UK; 5 Department of Physiotherapy, Catholic University of Valencia, Valencia, Spain; 6 School of Sport and Health Sciences, Cardif Metropolitan University, Cardif, UK

**Correction to: Sports Medicine (2024) 54:1269–1316** 10.1007/s40279-024-01997-3

In this article, Fig. 8 contained two minor errors, which have been corrected in the new figure shown below.

**Figure:**