# Supplementary material for: The Relationship Between Running Biomechanics and Running Economy: A Systematic Review and Meta-Analysis of Observational Studies
Source: Sports Med. 2024 Mar 6;54(5):1269–316. doi: 10.1007/s40279-024-01997-3 (PMC11127892; doi:10.1007/s40279-024-01997-3)
Supplement: Supplementary file 1 — Supplementary file1 (DOCX 20 kb) [file 40279_2024_1997_MOESM1_ESM.docx]

**Supplementary file S1. Search string for each database**

**PICOT**

- AND

|  | **Population** | **Test** | **Comparator** | **Outcome** | **Type** |
| --- | --- | --- | --- | --- | --- |
| 🡨 OR | Healthy Adults  Age 18-65 | - | - | Econom*  OR  Efficiency  OR  Oxygen  OR  Energy  OR  VO2  OR  Cost Of running  OR  Endurance  OR  Steady state  OR  Work  OR  Performance  **AND**  Technique  OR  Form  OR  Style  OR  Foot Strike  OR  Stride  OR Impact  OR  Kinematic*  OR  Kinetic*  OR  Biomechanic*  OR  Muscle activity  OR  electromyographic activity  OR  Muscle-tendon | Run* |

**Search Strategies:**

[tiab] = look only in title or abstract

* = truncation = words that have different endings are also included

-----------------------------------------------------------------------------------

**Most extensive search PubMed**

(“Running”[Mesh] OR run[title] OR running[title]) AND ("Oxygen Consumption"[MeSH] OR Economy[title] OR economical[title] OR Efficiency[title] OR Oxygen[title] OR Energy[title] OR VO2[title] OR “Cost Of running”[title] OR Endurance[title] OR “Steady state”[title] OR Work[title] OR Performance[title] OR “metabolic cost”[title]) AND (“Biomechanical Phenomena”[Mesh] OR Technique[tiab] OR Form[tiab] OR Style[tiab] OR “Foot Strike”[tiab] OR footstrike[tiab] OR Stride[tiab] OR cadence[tiab] OR "vertical oscillation"[tiab] OR "leg stiffness"[tiab] OR "contact time"[tiab] OR Impact[tiab] OR "vertical force"[tiab] OR "propulsive force"[tiab] OR Kinematic*[tiab] OR Kinetic*[tiab] OR Biomechanic*[tiab] OR spatiotemporal[tiab] OR "spatio-temporal"[tiab] OR “Muscle activation”[tiab] OR “Muscle activity”[tiab] OR electromyographic activ*[tiab] OR Muscle-tendon[tiab])

**OVID MEDLINE/OVID EMBASE**

(exp Running/ OR run.ti. OR running.ti.) AND (exp Oxygen Consumption/ OR Economy.ti. OR economical.ti. OR Efficiency.ti. OR Oxygen.ti. OR Energy.ti. OR VO2.ti. OR Cost Of running.ti. OR Endurance.ti. OR Steady state.ti. OR Work.ti. OR Performance.ti. OR metabolic cost.ti.) AND (exp Biomechanical Phenomena/ OR Technique.ti,ab. OR Form.ti,ab. OR Style.ti,ab. OR Foot Strike.ti,ab. OR footstrike.ti,ab. OR Stride.ti,ab. OR cadence.ti,ab. OR vertical oscillation.ti,ab. OR leg stiffness.ti,ab. OR contact time.ti,ab. OR Impact.ti,ab. OR vertical force.ti,ab. OR propulsive force.ti,ab. OR Kinematic*.ti,ab. OR Kinetic*.ti,ab. OR Biomechanic*.ti,ab. OR spatiotemporal.ti,ab. OR spatio-temporal.ti,ab. OR Muscle activation.ti,ab. OR Muscle activity.ti,ab. OR electromyographic activ*.ti,ab. OR Muscle-tendon.ti,ab.)

**EMBASE (title only)**

(Running OR run) AND ("Oxygen Consumption" OR Economy OR economical OR Efficiency OR Oxygen OR Energy OR VO2 OR "Cost Of running" OR Endurance OR "Steady state" OR Work OR Performance OR "metabolic cost") AND ("Biomechanical Phenomena" OR Technique OR Form OR Style OR "Foot Strike" OR footstrike OR Stride OR cadence OR "vertical oscillation" OR "leg stiffness" OR "contact time" OR Impact OR "vertical force" OR "propulsive force" OR Kinematic* OR Kinetic* OR Biomechanic* OR spatiotemporal OR spatio-temporal OR "Muscle activation" OR "Muscle activity" OR "electromyographic activ*" OR Muscle-tendon)

**Web of Science (title only)**

(Running OR run) AND ("Oxygen Consumption" OR Economy OR economical OR Efficiency OR Oxygen OR Energy OR VO2 OR "Cost Of running" OR Endurance OR "Steady state" OR Work OR Performance OR "metabolic cost") AND ("Biomechanical Phenomena" OR Technique OR Form OR Style OR "Foot Strike" OR footstrike OR Stride OR cadence OR "vertical oscillation" OR "leg stiffness" OR "contact time" OR Impact OR "vertical force" OR "propulsive force" OR Kinematic* OR Kinetic* OR Biomechanic* OR spatiotemporal OR spatio-temporal OR "Muscle activation" OR "Muscle activity" OR "electromyographic activ*" OR Muscle-tendon)

**SportRxiv (11 hits, all irrelevant)**

(Running OR run) AND ("Oxygen Consumption" OR Economy OR economical OR Efficiency OR Oxygen OR Energy OR VO2 OR "Cost Of running" OR Endurance OR "Steady state" OR Work OR Performance OR "metabolic cost") AND ("Biomechanical Phenomena" OR Technique OR Form OR Style OR "Foot Strike" OR footstrike OR Stride OR cadence OR "vertical oscillation" OR "leg stiffness" OR "contact time" OR Impact OR "vertical force" OR "propulsive force" OR Kinematic* OR Kinetic* OR Biomechanic* OR spatiotemporal OR spatio-temporal OR "Muscle activation" OR "Muscle activity" OR "electromyographic activ*" OR Muscle-tendon)

**BioRxiv (3 hits, all irrelevant)**

(“Running”[Mesh] OR run[title] OR running[title]) AND ("Oxygen Consumption"[MeSH] OR Economy[title] OR economical[title] OR Efficiency[title] OR Oxygen[title] OR Energy[title] OR VO2[title] OR “Cost Of running”[title] OR Endurance[title] OR “Steady state”[title] OR Work[title] OR Performance[title] OR “metabolic cost”[title]) AND (“Biomechanical Phenomena”[Mesh] OR Technique[tiab] OR Form[tiab] OR Style[tiab] OR “Foot Strike”[tiab] OR footstrike[tiab] OR Stride[tiab] OR cadence[tiab] OR "vertical oscillation"[tiab] OR "leg stiffness"[tiab] OR "contact time"[tiab] OR Impact[tiab] OR "vertical force"[tiab] OR "propulsive force"[tiab] OR Kinematic*[tiab] OR Kinetic*[tiab] OR Biomechanic*[tiab] OR spatiotemporal[tiab] OR "spatio-temporal"[tiab] OR “Muscle activation”[tiab] OR “Muscle activity”[tiab] OR electromyographic activ*[tiab] OR Muscle-tendon[tiab])
