# Supplementary material for: The Relationship Between Running Biomechanics and Running Economy: A Systematic Review and Meta-Analysis of Observational Studies
Source: Sports Med. 2024 Mar 6;54(5):1269–316. doi: 10.1007/s40279-024-01997-3 (PMC11127892; doi:10.1007/s40279-024-01997-3)
Supplement: Supplementary file 2 — Supplementary file2 (DOCX 28 kb) [file 40279_2024_1997_MOESM2_ESM.docx]

**Supplementary file S2 Risk of bias assessment questions and rating criteria**

| **Assessment criterion** | **Risk of bias rating** | **Additional rating information** |
| --- | --- | --- |
| 1. Were the inclusion/exclusion criteria clearly described and justified? | **Yes** (low risk of bias): The study clearly described all inclusion/exclusion criteria (see ‘additional rating information’).  **Reasonably** (moderate risk of bias): The study clearly described all but one of the inclusion/exclusion criteria.  **No** (high risk of bias): The study did not describe the inclusion/exclusion criteria, or the description did not specify two or more criteria. | Criteria: sex, age cut-off(s) performance/training background cut-offs, or injury. |
| 2. Were the participants’ characteristics described with a sufficient detail? | **Yes** (low risk of bias): The participants’ characteristics were clearly described with sufficient detail.  **Reasonably** (moderate risk of bias): Some participants’ characteristics were described with sufficient detail (see additional rating information column for more details).  **No** (high risk of bias): The participants’ characteristics were not described with sufficient detail hence not allowing the determination of the study’s population of interest. | If more than one or two of these characteristics were missing, this question was rated as moderate or high risk of bias, respectively: sex, age, height, body mass, ethnicity/nationality, and running ability (i.e., subjective description of range included; personal best times; ${\dot{V}O}_{2}$_max_, running economy values per speed; running experience). |
| 3. Were data collected representative of a direct measure (as opposed to a proxy)? | **Yes** (low risk of bias): Data collected for all variables are representative of a direct measure of a given outcome.  **Reasonably** (moderate risk of bias): Data collected for all but one variable are representative of a direct measure of a given outcome.  **No** (high risk of bias): Data collected for the vast majority of variables are representative of a proxy measure of a given outcome. | If running economy was measured only as ${\dot{V}O}_{2}$ rather than energy cost (in Joules or Kcal), this was considered as at least moderate risk of bias [1]. Additionally, modelling of leg stiffness through spatiotemporal variables and estimation of joint angles from spatiotemporal variables (e.g., stride angle) were considered at least moderate risk of bias. Similarly, centre of mass estimated derived from e.g., the ground reaction force were considered to be moderate risk of bias as opposed to estimated derived from full-body kinematics. |
| 4. Were the study instruments (or procedures) that measured the parameters of interest shown to be valid and reliable? | **Yes** (low risk of bias): All study instruments (or procedures) used to measure the parameters of interest were previously shown to be valid and reliable for measuring the outcomes of interest or the authors established the study instruments’ reliability and validity as a part of the study.  **Reasonably** (moderate risk of bias): All but one instruments (or procedures) used to measure the parameters of interest were previously shown to be valid and reliable for measuring the outcomes of interest or the authors established the study instruments’ reliability and validity as a part of the study.  **No** (high risk of bias): The study instruments (or procedures) used to measure the parameters of interest were not previously showed to be valid and reliable and the authors did not establish the study instruments’ reliability and validity as a part of the study. | If 3D motion capture was used with sufficient sampling frequency (≥200 Hz), all kinematic outcomes were assumed to be of low risk of bias. Similarly, if forces were samples with sufficient frequency (≥1000 Hz), all kinetic outcomes were assumed to be of low risk of bias. Accelerometer-based estimates were considered to be at least of moderate risk of bias when their outcomes were not validated due to the potential effect of noise on the obtained biomechanical outcomes. |
| 5. Was the assessment of the parameters of interest performed using the same technical and environmental conditions? | **Yes** (low risk of bias): All technical and environmental conditions were controlled for and were identical during all testing procedures for all participants.  **Reasonably** (moderate risk of bias): All but one technical and environmental conditions were controlled for and were identical during all testing procedures for all participants.  **No** (high risk of bias): The technical and environmental conditions were not controlled and standardized for all participants. | This question was rated as no when more than two of the following criteria were met: 1) the study used outdoor measurements where wind and temperature effects were not controlled (unless the authors explicitly mention that tests were performed only in specific conditions); 2) shoe wear was not standardized between participants; 3) the study did not detail how overground running speed was controlled; 4) measurements were performed at different times of the day for each participant; 5) authors did not describe how steady-state was determined.  If running economy and running biomechanics were assessed in different conditions (e.g. running economy on a treadmill, running biomechanics overground), we considered this to be at least moderate risk of bias since differences in surface and running speed could have influenced running biomechanics and running economy. In contrast, if running economy and running biomechanics were assessed simultaneously, we considered this to be of low risk of bias since metabolic testing has been shown to have no substantial influence on lower body sagittal plane kinematics and running economy [2, 3].  The use of non-standardized running shoes was considered at least moderate risk of bias since different running shoes have been shown to substantially influence running economy, particularly when only a short (e.g., 5 min) familiarization duration is provided [4]. |
| 6. Were the important/potential confounding factors clearly described and accounted for? | **Yes** (low risk of bias): All known and important confounding factors were clearly described and the attempt was made to control for them with the analyses, or their potential effects on the results were thoroughly discussed in the manuscript and clearly reported in the limitations section of the manuscript.  **Reasonably** (moderate risk of bias): All but one important confounding factors were clearly described and the attempt was made to control for them with the analyses, or their potential effects on the results were thoroughly discussed in the manuscript and clearly reported in the limitations section of the manuscript.  **No** (high risk of bias): All known and important confounding factors were not clearly described, the attempt to control for them with the analyses was not made, and their potential effects on the results were not discussed in the manuscript or reported in the limitations section of the manuscript. |  |
| 7. Was the appropriate statistical test used for the main analysis of interest and were the assumptions behind this test met and clearly described? | **Yes** (low risk of bias): The study reported and confirmed that all the assumptions behind the statistical test (e.g. Pearson’s *r*) were met or that the appropriate alternative test (e.g. Spearman’s *ρ*) was used when this was not the case.  **Reasonably** (moderate risk of bias): The study did not report and confirm the assumptions behind the main test.  **No** (high risk of bias): The study did not use the appropriate statistical test for the main analysis of interest (e.g. independent t-test on dependent sample). |  |
| 8. Summary item on the overall risk of study bias. | **Low risk of bias:** Further research is very unlikely to change our confidence in the estimate.  **Moderate risk of bias:** Further research is likely to change our confidence in the estimate.  **High risk of bias:** Further research is very likely to have an important impact on our confidence in the estimate and may change the estimate. |  |

**References**

1. Shaw AJ, Ingham SA, Folland JP. The valid measurement of running economy in runners. Med Sci Sports Exerc. 2014;46(10):1968-73. doi:10.1249/MSS.0000000000000311.

2. Sloan RS, Wight JT, Hooper DR, Garman JE, Pujalte GG. Metabolic testing does not alter distance running lower body sagittal kinematics. Gait Posture. 2020;76:403-8.

3. Siler WL. Is running style and economy affected by wearing respiratory apparatus? Med Sci Sports Exerc. 1993;25(2):260-4.

4. Hoogkamer W, Kipp S, Frank JH, Farina EM, Luo G, Kram R. A Comparison of the Energetic Cost of Running in Marathon Racing Shoes. Sports Med. 2018;48(4):1009-19. doi:10.1007/s40279-017-0811-2.
