# Supplementary material for: The Relationship Between Running Biomechanics and Running Economy: A Systematic Review and Meta-Analysis of Observational Studies
Source: Sports Med. 2024 Mar 6;54(5):1269–316. doi: 10.1007/s40279-024-01997-3 (PMC11127892; doi:10.1007/s40279-024-01997-3)
Supplement: Supplementary file 3 — Supplementary file3 (DOCX 38 kb) [file 40279_2024_1997_MOESM3_ESM.docx]

**Supplementary file S3 Sensitivity analysis**

1. **Sensitivity analysis on oxygen vs energy cost**

Some studies reported separate correlations between running biomechanics and running economy expressed as either oxygen or energy cost [1], or provided individual participant data or meta-data that allowed computation of separate correlations [2-9]. For the most common outcomes (i.e., contact time and step frequency) reported in the respective study, we computed the difference in the correlation coefficient when running economy was expressed as oxygen cost or energy cost (using the stochiometric equation used by the authors) and subsequently computed the mean difference overall studies for each outcome.

This analysis revealed that the contact time and step frequency correlation coefficients were higher and lower by 0.03 and 0.01 units when running economy was expressed as the energy cost instead of the oxygen cost, respectively (Table S1).

**Table S1. Correlation coefficients with running economy expressed as oxygen or energy cost**

|  |  | **Contact time** | | |  | **Cadence** | | | |
| --- | --- | --- | --- | --- | --- | --- | --- | --- | --- |
| **Study** | **Speed (m∙s^-1^)** | **Corr. with OC** | **Corr. With EC** | **Difference** |  | | **Corr. with OC** | **Corr. With EC** | **Difference** |
| Lemire et al. (2021) | 2.22 | -0.46 | -0.47 | **0.01** |  | | -0.01 | -0.02 | **0.02** |
| Lemire et al. (2021) | 2.78 | -0.35 | -0.32 | **-0.03** |  | | -0.05 | -0.10 | **0.05** |
| Lemire et al. (2021) | 3.33 | 0.12 | 0.16 | **-0.05** |  | | -0.22 | -0.22 | **0.00** |
| Lemire et al. (2021) | 3.89 | -0.47 | -0.38 | **-0.09** |  | | 0.08 | 0.03 | **0.05** |
| Lussiana et al. (2017) | 3.33 | 0.11 | 0.21 | **-0.10** |  | |  |  | **0.00** |
| Howe et al. (2021) | 2.22 |  |  |  |  | | -0.05 | -0.06 |  |
| Pastor et al. (2022) | 2.78 | 0.00 | 0.03 | **-0.03** |  | | 0.12 | 0.09 | **0.03** |
| Pastor et al. (2022) | 3.89 | -0.11 | -0.09 | **-0.02** |  | | 0.29 | 0.22 | **0.07** |
| Seki et al. (2020) | 3.33 | -0.45 | -0.54 | **0.08** |  | | -0.64 | -0.41 | **-0.23** |
| Lussiana et al. (2019) | 2.78 | -0.14 | -0.14 | **0.00** |  | | -0.17 | -0.17 | **0.00** |
| Lussiana et al. (2019) | 3.33 | 0.05 | 0.05 | **0.00** |  | | -0.09 | -0.09 | **0.00** |
| Lussiana et al. (2019) | 3.89 | 0.11 | 0.11 | **0.01** |  | | -0.02 | -0.02 | **0.00** |
| Beck et al. (2020) | 3.5 | 0.51 | 0.59 | **-0.08** |  | | 0.04 | -0.05 | **0.09** |
| **Mean** |  |  |  | **-0.03** |  | |  |  | **0.01** |

EC = energy cost; OC = oxygen cost.

1. **Sensitivity analysis on different** **stoichiometric equations for energy cost**

For three studies (with one study providing data at three different speeds) that provided individual participant data for VO_2_ and VCO_2_, we also explored the effect of different stochiometric equations on the resulting correlation coefficients for contact time and step frequency. We computed the correlation coefficients using the Jeukendrup equation for moderate intensity exercise [10] and the Péronnet equation [11]. The difference in correlation coefficients was again computed as detailed above.

This analysis revealed that the correlation coefficients differed by 0.01 units for contact time and cadence when energy cost was calculated using the Jeukendrup or Péronnet equation (Table S2).

**Table S2. Correlation coefficients with energy cost calculated by the Jeukendrup or the Péronnet equation**

|  |  | **Contact time** | | |  | **Cadence** | | |
| --- | --- | --- | --- | --- | --- | --- | --- | --- |
| **Study** | **Speed (m∙s^-1^)** | **Corr. with Jeukendrup** | **Corr. With Péronnet** | **Difference** |  | **Corr. with Jeukendrup** | **Corr. With Péronnet** | **Difference** |
| Beck et al. (2020) | 3.5 | 0.55 | 0.59 | **0.04** |  | -0.00 | -0.05 | **0.05** |
| Lussiana et al. (2019) | 2.78 | 0.21 | 0.21 | **0.00** |  | -0.26 | -0.26 | **0.00** |
| Lussiana et al. (2019) | 3.33 | 0.24 | 0.24 | **0.00** |  | -0.25 | -0.25 | **0.00** |
| Lussiana et al. (2019) | 3.89 | 0.25 | 0.25 | **0.00** |  | -0.25 | -0.26 | **0.00** |
| Lussiana et al. (2017) | 3.33 | 0.10 | 0.11 | **0.01** |  |  |  |  |
| **Mean** |  |  |  | **0.01** |  |  |  | **0.01** |

1. **References**

1. Lundby C, Montero D, Gehrig S, Andersson Hall U, Kaiser P, Boushel R et al. Physiological, biochemical, anthropometric, and biomechanical influences on exercise economy in humans. Scand J Med Sci Sports. 2017;27(12):1627-37. doi:10.1111/sms.12849.

2. Lemire M, Falbriard M, Aminian K, Millet GP, Meyer F. Level, Uphill, and Downhill Running Economy Values Are Correlated Except on Steep Slopes. Front Physiol. 2021;12:697315. doi:10.3389/fphys.2021.697315.

3. Seki K, Kyrolainen H, Sugimoto K, Enomoto Y. Biomechanical factors affecting energy cost during running utilising different slopes. J Sports Sci. 2020;38(1):6-12. doi:10.1080/02640414.2019.1676527.

4. Pastor FS, Besson T, Berthet M, Varesco G, Kennouche D, Dandrieux P-E et al. Elite Road vs. Trail Runners: Comparing Economy, Biomechanics, Strength, and Power. J Strength Cond Res. 2022.

5. Lussiana T, Gindre C, Hebert-Losier K, Sagawa Y, Gimenez P, Mourot L. Similar Running Economy With Different Running Patterns Along the Aerial-Terrestrial Continuum. Int J Sports Physiol Perform. 2017;12(4):481-9. doi:10.1123/ijspp.2016-0107.

6. Lussiana T, Patoz A, Gindre C, Mourot L, Hebert-Losier K. The implications of time on the ground on running economy: less is not always better. J Exp Biol. 2019;222(Pt 6):jeb192047. doi:10.1242/jeb.192047.

7. Vercruyssen F, Tartaruga M, Horvais N, Brisswalter J. Effects of Footwear and Fatigue on Running Economy and Biomechanics in Trail Runners. Med Sci Sports Exerc. 2016;48(10):1976-84. doi:10.1249/MSS.0000000000000981.

8. Beck ON, Golyski PR, Sawicki GS. Adding carbon fiber to shoe soles may not improve running economy: a muscle-level explanation. Sci Rep. 2020;10(1):1-13.

9. Howe CCF, Swann N, Spendiff O, Kosciuk A, Pummell EKL, Moir HJ. Performance determinants, running energetics and spatiotemporal gait parameters during a treadmill ultramarathon. Eur J Appl Physiol. 2021;121(6):1759-71. doi:10.1007/s00421-021-04643-2.

10. Jeukendrup AE, Wallis GA. Measurement of substrate oxidation during exercise by means of gas exchange measurements. Int J Sports Med. 2005;26 Suppl 1(S 1):S28-37. doi:10.1055/s-2004-830512.

11. Peronnet F, Massicotte D. Table of nonprotein respiratory quotient: an update. Can J Sport Sci. 1991;16(1):23-9.
