# Supplementary material for: The Relationship Between Running Biomechanics and Running Economy: A Systematic Review and Meta-Analysis of Observational Studies
Source: Sports Med. 2024 Mar 6;54(5):1269–316. doi: 10.1007/s40279-024-01997-3 (PMC11127892; doi:10.1007/s40279-024-01997-3)
Supplement: Supplementary file 4 — Supplementary file4 (DOCX 45 kb) [file 40279_2024_1997_MOESM4_ESM.docx]

**Online supplementary file S4 Data reduction**

1. **Data reduction**

Folland et al. [1] reported the average correlations between running biomechanics and running economy across three speeds and we therefore used the average of the three speeds as running speed in meta-regressions. For all but one outcome, data was measured among 97 individuals, with 90 individuals being measured for centre of mass vertical displacement. Moreover, both vertical displacement and centre of mass were measured during only the stance phase and during a complete stride, thus providing four correlations for one meta-analysis from this study. Because we combined stance and stride vertical displacement of the pelvis, and vertical displacement of the centre of mass in one meta-analysis, we used the average number of participants across both outcomes (90+97)/2 = 93.5 to allocate participants to all four outcomes (93.5/4 = 23.4), resulting in 23 for each outcome with one outcome using 24 participants. This ensured that the study was weighted similar to the total sample size, but also that all four outcomes could be included in the analysis.

Howe and colleagues [2] investigated changes in running biomechanics following a fatiguing marathon but provided meta-data to determine a Pearson correlation coefficient between running biomechanics and energy cost prior to the ultramarathon to be used for the review.

Bourdin et al. [3] reported correlations between running biomechanics and running economy during loaded and unloaded running and we used only the unloaded condition for analyses.

Tartaruga et al. [4] reported correlations between running biomechanics and running economy, with running economy expressed as allometrically or ratio scaled for body mass. We used only the ratio scaled values to be consistent with how most other studies expressed running economy (as can be seen in Table I).

Bohm et al. [5] investigated the effect of plantar flexor strength training on running economy and also reported running biomechanics. We used the pre-intervention data from the intervention group to compute Pearson correlation coefficients between running biomechanics and running economy, which we included in the analyses.

Craighead et al. [6] investigated the effect of gait retraining on running economy and provided the individual participant data from the pre- and post-test. We used the data from the pre-test to compute correlations between the measured running biomechanical outcomes and running economy for inclusion in this review.

Willis et al. [7] investigated correlations between running economy and running biomechanics in uphill and level running but provided separate correlations for males and females (as they ran at different speeds) between running economy and biomechanics for the level condition, which we included in the analyses.

Joubert, Jones [8] investigated differences in running economy and running biomechanics between different shoes but provided the individual running economy and biomechanical data for the control shoe averaged across two trials, which we used to compute Pearson correlations for meta-analyses.

Joubert et al. [9] also provided the correlation between running biomechanics and running economy averaged across shoes for use in meta-analyses.

Similarly, Hoogkamer et al. [10] provided running economy and biomechanical meta-data for different shoes, which we used to compute an average correlation between running biomechanics and running economy across all shoes for use in meta-analyses.

Beck and colleagues [11] compared running biomechanics and running economy between different shoes and provided meta-data, which we used to compute average running biomechanics and running economy values over all shoes, which in turn were used to compute correlations for meta-analyses. Moreover, the footstrike pattern and running economy data were used to perform an independent *t*-test, which in turn could be used in the meta-analysis on footstrike and running economy.

Barnes et al. [12] measured 39 males and 24 females and we used the sex-specific correlations for analysis instead of aggregated correlations.

Pastor and colleagues [13] compared running economy and running biomechanics between elite road and trail runners and provided combined correlations across groups between biomechanical outcomes and both O_2_ and energy cost during level running at 10 and 14 km/h. We used the energy cost correlations provided for meta-analyses.

Seki et al. [14] provided correlations between running economy and biomechanical outcomes during the level-only running condition, which were included in meta-analyses.

Lussiana et al. [15] provided the meta-data from which we derived correlations between the biomechanical outcomes and running economy expressed as the energetic cost. Moreover, since the dataset contained footstrike angles, runners were classified into a rearfoot or mid/front foot runner based on the footstrike angle using criteria described by Altman and Davis [16] (i.e. >8 was considered rearfoot strikers, below 8 as mid/forefoot striker) and this data was used in the between-group meta-analysis on footstrike and running economy. Moreover, muscle activation was reported for 2 subphases of the contact and flight phases. We computed an average correlation over the two phases to allow combination with other studies that only reported muscle activation over the whole contact of the flight phase.

Meta-data were also provided by Lussiana et al. [17], which we used to compute correlations between running economy expressed as the energetic cost and biomechanical outcomes.

Vercruyssen et al. [18] provided meta-data with biomechanical data and running economy pre-fatigue. We used the running economy expressed as caloric cost and data from the traditional shoe to compute correlations for meta-analyses. The provided footstrike pattern and running economy data were used to perform an independent *t*-test, which in turn could be used in the meta-analysis on footstrike and running economy.

Rogers et al. [19] provided individual data on running economy and contact and flight times. We computed step time and duty factor from these outcomes and used all outcomes in meta-analyses.

Ogueta-Alday et al. [20] provided running economy data for the rearfoot and midfoot strikers, which were used to compute *p*-values for the between-group difference.

Two studies reported correlations between swing time and running economy [21, 22], but the swing time values were in line with flight time values reported in other papers. Therefore, these outcomes were re-classified as flight time for further analysis.

1. **References**

1. Folland JP, Allen SJ, Black MI, Handsaker JC, Forrester SE. Running Technique is an Important Component of Running Economy and Performance. Med Sci Sports Exerc. 2017;49(7):1412-23. doi:10.1249/MSS.0000000000001245.

2. Howe CCF, Swann N, Spendiff O, Kosciuk A, Pummell EKL, Moir HJ. Performance determinants, running energetics and spatiotemporal gait parameters during a treadmill ultramarathon. Eur J Appl Physiol. 2021;121(6):1759-71. doi:10.1007/s00421-021-04643-2.

3. Bourdin M, Belli A, Arsac LM, Bosco C, Lacour JR. Effect of vertical loading on energy cost and kinematics of running in trained male subjects. J Appl Physiol (1985). 1995;79(6):2078-85. doi:10.1152/jappl.1995.79.6.2078.

4. Tartaruga MP, Peyré-Tartaruga LA, Coertjens M, De Medeiros MH, Kruel LFM. The influence of the allometric scale on the relationship between running economy and biomechanical variables in distance runners. Biol Sport. 2009;26(3):263-73. doi:10.5604/20831862.894791.

5. Bohm S, Mersmann F, Santuz A, Arampatzis A. Enthalpy efficiency of the soleus muscle contributes to improvements in running economy. Proc Biol Sci. 2021;288(1943):20202784. doi:10.1098/rspb.2020.2784.

6. Craighead DH, Lehecka N, King DL. A novel running mechanic's class changes kinematics but not running economy. J Strength Cond Res. 2014;28(11):3137-45. doi:10.1519/JSC.0000000000000500.

7. Willis SJ, Gellaerts J, Mariani B, Basset P, Borrani F, Millet GP. Level Versus Uphill Economy and Mechanical Responses in Elite Ultratrail Runners. Int J Sports Physiol Perform. 2019;14(7):1001-5.

8. Joubert DP, Jones GP. A comparison of running economy across seven highly cushioned racing shoes with carbon-fibre plates. Footwear Science. 2022:1-13.

9. Joubert DP, Dominy T, Burns GT. Effects of a Highly Cushioned Racing Shoe on Running Economy at Slower Running Speeds. SportRxiv. 2022. doi:<https://doi.org/10.51224/SRXIV.141>.

10. Hoogkamer W, Kipp S, Frank JH, Farina EM, Luo G, Kram R. A Comparison of the Energetic Cost of Running in Marathon Racing Shoes. Sports Med. 2018;48(4):1009-19. doi:10.1007/s40279-017-0811-2.

11. Beck ON, Golyski PR, Sawicki GS. Adding carbon fiber to shoe soles may not improve running economy: a muscle-level explanation. Sci Rep. 2020;10(1):1-13.

12. Barnes KR, McGuigan MR, Kilding AE. Lower-body determinants of running economy in male and female distance runners. J Strength Cond Res. 2014;28(5):1289-97. doi:10.1519/JSC.0000000000000267.

13. Pastor FS, Besson T, Berthet M, Varesco G, Kennouche D, Dandrieux P-E et al. Elite Road vs. Trail Runners: Comparing Economy, Biomechanics, Strength, and Power. J Strength Cond Res. 2022.

14. Seki K, Kyrolainen H, Sugimoto K, Enomoto Y. Biomechanical factors affecting energy cost during running utilising different slopes. J Sports Sci. 2020;38(1):6-12. doi:10.1080/02640414.2019.1676527.

15. Lussiana T, Gindre C, Hebert-Losier K, Sagawa Y, Gimenez P, Mourot L. Similar Running Economy With Different Running Patterns Along the Aerial-Terrestrial Continuum. Int J Sports Physiol Perform. 2017;12(4):481-9. doi:10.1123/ijspp.2016-0107.

16. Altman AR, Davis IS. A kinematic method for footstrike pattern detection in barefoot and shod runners. Gait Posture. 2012;35(2):298-300. doi:10.1016/j.gaitpost.2011.09.104.

17. Lussiana T, Patoz A, Gindre C, Mourot L, Hebert-Losier K. The implications of time on the ground on running economy: less is not always better. J Exp Biol. 2019;222(Pt 6):jeb192047. doi:10.1242/jeb.192047.

18. Vercruyssen F, Tartaruga M, Horvais N, Brisswalter J. Effects of Footwear and Fatigue on Running Economy and Biomechanics in Trail Runners. Med Sci Sports Exerc. 2016;48(10):1976-84. doi:10.1249/MSS.0000000000000981.

19. Rogers SA, Whatman CS, Pearson SN, Kilding AE. Assessments of Mechanical Stiffness and Relationships to Performance Determinants in Middle-Distance Runners. Int J Sports Physiol Perform. 2017;12(10):1329-34. doi:10.1123/ijspp.2016-0594.

20. Ogueta-Alday A, Rodriguez-Marroyo JA, Garcia-Lopez J. Rearfoot striking runners are more economical than midfoot strikers. Med Sci Sports Exerc. 2014;46(3):580-5. doi:10.1249/MSS.0000000000000139.

21. Santos-Concejero J, Granados C, Irazusta J, Bidaurrazaga-Letona I, Zabala-Lili J, Tam N et al. Differences in ground contact time explain the less efficient running economy in north african runners. Biol Sport. 2013;30(3):181-7. doi:10.5604/20831862.1059170.

22. Santos-Concejero J, Olivan J, Mate-Munoz JL, Muniesa C, Montil M, Tucker R et al. Gait-cycle characteristics and running economy in elite Eritrean and European runners. Int J Sports Physiol Perform. 2015;10(3):381-7. doi:10.1123/ijspp.2014-0179.
