# Supplementary material for: The Relationship Between Running Biomechanics and Running Economy: A Systematic Review and Meta-Analysis of Observational Studies
Source: Sports Med. 2024 Mar 6;54(5):1269–316. doi: 10.1007/s40279-024-01997-3 (PMC11127892; doi:10.1007/s40279-024-01997-3)
Supplement: Supplementary file 5 — Supplementary file5 (DOCX 64 kb) [file 40279_2024_1997_MOESM5_ESM.docx]

**Supplementary file S5 Meta-regression results**

**Table S3. Meta-regression outcomes**

| **Outcome** | **Moderator** | **Studies *(n)*** | **Effect sizes *(k)*** | ***β* (95% CI) ^a^** | **Overall ^b^** | **Direction moderation** | **Interpretation** |
| --- | --- | --- | --- | --- | --- | --- | --- |
| **Spatiotemporal outcomes** |  |  |  |  |  |  |  |
| Contact time | **Model 1** |  |  |  |  |  |  |
|  | Intercept | 24 | 40 | -0.15 (-0.67 to 0.36) | F(4, 8.30) = 0.45, *p* = 0.77 |  |  |
|  | Standardized shoes (NS) | 24 | 40 | -0.09 (-0.36 to 0.19) |  | ↔ | Shoe stan. did not influence the corr. |
|  | RE units (OC) | 24 | 40 | 0.21 (-0.13 to 0.54) |  | ↔ | RE units did not influence the corr. |
|  | Speed | 24 | 40 | 0.01 (-0.13 to 0.16) |  | ↔ | Speed did not influence the corr. |
|  | Corrected for resting OC/EC | 24 | 40 | 0.07 (-0.27 to 0.41) |  | ↔ | Correcting did not influence the corr. |
|  | **Model 2** |  |  |  |  |  |  |
|  | Intercept | 24 | 40 | -0.01 (-0.47 to 0.45) | F(1, 6.02) = 0.00, *p* = 0.97 |  |  |
|  | CV of age | 24 | 40 | 0.00 (-0.02 to 0.02) |  | ↔ | CV of age did not influence the corr. |
|  | **Model 3** |  |  |  |  |  |  |
|  | Intercept | 24 | 40 | -0.13 (-0.52 to 0.26) | F(1, 1.71) = 1.01, *p* = 0.43 |  |  |
|  | CV of height | 24 | 40 | 0.03 (-0.12 to 0.18) |  | ↔ | CV of height did not influence the corr. |
|  | **Model 4** |  |  |  |  |  |  |
|  | Intercept | 24 | 40 | -0.24 (-1.07 to 0.59) | F(1, 4.29) = 0.34, *p* = 0.59 |  |  |
|  | CV of body mass | 24 | 40 | 0.02 (-0.07 to 0.11) |  | ↔ | CV of mass did not influence the corr. |
|  | **Model 5** |  |  |  |  |  |  |
|  | Intercept | 10 | 19 | -0.12 (-0.66 to 0.42) | F(1, 2.42) = 0.00, *p* = 0.98 |  |  |
|  | CV of VO_2_max | 10 | 19 | -0.00 (-0.09 to 0.09) |  | ↔ | CV of VO_2_max did not influence the corr. |
|  |  |  |  |  |  |  |  |
| Flight time | **Model 1** |  |  |  |  |  |  |
|  | Intercept | 8 | 18 | 0.87 (0.09 to 1.65) | F(1, 3.47) = 3.83, *p* = 0.13 |  |  |
|  | Speed | 8 | 18 | -0.24 (0.44 to -0.04) |  | ↔ | Speed did not influence the corr. |
|  | RE units (OC) | 8 | 18 | 0.34 (-0.27 to 0.95) |  | ↔ | RE units did not influence the corr. |
|  | **Model 2** |  |  |  |  |  |  |
|  | Intercept | 8 | 18 | 0.41 (-0.50 to 1.32) | F(1, 3.74) = 1.14, *p* = 0.35 |  |  |
|  | Speed | 8 | 18 | -0.08 (-0.31 to 0.14) |  | ↔ | Speed did not influence the corr. |
|  | **Model 3** |  |  |  |  |  |  |
|  | Intercept | 8 | 18 | 0.06 (-0.19 to 0.32) | F(1, 1.59) = 1.23, *p* = 0.40 |  |  |
|  | RE units (OC) | 8 | 18 | 0.23 (-093 to 1.40) |  | ↔ | RE units did not influence the corr. |
|  | **Model 4** |  |  |  |  |  |  |
|  | Intercept | 8 | 18 | -0.02 (-0.29 to 0.26) | F(1, 1.76) = 6.49, *p* = 0.14 |  |  |
|  | Corrected for resting OC/EC | 8 | 18 | 0.31 (-0.29 to 0.92) |  | ↔ | Correcting did not influence the corr. |
|  | **Model 5** |  |  |  |  |  |  |
|  | Intercept | 8 | 17 | -0.32 (-0.73 to 0.08) | F(1, 2.45) = 19.9, *p* = 0.03 |  |  |
|  | CV of age* | 8 | 17 | 0.02 (0.00 to 0.03) |  | ↑ | ↑ corr. with ↑ CV of age |
|  | **Model 6** |  |  |  |  |  |  |
|  | Intercept | 8 | 18 | 0.30 (-0.17 to 0.77) | F(1, 1.68) = 1.74, *p* = 0.34 |  |  |
|  | CV of height | 8 | 18 | -0.04 (-0.21 to 0.13) |  | ↔ | CV of height did not influence the corr. |
|  | **Model 7** |  |  |  |  |  |  |
|  | Intercept | 8 | 16 | -0.63 (-1.05 to -0.20) | F(1, 1.69) = 69.7, *p* = 0.02 |  |  |
|  | CV of body mass* | 8 | 16 | 0.06 (0.02 to 0.10) |  | ↑ | ↑ corr. with ↑ CV of body mass |
|  | **Model 8** |  |  |  |  |  |  |
|  | Intercept | 4 | 10 | 0.06 (-1.83 to 1.95) | F(1, 1.71) = 0.18, *p* = 0.72 |  |  |
|  | CV of VO_2_max | 4 | 10 | 0.01 (-0.18 to 0.21) |  | ↔ | CV of VO_2_max did not influence the corr. |
|  |  |  |  |  |  |  |  |
| Swing time | **Model 1** |  |  |  |  |  |  |
|  | Intercept | 8 | 14 | 0.92 (0.50 to 1.34) | F(1, 2.16) = 37.3, *p* = 0.02 | ↑ | ↑ corr. with ↑ speed |
|  | Speed* | 8 | 14 | -0.23 (-0.38 to -0.08) |  |  |  |
|  | **Model 2** |  |  |  |  |  |  |
|  | Intercept | 9 | 15 | 0.13 (-0.21 to 0.46) | F(1, 5.55) = 0.07, *p* = 0.80 |  |  |
|  | RE units (OC) | 9 | 15 | -0.08 (-0.83 to 0.67) |  | ↓ | ↓ corr. with RE expressed as OC |
|  | **Model 3** |  |  |  |  |  |  |
|  | Intercept | 9 | 15 | 0.06 (-0.40 to 0.53) | F(1, 4.07) = 25.7, *p* = 0.02 |  |  |
|  | Corrected for resting OC/EC | 9 | 15 | 0.10 (-0.54 to 0.74) |  | ↔ | Correcting did not influence the corr. |
|  | **Model 4** |  |  |  |  |  |  |
|  | Intercept | 8 | 14 | 0.43 (-2.39 to 3.24) | F(1, 1.49) = 0.20, *p* = 0.12 |  |  |
|  | CV of age | 8 | 14 | 0.01 (-0.20 to 0.17) |  | ↔ | CV of age did not influence the corr. |
|  | **Model 5** |  |  |  |  |  |  |
|  | Intercept | 8 | 14 | -0.57 (-3.46 to 2.33) | F(1, 2.57) = 0.48, *p* = 0.55 |  |  |
|  | CV of height | 8 | 14 | 0.19 (-0.79 to 1.18) |  | ↔ | CV of height did not influence the corr. |
|  | **Model 6** |  |  |  |  |  |  |
|  | Intercept | 8 | 14 | 1.42 (-2.16 to 4.99) | F(1, 2.52) = 1.49, *p* = 0.32 |  |  |
|  | CV of body mass | 8 | 14 | -0.12 (-0.46 to 0.22) |  | ↔ | CV of mass did not influence the corr. |
|  |  |  |  |  |  |  |  |
| Stride time | **Model 1** |  |  |  |  |  |  |
|  | Intercept | 7 | 12 | -0.37 (-3.14 to 2.40) | F(1, 1.42) = 0.43, *p* = 0.60 |  |  |
|  | Speed | 7 | 12 | 0.11 (-0.99 to 1.21) |  | ↔ | Speed did not influence the corr. |
|  | **Model 2** |  |  |  |  |  |  |
|  | Intercept | 7 | 12 | -0.02 (-0.72 to 0.67) | F(1, 4.29) = 0.02, *p* = 0.91 |  |  |
|  | RE units (OC) | 7 | 12 | 0.07 (-1.42 to 1.55) |  | ↔ | RE units did not influence the corr. |
|  | **Model 3** |  |  |  |  |  |  |
|  | Intercept | 7 | 12 | 0.71 (-2.22 to 3.64) | F(1, 1.41) = 0.75, *p* = 0.51 |  |  |
|  | CV of age | 7 | 12 | -0.03 (-0.23 to 0.18) |  | ↔ | CV of age did not influence the corr. |
|  | **Model 4** |  |  |  |  |  |  |
|  | Intercept | 7 | 12 | -0.64 (-3.09 to 1.80) | F(1, 2.92) = 0.55, *p* = 0.51 |  |  |
|  | CV of height | 7 | 12 | 0.19 (-0.63 to 1.01) |  | ↔ | CV of height did not influence the corr. |
|  | **Model 5** |  |  |  |  |  |  |
|  | Intercept | 7 | 12 | 1.73 (-4.28 to 7.74) | F(1, 2.11) = 1.08, *p* = 0.40 |  |  |
|  | CV of body mass | 7 | 12 | -0.15 (-0.73 to 0.44) |  | ↔ | CV of mass did not influence the corr. |
|  |  |  |  |  |  |  |  |
| Duty factor | **Model 1** |  |  |  |  |  |  |
|  | Intercept | 9 | 19 | -0.70 (-1.36 to -0.04) | F(1, 4.21) = 8.76, *p* = 0.04 |  |  |
|  | Speed | 9 | 19 | 0.19 (0.02 to 0.37) |  | ↑ | ↑ corr. with ↑ speed |
|  | **Model 2** |  |  |  |  |  |  |
|  | Intercept | 9 | 19 | 0.01 (-0.11 to 0.13) | F(1, 4.04) = 8.61, *p* = 0.04 |  |  |
|  | Corrected for resting OC/EC | 9 | 19 | -0.21 (-0.41 to -0.01) |  | ↓ | ↓ corr. with correcting for resting OC/EC |
|  | **Model 3** |  |  |  |  |  |  |
|  | Intercept | 9 | 19 | 0.24 (-0.29 to 0.76) | F(1, 1.81) = 8.54, *p* = 0.11 |  |  |
|  | CV of age |  |  | -0.01 (-0.03 to 0.01) |  | ↔ | CV of age did not influence the corr. |
|  | **Model 4** | 9 | 19 |  |  |  |  |
|  | Intercept | 9 | 19 | -0.14 (-0.59 to 0.30) | F(1, 1.56) = 0.21, *p* = 0.70 |  |  |
|  | CV of height | 9 | 19 | 0.02 (-0.22 to 0.26) |  | ↔ | CV of height did not influence the corr. |
|  | **Model 5** |  |  |  |  |  |  |
|  | Intercept | 9 | 17 | 0.45 (-0.07 to 0.96) | F(1, 1.77) = 17.3, *p* = 0.07 |  |  |
|  | CV of body mass* | 9 | 17 | -0.04 (-0.10 to 0.01) |  | ↔ | CV of mass did not influence the corr. |
|  |  |  |  |  |  |  |  |
| Stride length | **Model 1** |  |  |  |  |  |  |
|  | Intercept | 11 | 19 | 0.24 (-0.27 to 0.75) | F(2, 2.71) = 0.22, *p* = 0.82 |  |  |
|  | RE units (OC) | 11 | 19 | -0.08 (-0.68 to 0.52) |  | ↔ | RE units did not influence the corr. |
|  | Speed | 11 | 19 | -0.02 (-0.23 to 0.19) |  | ↔ | Speed did not influence the corr. |
|  | **Model 2** |  |  |  |  |  |  |
|  | Intercept | 11 | 19 | 0.30 (-0.43 to 1.03) | F(1, 2.27) = 1.10, *p* = 0.40 |  |  |
|  | Speed | 11 | 19 | -0.05 (-0.23 to 0.13) |  | ↔ | Speed did not influence the corr. |
|  | **Model 3** |  |  |  |  |  |  |
|  | Intercept | 11 | 19 | 0.18 (-0.08 to 0.44) | F(1, 6.34) = 0.29, *p* = 0.61 |  |  |
|  | RE units (OC) | 11 | 19 | -0.12 (-0.65 to 0.42) |  | ↔ | RE units did not influence the corr. |
|  | **Model 4** |  |  |  |  |  |  |
|  | Intercept | 11 | 19 | 0.10 (-0.23 to 0.43) | F(1, 1.37) = 0.05, *p* = 0.04 |  |  |
|  | Standardized shoes (NS) | 11 | 19 | 0.07 (-2.07 to 2.20) |  | ↔ | Shoe stand. did not influence the corr. |
|  | **Model 5** |  |  |  |  |  |  |
|  | Intercept | 11 | 19 | 0.15 (-0.19 to 1.07) | F(1, 1.52) = 0.93, *p* = 0.46 |  |  |
|  | Corrected for resting OC/EC | 11 | 19 | -0.21 (-1.49 to 1.07) |  | ↔ | Correcting did not influence the corr. |
|  | **Model 6** |  |  |  |  |  |  |
|  | Intercept | 11 | 19 | 0.63 (-0.14 to 1.39) | F(1, 2.99) = 3.03, *p* = 0.18 |  |  |
|  | CV of age | 11 | 19 | -0.02 (-0.06 to 0.02) |  | ↔ | CV of age did not influence the corr. |
|  | **Model 7** |  |  |  |  |  |  |
|  | Intercept | 11 | 19 | 0.08 (-1.26 to 1.41) | F(1, 4.22) = 0.00, *p* = 0.95 |  |  |
|  | CV of height | 11 | 19 | 0.01 (-0.35 to 0.36) |  | ↔ | CV of height did not influence the corr. |
|  | **Model 8** |  |  |  |  |  |  |
|  | Intercept | 11 | 19 | 0.25 (-0.70 to 1.20) | F(1, 3.55) = 0.12, *p* = 0.75 |  |  |
|  | CV of body mass | 11 | 19 | -0.01 (-0.12 to 0.09) |  | ↔ | CV of mass did not influence the corr. |
|  | **Model 9** |  |  |  |  |  |  |
|  | Intercept | 7 | 13 | 0.35 (-1.95 to 2.60) | F(1, 1.66) = 0.24, *p* = 0.68 |  |  |
|  | CV of VO_2_max | 7 | 13 | -0.03 (-0.40 to 0.33) |  | ↔ | CV of VO_2_max did not influence the corr. |
|  |  |  |  |  |  |  |  |
| Cadence | **Model 1** |  |  |  |  |  |  |
|  | Intercept | 18 | 32 | -0.27 (-0.53 to -0.01) | F(3, 5.07) = 10.6, *p* = 0.04 |  |  |
|  | Speed* | 18 | 32 | 0.07 (0.005 to 0.14) |  | ↑ | ↑ corr. with ↑ speed |
|  | RE units (OC)* | 18 | 32 | -0.26 (-0.50 to -0.03) |  | ↓ | ↓ corr. with RE expressed as OC |
|  | Standardized shoes* | 18 | 32 | -0.27 (-0.59 to 0.05) |  | ↔ | Shoe stand. did not influence the corr. |
|  | **Model 2** |  |  |  |  |  |  |
|  | Intercept | 22 | 37 | -0.21 (-0.39 to -0.03) | F(1, 4.03) = 0.79, *p* = 0.42 |  |  |
|  | Corrected for resting OC/EC | 22 | 37 | 0.16 (-0.33 to 0.64) |  | ↔ | Correcting did not influence the corr. |
|  | **Model 3** |  |  |  |  |  |  |
|  | Intercept | 22 | 37 | -0.46 (-1.12 to 0.19) | F(1, 5.26) = 0.96, *p* =0.37 |  |  |
|  | CV of age | 22 | 37 | 0.01 (-0.02 to 0.04) |  | ↔ | Age did not influence the corr. |
|  | **Model 4** |  |  |  |  |  |  |
|  | Intercept | 22 | 37 | -0.31 (-0.85 to 0.24) | F(1, 2.05) = 0.30, *p* = 0.64 |  |  |
|  | CV of height | 22 | 37 | 0.03 (-0.17 to 0.22) |  | ↔ | Height did not influence the corr. |
|  | **Model 5** |  |  |  |  |  |  |
|  | Intercept | 22 | 37 | 0.06 (-0.43 to 0.54) | F(1, 7.06) = 1.36, *p* = 0.28 |  |  |
|  | CV of body mass | 22 | 37 | -0.02 (-0.07 to 0.02) |  | ↔ | CV of mass did not influence the corr. |
|  | **Model 6** |  |  |  |  |  |  |
|  | Intercept | 9 | 17 | -0.23 (-1.04 to 0.57) | F(1, 2.57) = 0.33, *p* = 0.61 |  |  |
|  | CV of VO_2_max | 9 | 17 | 0.01 (-0.07 to 0.10) |  | ↔ | CV of VO_2_max did not influence the corr. |
|  |  |  |  |  |  |  |  |
| Vertical oscillation | **Model 1** |  |  |  |  |  |  |
|  | Intercept | 8 | 23 | 0.27 (-0.08 to 0.62) | F(2, 2.01) = 9.26, *p* = 0.10 |  |  |
|  | Standardized shoes (NS) | 8 | 23 | 0.25 (0.04 to 0.46) |  | ↑ | ↑ corr. with standardized shoes |
|  | RE units (OC) | 8 | 23 | 0.05 (-0.19 to 0.30) |  | ↔ | RE units did not influence the corr. |
|  | **Model 2** |  |  |  |  |  |  |
|  | Intercept | 8 | 23 | 0.86 (0.34 to 1.38) | F(1, 1.67) = 16.7, *p* = 0.07 |  |  |
|  | Speed | 8 | 23 | -0.15 (-0.34 to 0.04) |  | ↔ | Speed did not influence the corr. |
|  | **Model 3** |  |  |  |  |  |  |
|  | Intercept | 8 | 23 | 0.29 (0.14 to 0.45) | F(1, 2.32) = 18.9, *p* = 0.04 |  |  |
|  | Standardized shoes (NS) | 8 | 23 | 0.23 (-0.03 to 0.43) |  | ↑ | ↑ corr. with standardized shoes |
|  | **Model 4** |  |  |  |  |  |  |
|  | Intercept | 8 | 23 | 0.37 (-0.05 to 0.79) | F(1, 4.89) = 0.01, *p* = 0.93 |  |  |
|  | RE units (OC) | 8 | 23 | -0.01 (-0.04 to 0.38) |  | ↔ | RE units did not influence the corr. |
|  | **Model 5** |  |  |  |  |  |  |
|  | Intercept | 8 | 23 | 0.35 (0.20 to 0.49) | F(1, 1.15) = 3.63, *p* = 0.28 |  |  |
|  | Corrected for resting OC/EC | 8 | 23 | 0.07 (-0.28 to 0.43) |  | ↔ | Correcting did not influence the corr. |
|  | **Model 6** |  |  |  |  |  |  |
|  | Intercept | 8 | 23 | -0.24 (-2.18 to 1.70) | F(1, 2.8) = 1.19, *p* = 0.36 |  |  |
|  | CV of age | 8 | 23 | 0.02 (-0.05 to 0.10) |  | ↔ | CV of age did not influence the corr. |
|  | **Model 7** |  |  |  |  |  |  |
|  | Intercept | 7 | 22 | -0.47 (-0.94 to -0.00) | F(1, 2.67) = 49.5, *p* = 0.01 |  |  |
|  | CV of height* | 7 | 22 | 0.26 (0.13 to 0.38) |  | ↔ | CV of height did not influence the corr. |
|  | **Model 8** |  |  |  |  |  |  |
|  | Intercept | 8 | 23 | 0.49 (-0.81 to 1.78) | F(1, 2.15) = 0.14, *p* = 0.74 |  |  |
|  | CV of body mass | 8 | 23 | -0.01 (-0.12 to 0.10) |  | ↔ | CV of mass did not influence the corr. |
|  |  |  |  |  |  |  |  |
| Vertical stiffness | **Model 1** |  |  |  |  |  |  |
|  | Intercept | 10 | 18 | 0.12 (-0.37 to 0.62) | F(1, 2.04) = 17.5, *p* = 0.05 |  |  |
|  | Speed | 10 | 18 | -0.14 (-0.27 to 0.00) |  | ↔ | Speed did not influence the corr. |
|  | **Model 2** |  |  |  |  |  |  |
|  | Intercept | 10 | 18 | -0.20 (-0.58 to 0.17) | F(1, 5.15) = 2.74, *p* = 0.16 |  |  |
|  | Standardized shoes (NS) | 10 | 18 | -0.33 (-0.83 to 0.18) |  | ↔ | Shoe stan. did not influence the corr. |
|  | **Model 3** |  |  |  |  |  |  |
|  | Intercept | 10 | 18 | -0.19 (-0.56 to 0.19) | F(1, 5.00) = 4.65, *p* = 0.08 |  |  |
|  | RE units (OC) | 10 | 18 | -0.39 (-0.86 to 0.08) |  | ↔ | RE units did not influence the corr. |
|  | **Model 4** |  |  |  |  |  |  |
|  | Intercept | 10 | 18 | -0.28 (-0.68 to 0.12) | F(1, 3.70) = 0.27, *p* = 0.63 |  |  |
|  | Corrected for resting OC/EC | 10 | 18 | -0.10 (-0.64 to 0.45) |  | ↔ | Correcting did not influence the corr. |
|  | **Model 5** |  |  |  |  |  |  |
|  | Intercept | 10 | 18 | -0.27 (-1.10 to 0.56) | F(1, 4.82) = 0.04, *p* = 0.85 |  |  |
|  | CV of age | 10 | 18 | -0.00 (-0.03 to 0.03) |  | ↔ | CV of age did not influence the corr. |
|  | **Model 6** |  |  |  |  |  |  |
|  | Intercept | 9 | 16 | -0.74 (-0.96 to -0.51) | F(1, 1.55) = 92.9, *p* = 0.02 |  |  |
|  | CV of height* | 9 | 16 | 0.10 (0.04 to 0.16) |  | ↑ | ↑ corr. with higher CV of height |
|  | **Model 7** |  |  |  |  |  |  |
|  | Intercept | 10 | 18 | -0.06 (-1.77 to 1.66) | F(1, 2.09) = 0.24, *p* = 0.67 |  |  |
|  | CV of body mass | 10 | 18 | -0.02 (-0.21 to 0.17) |  | ↔ | CV of mass did not influence the corr. |
|  |  |  |  |  |  |  |  |
| Leg stiffness | **Model 1** |  |  |  |  |  |  |
|  | Intercept | 11 | 18 | 0.28 (-0.10 to 0.66) | F(1, 2.59) = 17.2, *p* = 0.03 |  |  |
|  | Speed* | 11 | 18 | -0.18 (-0.34 to -0.03) |  | ↓ | ↓ corr. with higher speeds |
|  | **Model 2** |  |  |  |  |  |  |
|  | Intercept | 11 | 18 | -0.28 (-0.65 to 0.08) | F(1, 5.15) = 0.02, *p* = 0.91 |  |  |
|  | Standardized shoes (NS) | 11 | 18 | 0.02 (-0.47 to 0.52) |  | ↔ | Shoe stan. did not influence the corr. |
|  | **Model 3** |  |  |  |  |  |  |
|  | Intercept | 11 | 18 | -0.13 (-0.38 to 0.12) | F(1, 6.99) = 2.42, *p* = 0.16 |  |  |
|  | RE units (OC)* | 11 | 18 | -0.36 (-0.90 to 0.19) |  | ↔ | Correcting did not influence the corr. |
|  | **Model 4** |  |  |  |  |  |  |
|  | Intercept | 11 | 18 | -0.26 (-0.57 to 0.05) | F(1, 1.49) = 0.40, *p* = 0.61 |  |  |
|  | Corrected for resting OC/EC | 11 | 18 | -0.09 (-0.97 to 0.78) |  | ↔ | Correcting did not influence the corr. |
|  | **Model 5** |  |  |  |  |  |  |
|  | Intercept | 11 | 18 | -0.32 (-1.05 to 0.42) | F(1, 4.34) = 0.05, *p* = 0.83 |  |  |
|  | CV of age | 11 | 18 | 0.00 (-0.03 to 0.04) |  | ↔ | CV of age did not influence the corr. |
|  | **Model 6** |  |  |  |  |  |  |
|  | Intercept | 11 | 18 | -0.60 (-1.03 to -0.17) | F(1, 1.62) = 12.4, *p* = 0.10 |  |  |
|  | CV of height | 11 | 18 | 0.08 (-0.04 to 0.20) |  | ↔ | CV of height did not influence the corr. |
|  | **Model 7** |  |  |  |  |  |  |
|  | Intercept | 11 | 18 | -0.36 (-2.19 to 1.47) | F(1, 4.18) = 0.02, *p* = 0.89 |  |  |
|  | CV of body mass | 11 | 18 | 0.01 (-0.15 to 0.17) |  | ↔ | CV of mass did not influence the corr. |

*CI,* confidence interval; CV, coefficient of variation; EE, energy cost; OC, oxygen cost RE; running economy; NS, not standardized

^a^ All *β* are expressed as Fisher’s *z* and should therefore not be interpreted as a Pearson correlation coefficient.

^b^ Omnibus test

Oxygen cost was the reference estimate for running economy units. Non-standardizes shoes were the reference estimate for shoe wear, and correction for resting or standing oxygen or energy cost was the reference estimate.

* Outliers removed
