# Supplementary material for: The Relationship Between Running Biomechanics and Running Economy: A Systematic Review and Meta-Analysis of Observational Studies
Source: Sports Med. 2024 Mar 6;54(5):1269–316. doi: 10.1007/s40279-024-01997-3 (PMC11127892; doi:10.1007/s40279-024-01997-3)
Supplement: Supplementary file 6 — Supplementary file6 (DOCX 2401 kb) [file 40279_2024_1997_MOESM6_ESM.docx]

**Online supplementary file S6 Additional figures**


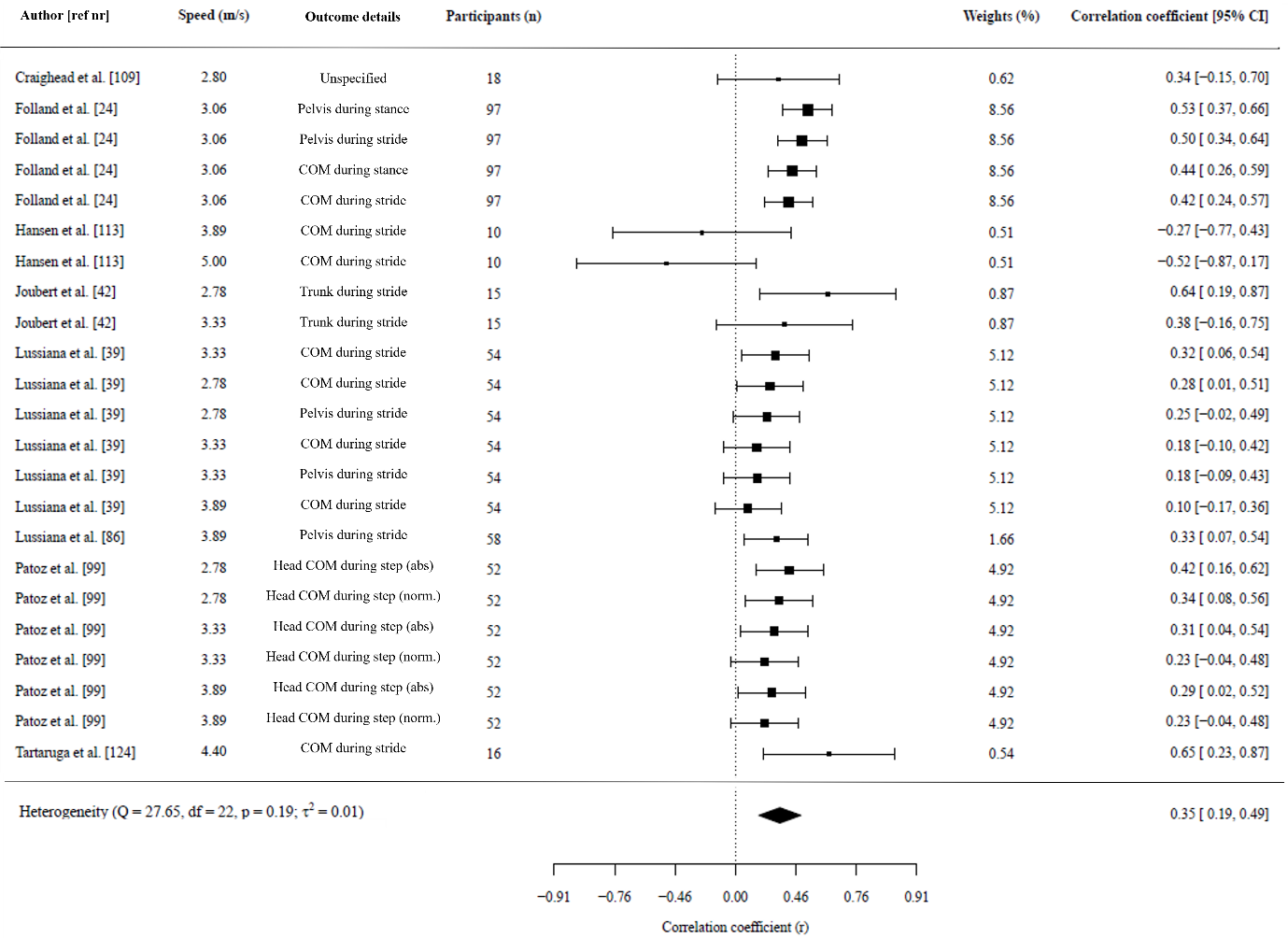


**Figure S1.** Random-effects meta-analysis of the correlation between vertical oscillation and running economy. Positive correlations indicate that higher vertical oscillation is associated with a higher oxygen or energy cost of running or that lower vertical oscillation is associated with a lower oxygen or energy cost (i.e., higher is higher or lower is lower), while negative correlations indicate that a lower vertical oscillation is associated with a higher oxygen or energy cost or that a higher vertical oscillation is associated with lower oxygen or energy cost (i.e., higher is lower or lower is higher). Note that the correlation coefficients are depicted on a non-linear scale to ensure symmetric confidence intervals after the back transformation procedure. *CI =* confidence interval; *COM* = center of mass; *abs*. = absolute; *norm*. = normalized to height


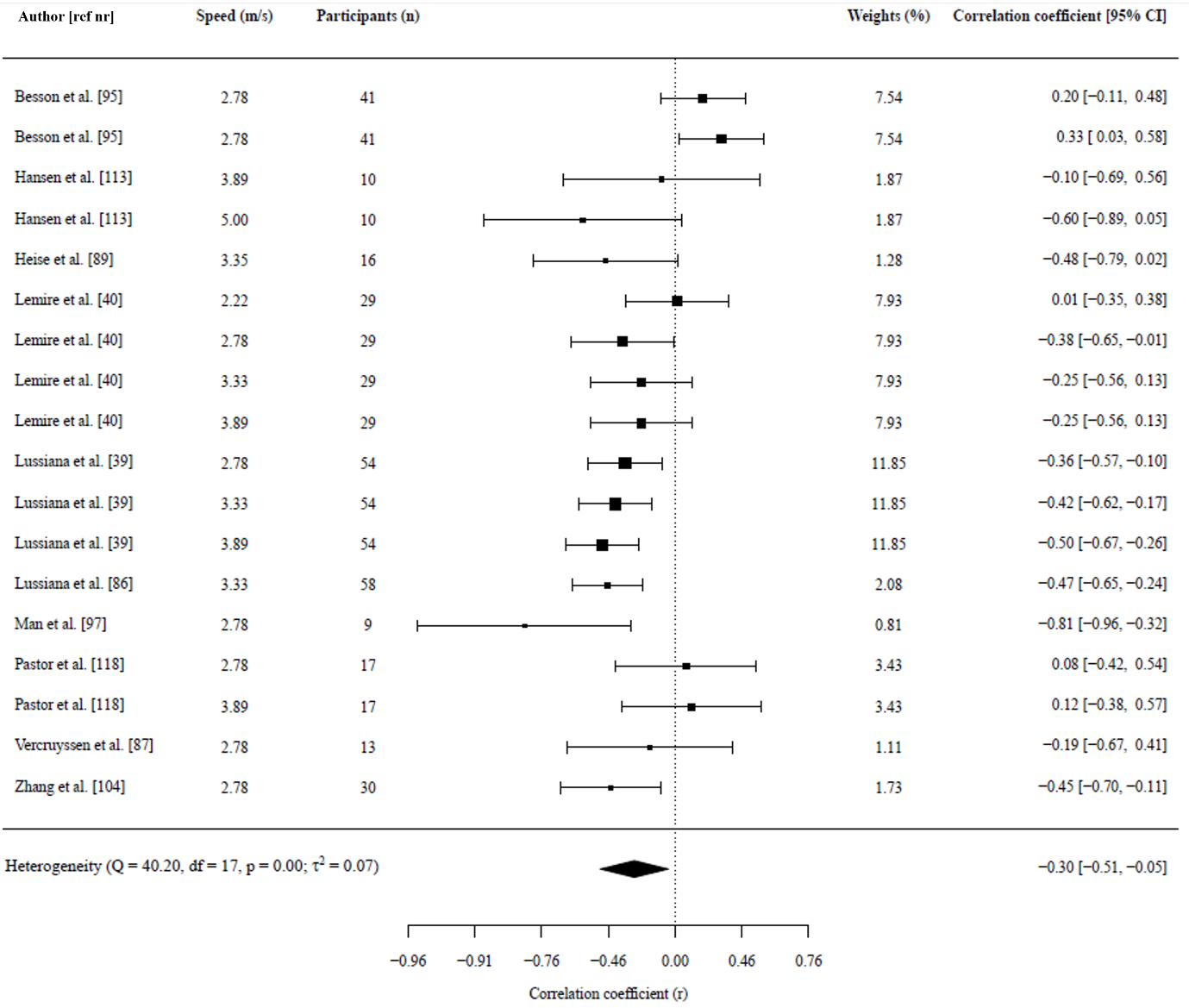


**Figure S2.** Random-effects meta-analysis of the correlation between vertical stiffness and running economy. Positive correlations indicate that a higher vertical stiffness is associated with a higher oxygen or energy cost of running or that lower stiffness is associated with a lower oxygen or energy cost (i.e., higher is higher or lower is lower), while negative correlations indicate that a lower vertical stiffness is associated with a higher oxygen or energy cost or that a higher vertical stiffness is associated with lower oxygen or energy cost (i.e., higher is lower or lower is higher). Note that the correlation coefficients are depicted on a non-linear scale to ensure symmetric confidence intervals after the back transformation procedure. *CI* confidence interval.


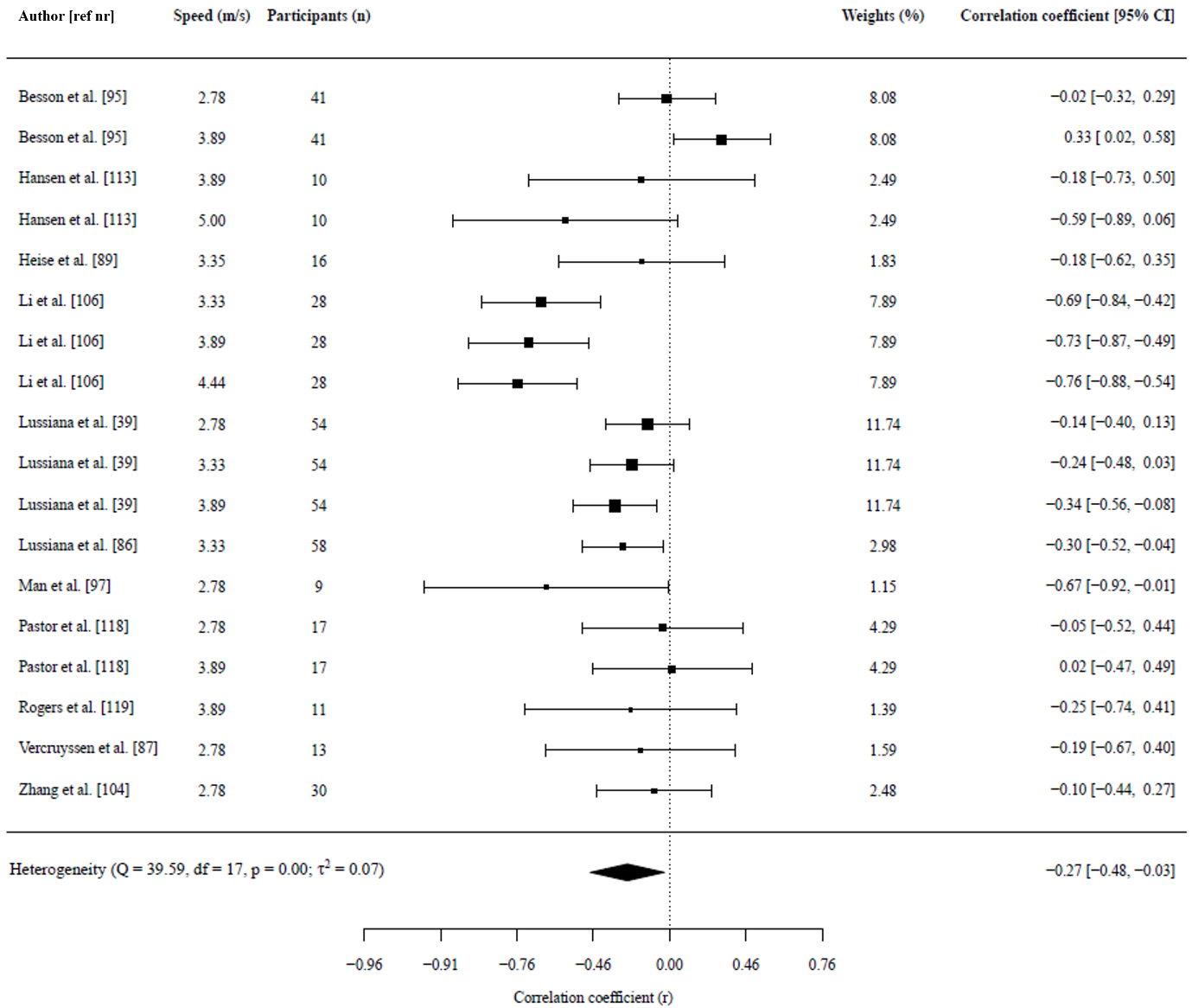


**Figure S3.** Random-effects meta-analysis of the correlation between leg stiffness and running economy. Positive correlations indicate that a higher leg stiffness is associated with a higher oxygen or energy cost of running or that lower leg stiffness is associated with a lower oxygen or energy cost (i.e., higher is higher or lower is lower), while negative correlations indicate that a lower leg stiffness is associated with a higher oxygen or energy cost or that a higher leg stiffness is associated with lower oxygen or energy cost (i.e., higher is lower or lower is higher). Note that the correlation coefficients are depicted on a non-linear scale to ensure symmetric confidence intervals after the back transformation procedure. *CI* confidence interval.

**
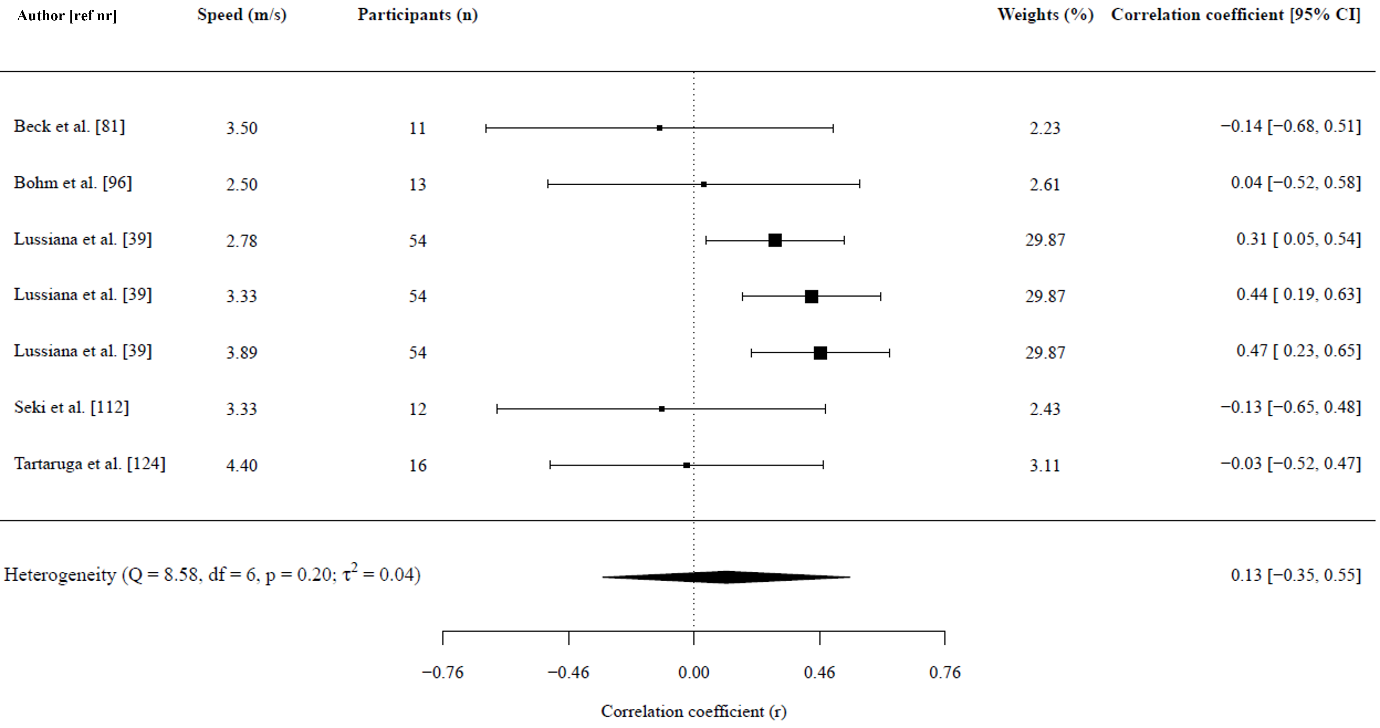
Figure S4.** Random-effects meta-analysis of the correlation between ankle angle at toe-off and running economy. Positive correlations indicate that a more plantar flexed ankle at toe-off is associated with a higher oxygen or energy cost of running or that smaller plantar flexed ankle at toe-off is associated with a lower oxygen or energy cost (i.e., higher is higher or lower is lower), while negative correlations indicate that a smaller plantar flexed ankle at toe-off is associated with a higher oxygen or energy cost or that a larger plantar flexed ankle at toe-off is associated with lower oxygen or energy cost (i.e., higher is lower or lower is higher). Note that the correlation coefficients are depicted on a non-linear scale to ensure symmetric confidence intervals after the back transformation procedure. *CI* confidence interval.


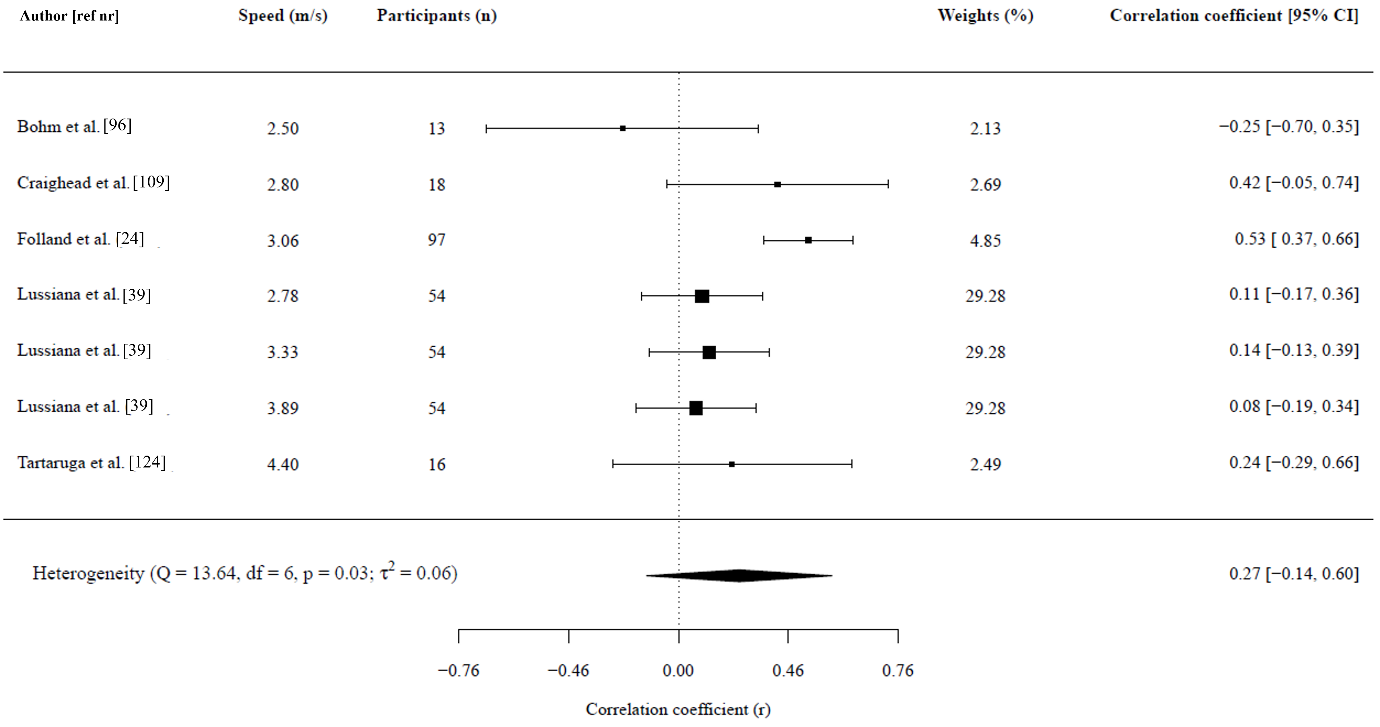


**Figure S5.** Random-effects meta-analysis of the correlation between peak knee angle during stance and running economy. Positive correlations indicate that higher peak knee flexion during stance is associated with a higher oxygen or energy cost of running or that smaller peak knee flexion during stance is associated with a lower oxygen or energy cost (i.e., higher is higher or lower is lower), while negative correlations indicate that a smaller peak knee flexion during stance is associated with a higher oxygen or energy cost or that a larger peak knee flexion during stance is associated with lower oxygen or energy cost (i.e., higher is lower or lower is higher). Note that the correlation coefficients are depicted on a non-linear scale to ensure symmetric confidence intervals after the back transformation procedure. *CI* confidence interval.


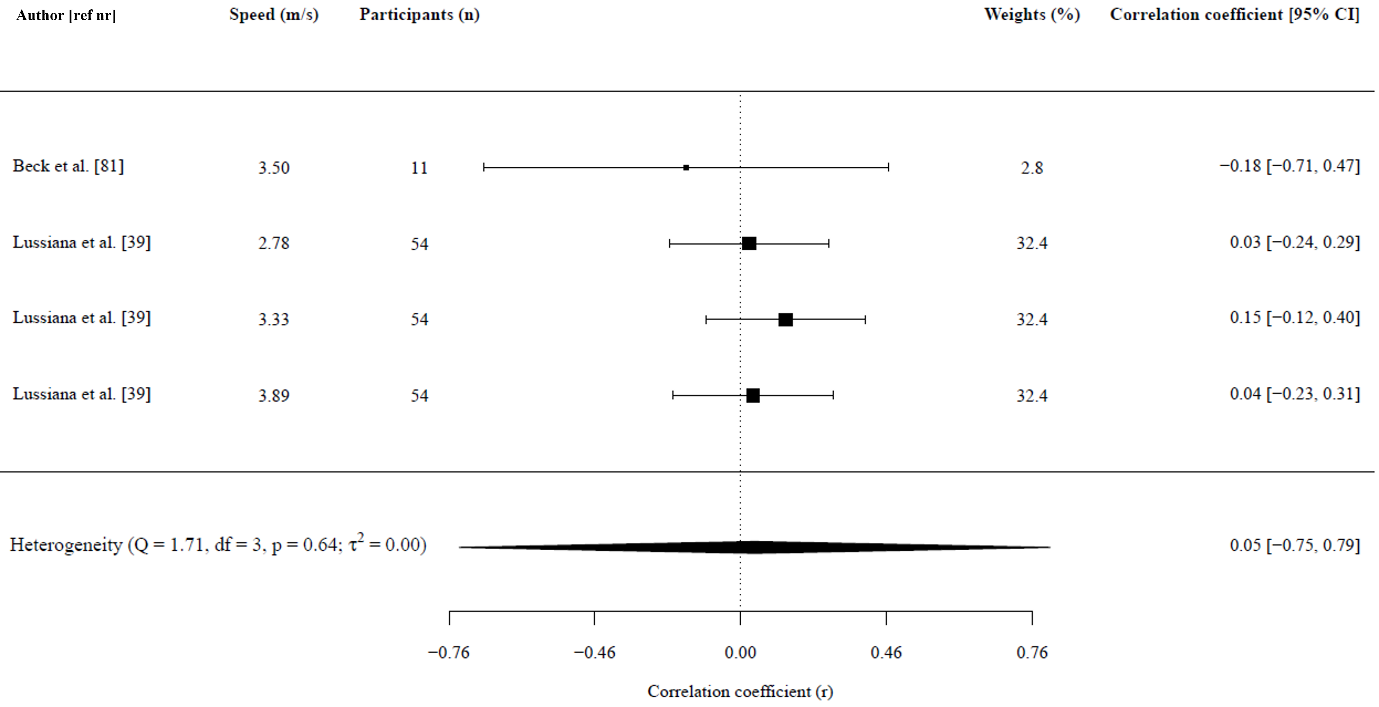


**Figure S6.** Random-effects meta-analysis of the correlation between hip angle at footstrike and running economy. Positive correlations indicate that higher hip flexion at footstrike is associated with a higher oxygen or energy cost of running or that smaller hip flexion at footstrike is associated with a lower oxygen or energy cost (i.e., higher is higher or lower is lower), while negative correlations indicate that a smaller hip flexion at footstrike is associated with a higher oxygen or energy cost or that a larger hip flexion at footstrike is associated with lower oxygen or energy cost (i.e., higher is lower or lower is higher). Note that the correlation coefficients are depicted on a non-linear scale to ensure symmetric confidence intervals after the back transformation procedure. *CI* confidence interval.


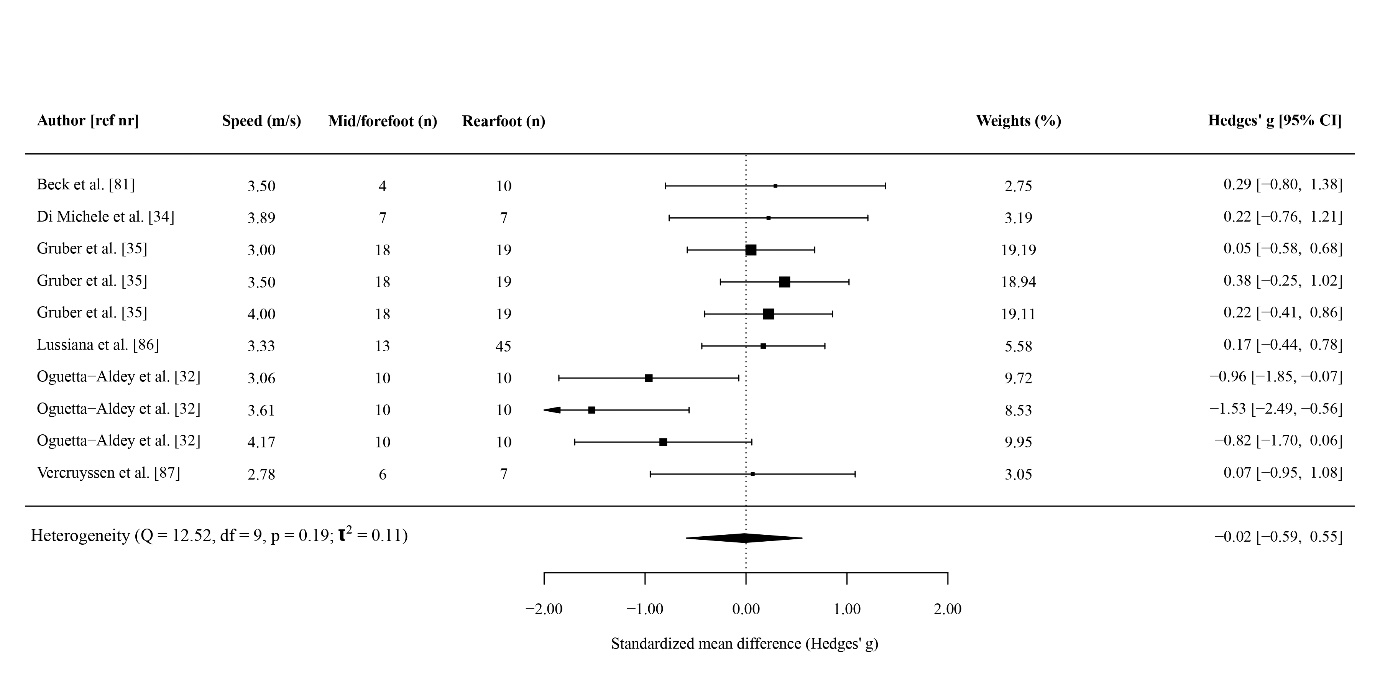


**Figure S7.** Random-effects meta-analysis of the difference in running economy between rearfoot and non-rearfoot strikers. Note that the units are standardized mean differences as opposed to correlation coefficients. Positive values indicate a higher energy cost for rearfoot strikers. *CI* confidence interval.
